# A Harmonized Visual Reading Framework for Tau PET Staging in Alzheimer Disease

**DOI:** 10.1001/jamaneurol.2026.3253

**Published:** 2026-09-21

**Authors:** Emma Ruppert, Marie R. Vermeiren, Marina Scop Medeiros, Guilherme Povala, Carolina Soares, Andreia Rocha, Alvaro de Oliveira Franco, Matheus Scarpatto Rodrigues, Markley Oliveira, Rayan Mroué, Pamela C. L. Ferreira, Guilherme Bauer-Negrini, Firoza Z. Lussier, Livia Amaral, Bruna Bellaver, Cécile Tissot, Joseph Masdeu, Dana L. Tudorascu, Thomas Karikari, David N. Soleimani-Meigooni, Juan Fortea, Val J. Lowe, Hwamee Oh, Belen Pascual, Brian A. Gordon, Pedro Rosa-Neto, Suzanne Baker, Rik Ossenkoppele, Elsmarieke van de Giessen, Tharick A. Pascoal

**Affiliations:** 1 University of Pittsburgh, Pittsburgh, Pennsylvania; 2 Department of Radiology and Nuclear Medicine, Amsterdam University Medical Center (UMC), Vrije Universiteit, Amsterdam, the Netherlands; 3 Neurology, Alzheimer Center Amsterdam, Amsterdam UMC location VUmc, Vrije Universiteit Amsterdam, Amsterdam, the Netherlands; 4 Brain Imaging, Amsterdam Neuroscience, Amsterdam, the Netherlands; 5 Graduate Program in Biological Sciences, Biochemistry Department, Universidade Federal do Rio Grande do Sul, Porto Alegre, Brazil; 6 Lawrence Berkeley National Laboratory, Berkeley, California; 7 McGill University, Montréal, Quebec, Canada; 8 Houston Methodist Research Institute, Houston, Texas; 9 Edward and Pearl Fein Memory and Aging Center, University of California, San Francisco; 10 Sant Pau Memory Unit, Biomedical Research Institute Sant Pau, Department of Neurology, Hospital de la Santa Creu y Sant Pau, Universitat Autònoma de Barcelona, Barcelona, Spain; 11 Centro de Investigación Biomédica en Red de Enfermedades Neurodegenerativas (CIBERNED), Madrid, Spain; 12 Mayo Clinic, Rochester, Minnesota; 13 Brown University, Providence, Rhode Island; 14 Washington University in St Louis, St Louis, Missouri; 15 Peter O’Donnell Jr. Brain Institute, University of Texas Southwestern Medical Centre, Dallas; 16 Neurodegeneration, Amsterdam Neuroscience, Amsterdam, the Netherlands

## Abstract

**Importance:**

Tau positron emission tomography (PET) can detect and stage Alzheimer disease pathology; however, current binary regulatory-approved visual interpretation focuses on advanced disease, limiting early detection and patient stratification.

**Objective:**

To develop and validate a harmonized tau PET visual reading framework across radiotracers that stages tau burden into 5 classes: negative, low, moderate, high, and atypical.

**Design, Setting, and Participants:**

This prospective multicenter cross-sectional study among a cohort with head-to-head tau PET acquisitions was conducted from March 2022 to August 2025 and data analyzed from February to June 2026, with blinded visual reads performed by clinicians. The study was conducted at 9 sites across North America and Europe. Of those individuals enrolled in the HEAD (Head-to-Head Harmonization of Tau Tracers in Alzheimer's Disease) study, a proportion of participants aged 50 to 90 years who completed multitracer tau PET (2-4 scans) and cognitive assessments were included, spanning cognitively unimpaired to dementia.

**Exposures:**

Tau PET with ^18^F-MK-6240 (TAUKLARIFY, n = 681), ^18^F-flortaucipir (Tauvid, n = 644), ^18^F-RO948 (n = 150), and ^18^F-PI-2620 (n = 149); all participants underwent amyloid-β (Aβ) PET and magnetic resonance imaging.

**Main Outcomes and Measures:**

The primary outcomes were interrater and intertracer agreement for 5-class tau classification; prevalence ratio (PR) vs the regulatory-approved ^18^F-flortaucipir framework; and associations with cognition, Aβ burden, and plasma phosphorylated tau 217 (p-tau217).

**Results:**

Of 822 individuals enrolled in the HEAD study, 681 participants spanning cognitively unimpaired to dementia were included. Mean (SD) age was 69.3 (8.4) years, and 367 participants (53.9%) were female. The harmonized 5-class framework achieved excellent interrater agreement (κ = 0.77-0.85) and good to excellent intertracer agreement, highest between ^18^F-MK-6240 and ^18^F-flortaucipir (κ = 0.88). Compared with the regulatory-approved framework, the harmonized classification identified more tau-positive cognitively unimpaired participants (regulatory-approved binary: 26 of 364 participants; harmonized ^18^F-flortaucipir: 49 of 364 [PR = 1.89; 95% CI, 1.31-2.70; *P* = .001]; harmonized ^18^F-MK-6240: 71 of 364 [PR = 2.73; 95% CI, 1.95-3.83; *P* < .001]) and those with mild cognitive impairment (regulatory-approved binary: 100 of 223; harmonized ^18^F-flortaucipir: 123 of 223 [PR = 1.23; 95% CI, 1.12-1.35; *P* < .001]; harmonized ^18^F-MK-6240: 142 of 223 [PR = 1.42; 95% CI, 1.27-1.59; *P* < .001]), with convergence in dementia. Tau burden classes showed stepwise worsening of cognition, Aβ PET burden, and plasma p-tau217, with 1.4-fold, 3.2-fold, and 2.5-fold differences between the low and high tau classes, respectively.

**Conclusions and Relevance:**

In this prospective multitracer cross-sectional study, a 5-class harmonized tau PET visual reading framework showed high interrater and intertracer agreement and detected more early-stage tau pathology than the regulatory-approved binary approach, with tau burden classes tracking stepwise with cognition and biomarkers. These findings support a standardized, tracer-agnostic framework for diagnostic categorization, therapeutic window identification, and clinical trial stratification.

## Key Points

**Question:**

Can a harmonized visual reading framework enable reliable, tracer-agnostic staging of tau positron emission tomography (PET) pathology for clinical use?

**Findings:**

In this multicenter cross-sectional study of 681 participants scanned with multiple tau PET radiotracers, a 5-class harmonized visual reading framework demonstrated high interrater and intertracer agreement and identified more early-stage tau pathology than the regulatory-approved approach. Tau burden classes were associated with progressively worse cognition, greater amyloid-β burden, and higher plasma phosphorylated tau 217 (p-tau217) concentrations.

**Meaning:**

A harmonized visual reading framework may enable consistent detection and staging of Alzheimer disease pathology, improve comparability across tracers, and support biomarker-informed clinical decision-making.

## Teaser

This cross-sectional study develops and validates a harmonized tau positron emission tomography (PET) visual reading framework across radiotracers that stages tau burden into 5 classes.

## Introduction

Amyloid-β (Aβ) plaques and tau neurofibrillary tangles are the defining neuropathological hallmarks of Alzheimer disease (AD).^1^ Both can be detected in vivo using positron emission tomography (PET), reflecting neuropathological stages.^2,3,4,5,6,7,8^ Currently, Aβ PET visual assessment supports diagnostic classification and eligibility for anti-Aβ therapies.^9,10^ However, although Aβ deposition is key to confirming the presence of AD pathology, it has a weak association with imminent cognitive decline.^2,11^ Indeed, in an Aβ-positive individual with cognitive decline, a negative tau PET scan raises the possibility that non-AD etiology may be the primary driver of symptoms.^3^ Tau PET, in contrast, is strongly associated with cognitive impairment and clinical presentation^12,13,14^ and can accurately distinguish AD from other dementias,^8,15,16,17,18^ positioning it to guide diagnosis, prognosis, and selection for disease-modifying therapies. Tau PET’s clinical utility depends on how scans are interpreted, yet the current regulatory-approved visual framework prioritizes specificity for advanced tau pathology, limiting the identification of early tau pathology.^10^

As tau PET expands into clinical settings, feasible and reliable visual reading methods are essential.^19,20,21,22,23,24,25^ Despite recent US Food and Drug Administration (FDA) approval of MK-6240 (TAUKLARIFY [Lantheus]; ^18^F-florquinitau),^26^ flortaucipir (Tauvid [Lilly]; previously ^18^F-T807 or ^18^F-AV1451) remains the only tau PET radiotracer with FDA- and European Medicines Agency–approved visual reading methods for adults with cognitive impairment (CI) undergoing assessment for AD.^14,25,27,28^ Tau PET’s principal advantage over fluid biomarkers is its ability to enable spatially resolved detection and regional staging of tau pathology. However, existing assessment methods do not fully exploit this capability.^29^ The current regulatory-approved framework, optimized for specificity, classifies tau PET as binary positive or negative, corresponding primarily to advanced pathology (Braak V and VI).^25,30,31^ This approach has limited sensitivity for detecting earlier tau deposition (Braak I-IV), the stage where patients are most likely to benefit from therapeutic intervention.^3,32^ This classification also does not capture the extent of tau accumulation, limiting differentiation between moderate and widespread tau deposition, despite strong associations between tau distribution and CI and expert agreement that tau PET should be used for staging in practice.^25^ Furthermore, this method was developed and validated exclusively for ^18^F-flortaucipir, limiting cross-study comparability and broader clinical adoption.

To address these gaps, we developed a harmonized visual reading framework for tau PET staging across ^18^F-flortaucipir, ^18^F-MK-6240, ^18^F-RO948, and ^18^F-PI-2620 in a large multisite cohort. The framework staged tau PET burden into 5 classes—negative, low, moderate, high, and atypical—designed to capture early tau deposition. We compared the harmonized framework with the regulatory-approved tracer-specific approach and evaluated its biological and clinical validity across the AD continuum.

## Methods

The study protocol was approved by all participating institutional review boards, and all participants provided written informed consent by self or proxy. The study followed the Strengthening the Reporting of Observational Studies in Epidemiology (STROBE) reporting guidelines.

### Participants

This cross-sectional analysis used baseline data from a portion of participants enrolled in the HEAD (Head-to-Head Harmonization of Tau Tracers in Alzheimer's Disease) study, an ongoing longitudinal multicenter cohort (ClinicalTrials.gov identifier: NCT05361382). Inclusion and exclusion criteria are in eFigure 1 in Supplement 1. Participants aged 50 to 90 years were enrolled from March 2022 to August 2025 across 9 sites in North America and Europe and underwent cognitive assessments using the National Alzheimer's Coordinating Center Uniform Data Set version 3 (UDS-3), supplemented by the Mini-Mental State Examination. Cognitively unimpaired (CU) individuals had normal cognition and Clinical Dementia Rating (CDR) of 0. Participants with mild CI (MCI) had CDR of 0.5; and participants with dementia had CDR of 1.0 or more.^33^

### Imaging

eMethods 1 in Supplement 1 details magnetic resonance imaging (MRI) and PET acquisition and processing. Aβ PET used site-specific radiotracers (eTable 1 in Supplement 1). Aβ standardized uptake value ratios (SUVRs) were calculated using whole cerebellar gray matter as the reference region and converted to Centiloids.^34^ Two pairs of raters independently determined Aβ positivity, with a tiebreaker resolving disagreements. Tau PET imaging with ^18^F-MK-6240 was acquired across all sites, while ^18^F-flortaucipir, ^18^F-PI-2620, and ^18^F-RO948 were each acquired at select sites (eTable 2 in Supplement 1). Tau SUVRs were calculated using inferior cerebellar gray matter as the reference region, and mean SUVRs were extracted from regions of interest (ROIs) using the Cerebrum Atlas.^35^ Blood sample measurements are detailed in eMethods 2 in Supplement 1.

### Harmonized Visual Reading Method

To develop the harmonized visual reading method, we defined voxel-level abnormality as more than 2 SDs above the mean of CU Aβ-negative individuals (n = 51) scanned with all 4 tau PET radiotracers (n = 131) (eTable 3 in Supplement 1), generating individual maps in Montreal Neurological Institute (MNI) 152 space. For each ROI, the proportion of participants with abnormal uptake was calculated by averaging individual maps. Then, ROIs were grouped into 7 composite regions based on frequency of abnormality across radiotracers and anatomical proximity (eFigure 2 in Supplement 1), providing a data-driven approximation of the hierarchical progression of tau pathology. Additional thresholds had no impact on composite assignments (eFigure 3, eTable 4 in the Supplement). Regions susceptible to off-target or spillover binding were excluded for all radiotracers (eFigure 4 in Supplement 1).^36,37,38,39^

**Figure 1.**
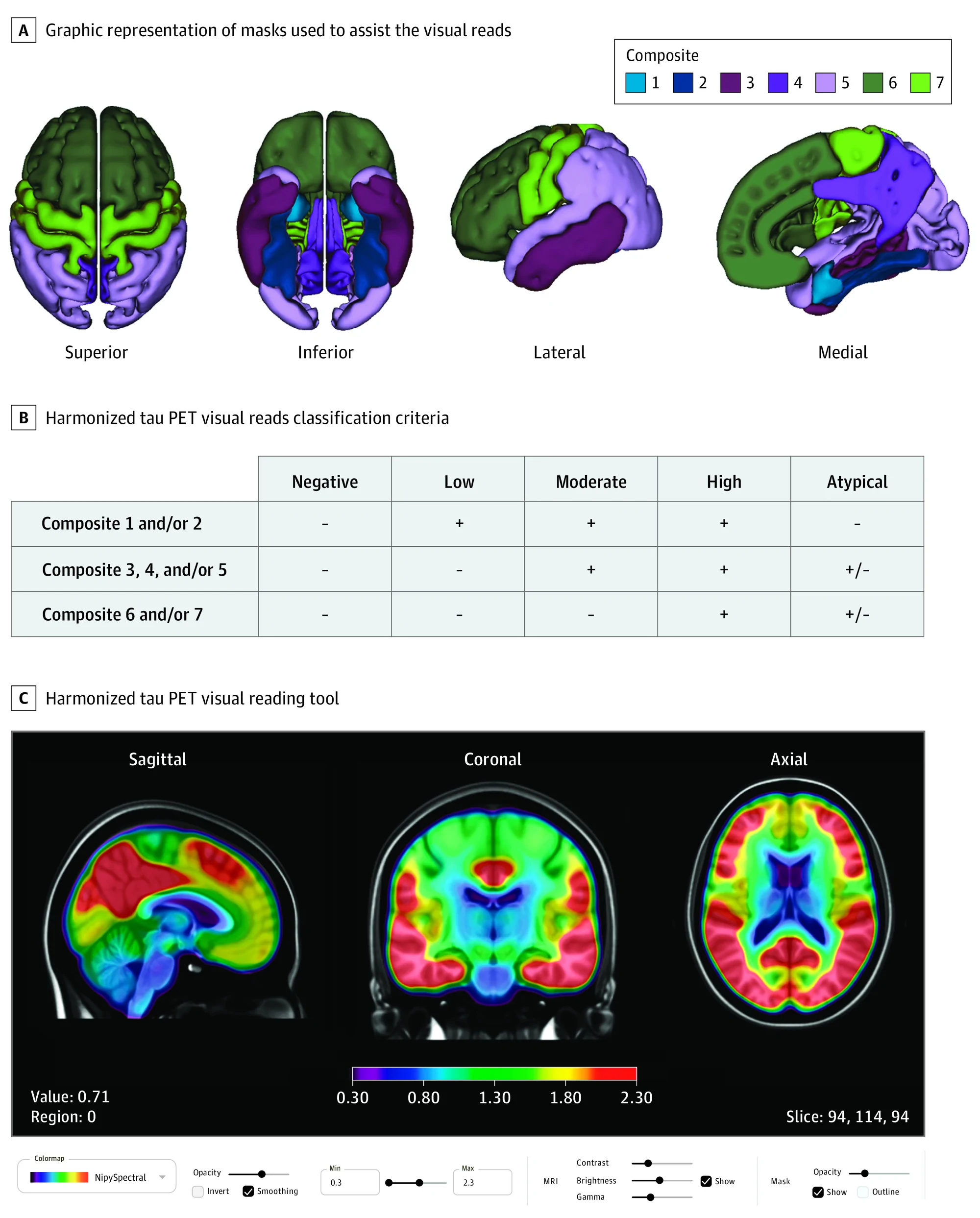
Visualization of Brain Regions, Classification Schematic, and Reading Tool Showing Harmonized Framework for Tau Positron Emission Tomography (PET) Visual Reading and Classification A, Seven composite regions used for visual assessment, displayed in superior, inferior, lateral, and medial views. The composite cortical regions were (1) amygdala and entorhinal; (2) fusiform gyrus; (3) middle and inferior temporal; (4) posterior cingulate and precuneus; (5) inferior parietal, lateral occipital, superior temporal, superior parietal, and supramarginal; (6) anterior cingulate, middle frontal, superior frontal, inferior frontal, and orbitofrontal; and (7) precentral, paracentral, and postcentral. Composites are color coded by stage: early (composites 1 and 2, in blue), moderate (composites 3, 4, and 5, in purple), and late (composites 6 and 7, in green). B, Stepwise classification algorithm for tau burden staging. Scans without abnormal tracer uptake in any composite are classified as tau PET negative. Scans with abnormal uptake are classified as tau PET positive and further categorized as low (uptake limited to composites 1 and/or 2), moderate (low criteria met plus uptake in at least 1 of the composites 3, 4, and/or 5), high (moderate criteria met plus uptake in at least 1 of the composites 6 and/or 7), or atypical (uptake in any of the composites 3-7 without involvement of composites 1 or 2). Panel C shows a freely available web-based tool (https://visualreading.unitau.app/) that visually guides raters through the 7 composite regions. The tool processes all data locally on the user’s computer, and data are not transferred to external servers.

We then developed the following 5-class classification scheme based on the pattern of composite region involvement: negative, low, moderate, high, and atypical tau burden (Figure 1). For the harmonized framework, binary tau positivity corresponds to any classification other than negative. This framework reflects established spatial progression patterns of tau pathology in AD^31^ and enables both early detection and disease staging. To standardize visual assessment, we developed a web-based visual reading platform that presents tau PET images coregistered with MRI and guides raters through binary classification (positive or negative) of each composite. To aid raters, the platform overlaid composite-specific masks (refined using 3D Slicer^40^) (eFigure 5 in Supplement 1). This standardized interface ensures consistency across raters and reduces variability associated with different software platforms (eFigure 6 in Supplement 1). On completion of all composite ratings, raters assigned a confidence level from 1 to 5 and could add commentary. The platform then assigns the corresponding 5-class classification. Three clinician-scientist raters (E.R., M.R.V., E.v.d.G.) with neuroimaging experience and 3, 2, and 5 years of tau PET reading experience, respectively, evaluated all scans following standardized instructions (eMethods 3 in Supplement 1). Raters were blinded to demographic, clinical, and biomarker data and other raters’ assessments. Washout periods between reading sessions minimized recall bias for participants with multiple scans. For both binary positivity and 5-class classification, majority vote was applied, with discordant cases across all raters resolved by consensus. Three clinician-scientist raters (E.R., M.S.M., A.d.O.F.) also assessed ^18^F-flortaucipir images blinded using the regulatory-approved visual reading framework, with majority vote applied.^14,25^

### Statistical Analysis

Interrater agreement was quantified using unweighted Cohen κ for pairwise comparisons and Fleiss κ for 3-rater comparisons. Intertracer agreement was quantified using unweighted Cohen κ for binary positivity and weighted Cohen κ for 5-class classification. Kappas were interpreted as poor (<0.40), fair (0.40-0.59), good (0.60-0.74), and excellent (≥0.75).^41^ To assess robustness, sensitivity analyses were performed restricted to CI and Aβ-positive subgroups, participants with all 4 radiotracers (n = 131), participants excluded from the initial composite-development sample, and participant subgroups stratified by scan interval. Participants with tau PET data available for fewer than 4 radiotracers were included in all analyses for which they had data.

Comparisons between the harmonized and regulatory-approved frameworks were restricted to participants with both ^18^F-flortaucipir and ^18^F-MK-6240 tau PET (n = 644). To compare tau PET positivity prevalence between frameworks, generalized estimating equations with a Poisson distribution, log link, and exchangeable within-person correlation structure were fitted with Centiloid as a covariate to estimate prevalence ratios (PRs). Differences across clinical groups were evaluated using Wald interaction tests and estimated stratum-specific PRs with 95% confidence intervals using marginal means. To estimate positivity as a function of age, we fit generalized additive models with a binomial link function and age modeled as a cubic regression spline with knots at 60, 70, and 80 years. To compare Montreal Cognitive Assessment (MoCA) scores, Centiloid values, and plasma phosphorylated tau 217 (p-tau217) across tau burden classes, linear models adjusted for age and sex were fitted separately for ^18^F-flortaucipir and ^18^F-MK-6240. Pairwise contrasts were estimated using marginal means with Tukey correction for multiple comparisons. Domain-specific cognitive composites were derived from the UDS-3 neuropsychological battery and *z*-scored for age, sex, and education.^42^ Missing data were minimal (Table). Participants missing outcome data were excluded only from analyses involving those measures. All tests were 2-sided with a significance threshold of *P* < .05, and all analyses were conducted in R version 4.4.0 (R Foundation).

**Table.** Demographics and Key Characteristics of HEAD Study Participants (n = 681)

Characteristic	No. (%)^a^		
	CU (n = 369)	MCI (n = 245)	Dementia (n = 67)
Age, mean (SD), y	67.2 (8.3)	71.8 (7.7)	72.0 (8.7)
Sex			
Female	224 (60.7)	112 (45.7)	31 (46.3)
Male	145 (39.3)	133 (54.3)	36 (53.7)
Education, mean (SD), y	16.1 (2.5)	15.5 (3.4)	15.5 (2.9)
Race^b^			
Asian	4 (1.1)	7 (2.9)	0
Black or African American	15 (4.1)	5 (2.0)	3 (4.5)
White	348 (94.3)	231 (94.3)	63 (94.0)
Other or unknown^c^	2 (0.5)	2 (0.8)	1 (1.5)
Ethnicity^b^			
Hispanic or Latino	7 (1.9)	13 (5.3)	6 (9.0)
Non-Hispanic or Latino	362 (98.1)	232 (94.7)	61 (91.0)
APOE ε4			
Carrier	114 (31.2)	123 (50.2)	42 (63.6)
Noncarrier	251 (68.8)	122 (49.8)	24 (36.4)
MMSE score, mean (SD)	28.7 (1.5)	26.4 (3.1)	20.9 (6.1)
MoCA score, mean (SD)	27.1 (2.2)	22.7 (4.2)	15.8 (6.5)
Aβ PET visual read			
Negative	283 (76.7)	90 (36.7)	11 (16.4)
Positive	86 (23.3)	155 (63.3)	56 (83.6)
Centiloid, mean (SD)	14.5 (32.4)	57.6 (55.1)	90.6 (56.3)
Plasma p-tau217, mean (SD), pg/mL	0.4 (0.3)	0.8 (0.6)	1.4 (0.8)
Tau PET images			
^18^F-MK-6240	369 (100)	245 (100)	67 (100)
^18^F-Flortaucipir	364 (98.6)	223 (91.0)	57 (85.1)
^18^F-RO948	71 (19.2)	60 (24.5)	19 (28.4)
^18^F-PI-2620	71 (19.2)	61 (24.9)	17 (25.4)

Abbreviations: Aβ, amyloid-β; APOE ε4, apolipoprotein ε4 allele; CU, cognitively unimpaired; MCI, mild cognitive impairment; MMSE, Mini-Mental State Examination; MoCA, Montreal Cognitive Assessment; p-tau217, phosphorylated tau 217; PET, positron emission tomography.

^a^ Missing values: education (n = 4), APOE ε4 (n = 5), MMSE score (n = 2), MoCA score (n = 3), and plasma p-tau217 (n = 13).

^b^ Self-reported following the Uniform Data Set version 3 A1 package.

^c^ Included individuals identifying as Ashkenazi Jewish, Filipino, Latin-American, and unknown.

## Results

Of 822 consented individuals, 74 withdrew, 10 had incomplete data, and 57 were excluded (eFigure 1 in Supplement 1). Of 681 participants aged 50 to 90 years, mean (SD) age was 69.3 (8.4) years, and 367 participants (53.9%) were female. ^18^F-MK-6240 was available in 681 participants (100%), ^18^F-flortaucipir in 644 (94.6%), ^18^F-RO948 in 150 (22.0%), and ^18^F-PI-2620 in 149 (21.9%). Mean (SD) interval between PET scans was 41.3 (46.6) days. Cohort characteristics are summarized in the Table and in eTables 5 through 8 in Supplement 1.

### Agreement Rates

**Figure 2.**
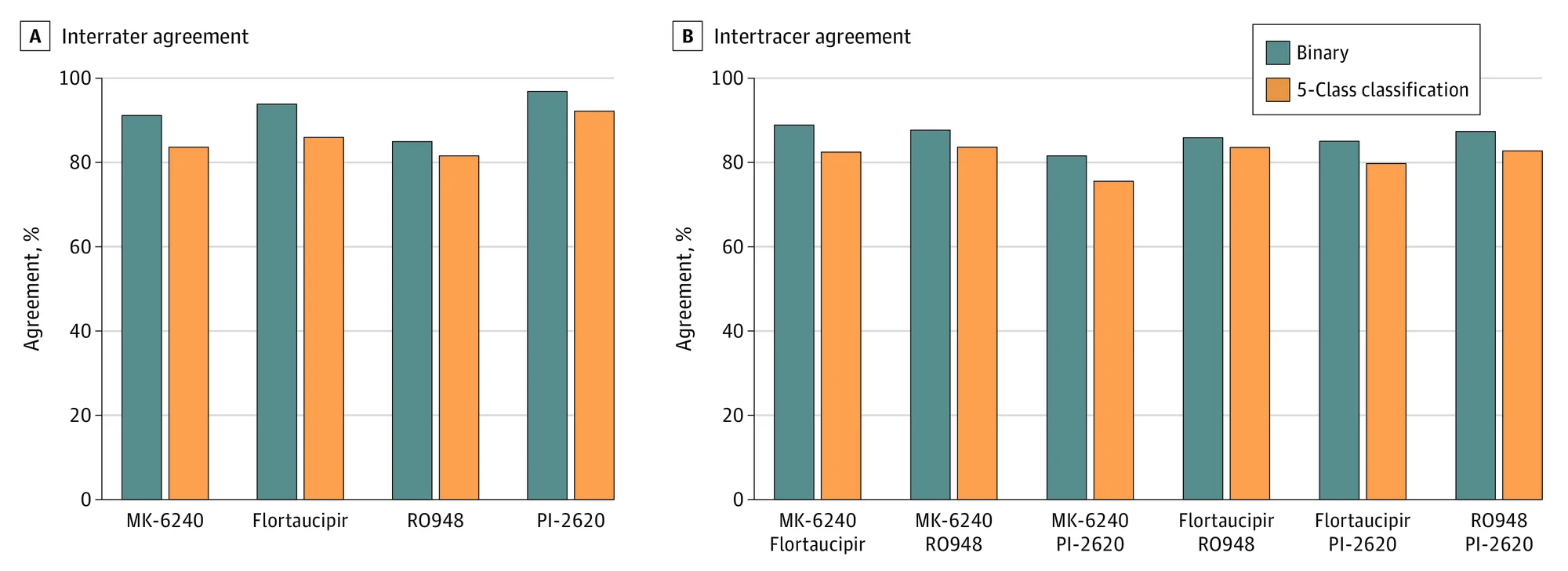
Bar Graphs Showing Interrater and Intertracer Agreement for Harmonized Tau Positron Emission Tomography Visual Reading A, Interrater agreement for each radiotracer. Grouped bars show percentage agreement for binary positivity and 5-class tau burden classification based on agreement across all 3 raters. Sample sizes were: ^18^F-MK-6240 (n = 681), ^18^F-flortaucipir (n = 644), ^18^F-RO948 (n = 150), and ^18^F-PI-2620 (n = 149). B, Intertracer agreement for each radiotracer pair. Grouped bars show percentage agreement for binary positivity and 5-class framework based on agreement across all 3 raters. Sample sizes were: ^18^F-MK-6240/^18^F-flortaucipir (n = 644), ^18^F-MK-6240/^18^F-RO948 (n = 150), ^18^F-MK-6240/^18^F-PI-2620 (n = 149), and all other pairs (n = 131 each).

Interrater agreement for tau PET binary positivity and 5-class classification was excellent across radiotracers (κ = 0.77-0.93) (Figure 2A). Binary and 5-class agreement ranged from good to excellent in Aβ-positive, CI, and 4-tracer subsamples (eTable 9 in Supplement 1). Mean SUVR of tau distribution across classification groups are provided in eFigures 7 through 11 in Supplement 1. Composite-level agreement and rater confidence were notably lower for ^18^F-PI-2620, particularly for composite 1 (eFigures 12 and 13 in Supplement 1). Exclusion of composite 1 for ^18^F-PI-2620 improved interrater agreement while largely preserving intertracer agreements (eTable 10 and 11 in Supplement 1).

Intertracer agreement (Figure 2B) was highest between ^18^F-MK-6240 and ^18^F-flortaucipir for both binary positivity (κ = 0.76) and 5 classes (κ = 0.88). Across all tracer pairs, binary agreement ranged from 81.2% to 88.5% and 5-class agreement ranged from 75.2% to 83.3%, with comparable ranges across Aβ-positive, CI, and 4-tracer subsamples and across scan intervals (eTable 12 and 13 in Supplement 1). Disagreements across radiotracers showed notable asymmetries (eFigure 14 in Supplement 1): ^18^F-MK-6240 detected positivity more frequently than other tracers (eFigure 15 and 16 in Supplement 1), while ^18^F-PI-2620 was more often negative when other tracers were positive, consistent with the removal of composite 1 (eTable 11-14 in Supplement 1). There was no convergence of atypical classification across tau PET tracers. Among participants with an atypical classification (n = 15) (eFigure 17, eTable 15 in Supplement 1), 10 of 11 Aβ-positive individuals (90%) showed a typical pattern of tau positivity on all other tracers. In contrast, all Aβ-negative individuals were tau-negative on all other tracers.

### Tau PET Positivity and Staging

Binary tau PET positivity increased progressively from CU to MCI to dementia across radiotracers, with rates highest for ^18^F-MK-6240 and lowest for ^18^F-PI-2620 (eTable 16 in Supplement 1). Five-class classification showed that higher tau burden classes were more prevalent with greater CI and Aβ positivity across all radiotracers (eFigure 18 in Supplement 1). CU participants constituted most of the negative classification (^18^F-MK-6240: 296 [74.7%]; ^18^F-flortaucipir: 315 [74.1%]; ^18^F-RO948: 67 [69.1%]; ^18^F-PI-2620: 70 [58.8%]) (eFigure 19 in Supplement 1). Among the positive stages, the proportion of participants with CI increased progressively from low to high, with high tau burden composed almost exclusively of individuals with CI (^18^F-MK-6240: 110 [94.8%]; ^18^F-flortaucipir: 88 [95.7%]; ^18^F-RO948: 22 [100%]; and ^18^F-PI-2620: 17 [100%]).

**Figure 3.**
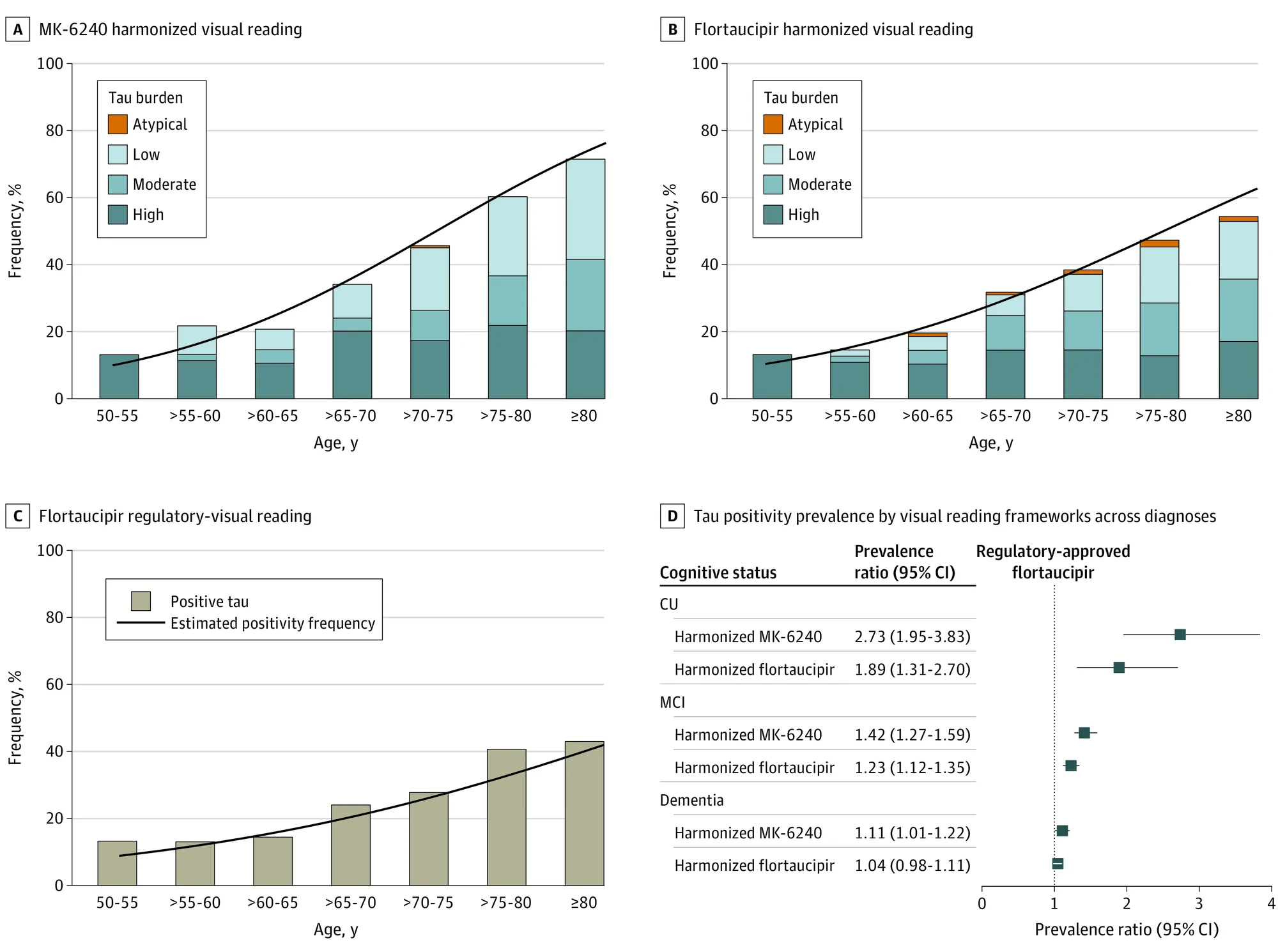
Bar and Line Graphs Showing Tau Positron Emission Tomography (PET) Positivity by Age and Clinical Diagnosis Across Visual Reading Frameworks Stacked bars show the proportion of tau-positive participants within 5-year age bins, stratified by tau burden class (low, moderate, high, and atypical) for the harmonized framework with ^18^F-MK-6240 (A) and ^18^F-flortaucipir (B) and by binary classification for the regulatory-approved framework with ^18^F-flortaucipir (C). Superimposed curves show estimated positivity frequency as a function of age, derived from generalized additive models. D, Prevalence ratios (PRs) comparing tau PET positivity for harmonized ^18^F-MK-6240 and harmonized ^18^F-flortaucipir reads relative to regulatory-approved ^18^F-flortaucipir reads, stratified by clinical diagnosis. Points represent stratum-specific PRs adjusted for Centiloid values with 95% confidence intervals. PR >1 indicates that the harmonized framework classified more participants as tau positive than the regulatory-approved framework. For harmonized ^18^F-flortaucipir vs regulatory-approved: cognitively unimpaired (CU), PR = 1.89 (95% CI, 1.31-2.70; *P* = .001); mild cognitive impairment (MCI), PR = 1.23 (95% CI, 1.12-1.35; *P* < .001); and dementia, PR = 1.04 (95% CI, 0.98-1.11; *P* = .16). For harmonized ^18^F-MK-6240 vs regulatory-approved: CU, PR = 2.73 (95% CI, 1.95-3.83; *P* < .001); MCI, PR = 1.42 (95% CI, 1.27-1.59; *P* < .001); and dementia, PR = 1.11 (95% CI, 1.01-1.22; *P* = .03). Analyses were restricted to participants with both ^18^F-MK-6240 and ^18^F-flortaucipir scans (n = 644).

Tau positivity increased with age for both ^18^F-MK-6240 and ^18^F-flortaucipir, regardless of visual reading framework (Figure 3). Harmonized reads identified more tau-positive individuals than the regulatory-approved framework, particularly in earlier stages (eTable 17 in Supplement 1). After Centiloid adjustment, harmonized ^18^F-flortaucipir was more likely to classify individuals as tau-positive than the regulatory-approved framework (219 of 644 [34.0%] vs 171 of 644 [26.6%]; PR = 1.28; 95% CI, 1.18-1.40), as was harmonized ^18^F-MK-6240 (263 of 644 [40.8%] vs 171 of 644 [26.6%]; PR = 1.54; 95% CI, 1.40-1.69). This effect varied by clinical class, with the largest divergence in CU (regulatory-approved ^18^F-flortaucipir, 26 of 364; harmonized ^18^F-flortaucipir, 49 of 364 [PR = 1.89; 95% CI, 1.31-2.70; *P* = .001]; harmonized ^18^F-MK-6240, 71 of 364 [PR = 2.73; 95% CI, 1.95-3.83; *P* < .001]) and MCI (100 of 223; 123 of 223 [PR = 1.23; 95% CI, 1.12-1.35; *P* < .001] and 142 of 223 [PR = 1.42; 95% CI, 1.27-1.59; *P* < .001]; respectively). In dementia, positivity rates converged (45 of 57, 47 of 57, and 50 of 57, respectively).

### Stepwise Association With CI, Cognition, and Biomarkers

**Figure 4.**
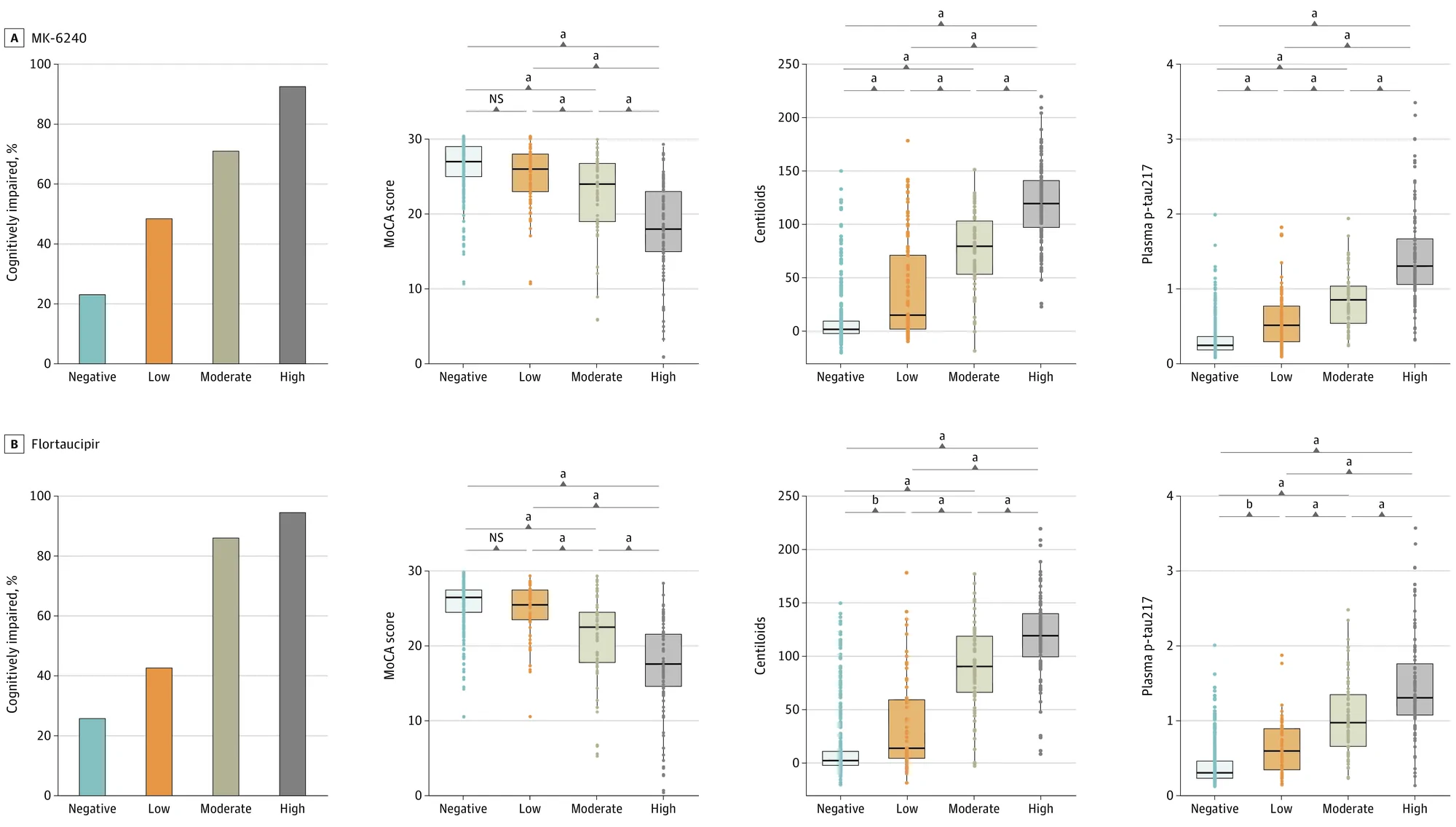
Bar Graphs and Box Plots Showing Clinical Characterization of Tau Burden Classes Bar charts show the percentage of cognitively impaired participants (mild cognitive impairment or dementia) within each tau burden class (negative, low, moderate, and high) for ^18^F-MK-6240 (A) and ^18^F-flortaucipir (B). Box plots show Montreal Cognitive Assessment (MoCA) scores, Centiloid values, and plasma phosphorylated tau 217 (p-tau217) levels stratified by harmonized tau burden class for each radiotracer. Participants classified as atypical were excluded due to small sample size (^18^F-MK-6240, n = 1; ^18^F-flortaucipir, n = 7). Boxes represent the interquartile range, horizontal lines indicate the median, whiskers extend to 1.5 × the interquartile range, and individual data points are shown. Pairwise comparisons were estimated using linear models adjusted for age and sex, with Tukey correction for multiple comparisons. NS indicates not significant. ^a^Significant at *P* < .001. ^b^Significant at *P* < .01.

Among 644 participants with ^18^F-MK-6240 and ^18^F-flortaucipir, higher tau burden classes in the harmonized framework were associated with progressively greater proportions of CI, poorer cognitive performance, higher Aβ load, and higher plasma p-tau217 (Figure 4). The prevalence of CI was lowest in the negative class for both radiotracers and increased stepwise across subsequent classes. MoCA scores did not differ between negative and low tau burden groups for ^18^F-MK-6240 (adjusted mean difference, 0.7; 95% CI, −0.4 to 1.9) or ^18^F-flortaucipir (adjusted mean difference, 0.5; 95% CI, −0.9 to 1.9). In subsequent classes, MoCA scores declined progressively: with ^18^F-MK-6240, by 2.5 points (95% CI, 0.9-4.1) from low to moderate and 4.6 points (95% CI, 3.0-6.2) from moderate to high; and with ^18^F-flortaucipir, by 3.3 points (95% CI, 1.6-5.0) and 4.4 points (95% CI, 2.8-6.0), respectively. Domain-specific cognitive composites showed a similar stepwise pattern (eTables 18 and 19 in Supplement 1).

Centiloid values (CL) increased across tau burden classes with both radiotracers. With ^18^F-MK-6240, adjacent-class differences were 24.4 CL (95% CI, 14.7-34.1), 35.3 CL (95% CI, 21.4-49.2), and 48.1 CL (95% CI, 34.4-61.9); and with ^18^F-flortaucipir, 17.2 CL (95% CI, 4.9-29.6), 53.0 CL (95% CI, 37.3-68.7), and 32.8 CL (95% CI, 18.6-47.0). Plasma p-tau217 followed the same pattern. With ^18^F-MK-6240, adjacent-class differences were 0.2 pg/mL (95% CI, 0.07-0.29) from negative to low, 0.3 pg/mL (95% CI, 0.12-0.44) from low to moderate, and 0.6 pg/mL (95% CI, 0.46-0.77) from moderate to high. For ^18^F-flortaucipir, the corresponding differences were 0.2 pg/mL (95% CI, 0.04-0.32), 0.4 pg/mL (95% CI, 0.24-0.59), and 0.4 pg/mL (95% CI, 0.29-0.61). Across visual reading frameworks, tau-positive participants had lower MoCA scores and higher Aβ burden and plasma p-tau217 levels than tau-negative participants (eFigure 20 in Supplement 1). Participants classified as tau positive only by the harmonized framework had significantly higher CLs and plasma p-tau217 levels than those negative on both frameworks, whereas participants positive only on the regulatory-approved framework did not show consistent biomarker elevation (eFigure 21 in Supplement 1).

## Discussion

We developed and validated a tracer-agnostic, harmonized visual reading framework for tau PET applicable across ^18^F-MK-6240, ^18^F-flortaucipir, ^18^F-RO948, and ^18^F-PI-2620, supported by a free web-based reading tool. This 5-class framework achieved high interrater and intertracer agreement, detected tau-positive individuals at earlier stages than the regulatory-approved approach, and produced tau burden classifications that showed stepwise associations with cognitive decline, Aβ load, and plasma p-tau217.

Interrater and intertracer agreement ranged from good to excellent across radiotracers, meeting or exceeding those reported for existing frameworks, while extending classification to 5 classes of tau burden.^21,22,25^ Composite-level agreement was notably lower for ^18^F-PI-2620 in composite 1 (amygdala, entorhinal cortex), where raters reported low confidence in distinguishing tau binding from off-target signal in the adjacent cavernous sinus.^39^ This led to the exclusion of composite 1 from ^18^F-PI-2620 classification criteria and explains the lower overall confidence observed for this tracer. Across tracers, ^18^F-MK-6240 more often yielded positive classifications when others were negative, possibly reflecting earlier tau detection,^43^ while ^18^F-PI-2620 was more frequently classified as negative, likely due to its higher positivity threshold after composite 1 exclusion. Consequently, sensitivity estimates for ^18^F-PI-2620 are not directly comparable to those of the other 3 tracers and should be interpreted cautiously. Despite these tracer-specific differences, overall high interrater and intertracer agreement support the feasibility of standardized reporting using a single framework.

The regulatory-approved visual framework for ^18^F-flortaucipir classifies scans as positive or negative, with positivity corresponding to advanced tau pathology.^25,27^ This approach is not sensitive to early tau deposition and does not characterize the extent of tau accumulation, limiting its ability to aid in biomarker-informed treatment decisions.^3,12,32^ The 5-class harmonized framework addressed both limitations. Compared with the regulatory-approved method, the harmonized framework classified significantly more participants as tau positive among CU participants (^18^F-flortaucipir, PR = 1.89; ^18^F-MK-6240, PR = 2.73) and among those with MCI (^18^F-flortaucipir, PR = 1.23; ^18^F-MK-6240, PR = 1.42), while positivity rates converged in dementia. The difference between harmonized and regulatory-approved rates widened with age, consistent with early-stage tau pathology falling below the regulatory-approved detection threshold. Participants classified as positive only by the harmonized framework had higher Aβ load and higher plasma p-tau217 levels than those negative by both frameworks, supporting the biological relevance of these additional detections.

Beyond binary classification, the 5-class tau burden classification captures the spatial progression of tau pathology in a clinically meaningful manner. Higher tau burden classes were associated with higher proportions of CI, poorer cognitive performance, higher Aβ load, and higher plasma p-tau217 in a stepwise manner across ^18^F-MK-6240 and ^18^F-flortaucipir. MoCA scores did not differ between negative and low-burden groups, consistent with early stages of AD in which tau deposition precedes measurable cognitive decline. Cognition declined progressively in subsequent stages, with reductions in MoCA scores of 2.5 to 3.3 points from low to moderate and 4.4 to 4.6 points from moderate to high burden. CLs and plasma p-tau217 increased across all burden classes, confirming that the classification tracks with both Aβ accumulation and phosphorylated tau levels.

In practice, the 5-class classification may inform diagnostic categorization, prognosis, and clinical trial eligibility more precisely than the regulatory-approved binary tau framework. A negative classification in a symptomatic individual may redirect evaluation toward non-AD etiologies.^3^ Among tau-positive individuals, the low and moderate burden categories identify individuals with early tau pathology, elevated Aβ burden, and absent or mild CI, representing a stage at which disease-modifying therapies may be most effective.^3^ Notably, some of these individuals would be classified as tau negative by the regulatory-approved framework, potentially missing an opportunity for early intervention. Individuals with high tau burden, who exhibited the greatest CI and biomarker abnormalities, carry the highest clinical risk and may have limited response to anti-Aβ therapies.^32^ Thus, graded tau staging may enable more refined participant selection and stratification in clinical trials than binary inclusion criteria by identifying individuals with early tau pathology who would otherwise be classified as tau negative despite representing optimal candidates for early intervention.

### Limitations

This study has several limitations. Although tau PET radiotracers are used as in vivo markers of neurofibrillary tangle pathology, with strong correspondence to postmortem findings,^7,8^ direct neuropathological validation in this study was not performed. Primary clinical validation focused on ^18^F-MK-6240 and ^18^F-flortaucipir, the most widely used radiotracers, whereas sample sizes were smaller for ^18^F-PI-2620 and ^18^F-RO948, reflecting limited availability of participants imaged with all 4 radiotracers and potentially reducing the precision of agreement estimates. The harmonized framework was developed and evaluated in the same cohort and will require external validation in independent datasets for generalizability. The cross-sectional design precludes assessment of whether tau burden classification predicts longitudinal cognitive decline or progression across burden stages, which should be addressed in longitudinal studies. Atypical classification was uncommon in this cohort, and larger studies enriched for nonamnestic AD presentations are warranted to better characterize this category. Future work should also target better characterization of low-burden classification for ^18^F-PI-2620. Finally, the cohort was predominantly composed of highly educated White non-Hispanic individuals recruited from academic centers, which limits generalizability to more racially and ethnically diverse and community-based populations. Nevertheless, this study represents the largest head-to-head tau PET dataset to date, enabling direct comparison and harmonization across tracers within a single framework.

## Conclusions

Per the results of this cross-sectional study, the harmonized visual reading framework for tau PET demonstrated high interrater and intertracer agreement across 4 radiotracers. Compared with the regulatory-approved framework, it detected significantly more tau-positive individuals at early disease stages. Five-class tau burden classification showed stepwise associations with cognitive performance, Aβ load, and plasma p-tau217. By enabling stratification beyond binary positivity, this harmonized tracer-agnostic framework may facilitate multicenter studies, support biomarker-informed treatment decisions and participant selection for clinical trials, and advance future clinical implementation of tau PET.

## Notes

Supplement 1. eMethods 1. Imaging Acquisition and Processing eMethods 2. Blood Sample Measurements eMethods 3. Harmonized Tau PET Visual Reading eTable 1. Amyloid PET Radiotracers and Scanners per Study Site eTable 2. Tau PET Radiotracers and Scanners per Study Site eTable 3. Demographics and Key Characteristics From HEAD Study Participants With All Four Tau PET Radiotracers eTable 4. Regional Tau PET Abnormality Prevalence in the Four-Tracer Subsample (n=131) and Two-Tracer Subsample (n=644) eTable 5. Demographics and Key Characteristics From HEAD Study Participants With [^1^^8^F]flortaucipir Tau PET eTable 6. Demographics and Key Characteristics From HEAD Study Participants With [^1^^8^F]RO948 Tau PET eTable 7. Demographics and Key Characteristics From HEAD Study Participants With [^1^^8^F]PI-2620 Tau PET eTable 8. Demographics and Key Characteristics From HEAD Study per Study Site eTable 9. Binary and Five-Level Classification Inter-Rater Agreement Across Subgroups eTable 10. PI-2620 Inter-Rater Agreement With and Without Composite 1 eTable 11. PI-2620 Inter-Tracer Agreement With and Without Composite 1 eTable 12. Binary and Five-Level Classification Inter-Tracer Agreement Across Subgroups eTable 13. Five-Level Classification Inter-Tracer Agreement by Scan Interval eTable 14. PI-2620 Classification With and Without Composite 1 eTable 15. Demographics of Individuals Classified as Atypical Tau Burden Using the Harmonized Framework eTable 16. Binary Tau PET Positivity Across Clinical Diagnoses eTable 17. Reclassification From Regulatory-Approved to Harmonized Framework eTable 18. Cognitive Composites (UDS-3) Across Harmonized [^1^^8^F]MK-6240 Classifications eTable 19. Cognitive Composites (UDS-3) Across Harmonized [^1^^8^F]flortaucipir Classifications eFigure 1. Study Sample Flowchart eFigure 2. Regional Abnormality Frequencies Across Tau PET Radiotracers Informing Composite Definitions eFigure 3. Regional Abnormality Prevalence at 1.5, 2.0, and 2.5 SD Thresholds Across Radiotracers eFigure 4. Examples of Regions With Frequent Off-Target Binding Excluded From the Harmonized Visual Reading Framework eFigure 5. Composite-Specific Visual Reference Masks eFigure 6. Harmonized Tau PET Visual Reading Tool eFigure 7. Mean Tau PET ([^1^^8^F]MK-6240) SUVR Images by Harmonized Five-Level Classification eFigure 8. Mean Tau PET ([^1^^8^F]flortaucipir) SUVR Images by Harmonized Five-Level Classification eFigure 9. Mean Tau PET ([^1^^8^F]RO948) SUVR Images by Harmonized Five-Level Classification eFigure 10. Mean Tau PET ([^1^^8^F]PI-2620) SUVR Images by Harmonized Five-Level Classification eFigure 11. Mean Tau PET SUVR Images by Harmonized Five-Level Classification Across the Four Radiotracers eFigure 12. Inter-rater Cohen's Kappa for Tau PET Regional Composites, Five-Level Classification, and Binary Positivity eFigure 13. Rater Confidence in Visual Reads eFigure 14. Patterns of Inter-Tracer Disagreements eFigure 15. Discordant Cases Where Harmonized [^1^^8^F]MK-6240 Was Positive and Harmonized [^1^^8^F]flortaucipir Was Negative eFigure 16. Discordant Cases Where Harmonized [^1^^8^F]MK-6240 Was Negative and Harmonized [^1^^8^F]flortaucipir Was Positive eFigure 17. Atypical Tau Burden Examples With Corresponding Radiotracer Images in Individual Participants eFigure 18. Frequencies of 5-Level Tau Burden Classification Across the Four Tau PET Radiotracers eFigure 19. Frequencies of Clinical Diagnoses in Each of the 5-Level Tau Burden Classification Across the Four Tau PET Radiotracers eFigure 20. Cognitive Performance, Amyloid-β Load, and Plasma p-tau217 With Binary Tau-Positivity Across Radiotracers and Frameworks eFigure 21. Discordant Tau PET Positivity Between Harmonized and Regulatory-Approved Visual Reading Frameworks eReferences.

Supplement 2. HEAD study members

Supplement 3. Data Sharing Statement
