# Supplementary material for: A Harmonized Visual Reading Framework for Tau PET Staging in Alzheimer Disease
Source: JAMA Neurol. 2026 Sep 21:e263253. Online ahead of print. doi: 10.1001/jamaneurol.2026.3253 (PMC13595235; doi:10.1001/jamaneurol.2026.3253)
Supplement: Supplementary file 1 [file jamaneurol-e263253-s001.pdf]

Supplemental Online Content

Ruppert E, Vermeiren MR, Scop Medeiros M, et al; HEAD study group. A harmonized visual reading framework for tau PET staging in Alzheimer disease. *JAMA Neurol*. Published online September 21, 2026. doi:10.1001/jamaneurol.2026.3253

eMethods 1. Imaging Acquisition and Processing
eMethods 2. Blood Sample Measurements
eMethods 3. Harmonized Tau PET Visual Reading
eTable 1. Amyloid PET Radiotracers and Scanners per Study Site
eTable 2. Tau PET Radiotracers and Scanners per Study Site
eTable 3. Demographics and Key Characteristics From HEAD Study Participants With All Four Tau PET Radiotracers
eTable 4. Regional Tau PET Abnormality Prevalence in the Four-Tracer Subsample (n=131) and Two-Tracer Subsample (n=644)
eTable 5. Demographics and Key Characteristics From HEAD Study Participants With [^{18}F]flortaucipir Tau PET
eTable 6. Demographics and Key Characteristics From HEAD Study Participants With [^{18}F]RO948 Tau PET
eTable 7. Demographics and Key Characteristics From HEAD Study Participants With [^{18}F]PI-2620 Tau PET
eTable 8. Demographics and Key Characteristics From HEAD Study per Study Site
eTable 9. Binary and Five-Level Classification Inter-Rater Agreement Across Subgroups
eTable 10. PI-2620 Inter-Rater Agreement With and Without Composite 1
eTable 11. PI-2620 Inter-Tracer Agreement With and Without Composite 1
eTable 12. Binary and Five-Level Classification Inter-Tracer Agreement Across Subgroups
eTable 13. Five-Level Classification Inter-Tracer Agreement by Scan Interval
eTable 14. PI-2620 Classification With and Without Composite 1
eTable 15. Demographics of Individuals Classified as Atypical Tau Burden Using the Harmonized Framework
eTable 16. Binary Tau PET Positivity Across Clinical Diagnoses
eTable 17. Reclassification From Regulatory-Approved to Harmonized Framework
eTable 18. Cognitive Composites (UDS-3) Across Harmonized [^{18}F]MK-6240 Classifications
eTable 19. Cognitive Composites (UDS-3) Across Harmonized [^{18}F]flortaucipir Classifications
eFigure 1. Study Sample Flowchart
eFigure 2. Regional Abnormality Frequencies Across Tau PET Radiotracers Informing Composite Definitions

© 2026 Ruppert E et al; HEAD study group. *JAMA Neurology*.

eFigure 3. Regional Abnormality Prevalence at 1.5, 2.0, and 2.5 SD Thresholds Across Radiotracers
eFigure 4. Examples of Regions With Frequent Off-Target Binding Excluded From the Harmonized Visual Reading Framework
eFigure 5. Composite-Specific Visual Reference Masks
eFigure 6. Harmonized Tau PET Visual Reading Tool
eFigure 7. Mean Tau PET ([^{18}F]MK-6240) SUVR Images by Harmonized Five-Level Classification
eFigure 8. Mean Tau PET ([^{18}F]flortaucipir) SUVR Images by Harmonized Five-Level Classification
eFigure 9. Mean Tau PET ([^{18}F]RO948) SUVR Images by Harmonized Five-Level Classification
eFigure 10. Mean Tau PET ([^{18}F]PI-2620) SUVR Images by Harmonized Five-Level Classification
eFigure 11. Mean Tau PET SUVR Images by Harmonized Five-Level Classification Across the Four Radiotracers
eFigure 12. Inter-rater Cohen's Kappa for Tau PET Regional Composites, Five-Level Classification, and Binary Positivity
eFigure 13. Rater Confidence in Visual Reads
eFigure 14. Patterns of Inter-Tracer Disagreements
eFigure 15. Discordant Cases Where Harmonized [^{18}F]MK-6240 Was Positive and Harmonized [^{18}F]flortaucipir Was Negative
eFigure 16. Discordant Cases Where Harmonized [^{18}F]MK-6240 Was Negative and Harmonized [^{18}F]flortaucipir Was Positive
eFigure 17. Atypical Tau Burden Examples With Corresponding Radiotracer Images in Individual Participants
eFigure 18. Frequencies of 5-Level Tau Burden Classification Across the Four Tau PET Radiotracers
eFigure 19. Frequencies of Clinical Diagnoses in Each of the 5-Level Tau Burden Classification Across the Four Tau PET Radiotracers
eFigure 20. Cognitive Performance, Amyloid-β Load, and Plasma p-tau217 With Binary Tau-Positivity Across Radiotracers and Frameworks
eFigure 21. Discordant Tau PET Positivity Between Harmonized and Regulatory-Approved Visual Reading Frameworks
eReferences.

This supplemental material has been provided by the authors to give readers additional information about their work.

© 2026 Ruppert E et al; HEAD study group. *JAMA Neurology*.

eMethods 1. Imaging Acquisition and Processing

eMethods 1. Imaging acquisition and processing.

MRI scans were acquired on 3T scanners using harmonized protocols aligned with ADNI4 guidelines.[1] High-resolution 3D T1-weighted images were obtained for all participants and used for PET co-registration and spatial normalization. T1-weighted images were processed using Advanced Normalization Tools (ANTs) to generate subject-specific transformations to stereotaxic space. PET data were reconstructed into 5-minute frames using scanner-specific ordered-subset expectation maximization (OSEM) algorithms with standardized reconstruction settings. All PET images underwent corrections for attenuation, motion, dead time, decay, scatter, and random events, and final PET volumes were smoothed with scanner-specific Gaussian kernels to achieve a uniform spatial resolution of 8-mm full width at half maximum (FWHM). PET images were co-registered to native-space T1-weighted MRI using linear transformations implemented in ANTs. MRI-derived spatial transformations were subsequently applied to bring PET images into stereotaxic space, and individual whole-brain masks were applied to exclude non-brain voxels.

© 2026 Ruppert E et al; HEAD study group. *JAMA Neurology*.

eMethods 2. Blood Sample Measurements

Blood samples were collected in commercial ethylenediaminetetraacetic (EDTA) tubes per NCRAD guidelines, aliquoted, stored at −80 °C, and shipped under temperature-controlled conditions to the University of Pittsburgh for first-thaw processing. Plasma p-tau217 was measured using the ALZpath Advantage PLUS assay (#104570) on the single-molecule array (Simoa) platform (Quanterix®).

© 2026 Ruppert E et al; HEAD study group. *JAMA Neurology*.

eMethods 3. Harmonized Tau PET Visual Reading

2. 1. General considerations

The harmonized tau PET visual reading tool, available at uni-tau.com, is a web-based platform designed to support standardized, clinician-friendly visual reads of tau PET scans while minimizing interpretative variability. It enables harmonized assessment of neurofibrillary tangle (NFT) abnormalities across tau PET radiotracers ([^{18}F]flortaucipir, [^{18}F]MK-6240, [^{18}F]PI-2620, [^{18}F]RO948).

To assist in evaluating NFT distribution within the cerebral cortex, the harmonized visual reading method incorporates visual reference masks. These anatomical guides help raters delineate boundaries but should be used only as a reference, they do not independently determine positivity.

The tool guides the reader through seven composites spanning early- to late-stage tau deposition. Each composite is rated as positive or negative. Based on regional ratings, individuals are classified into one of five patterns:

- Tau PET negative
- Tau PET positive consistent with Alzheimer's disease (AD) pattern, this is then subclassified to three different stages:
    - low tau burden
    - moderate tau burden
    - high tau burden
- Tau PET positive in an atypical pattern for AD pathology.

Future iterations of this framework may consolidate the seven composites into different regional groupings to further enhance clinical utility and scalability. This conceptual extension is considered part of the ongoing development of the harmonized visual reading method.

2. 2. Image Upload and Preprocessing

Brain imaging data (tau PET and structural MRI) can be uploaded in:

- Preprocessed format: SUVR and MRI images already normalized to the ICBM/MNI 152 template in NIfTI (.nii) format can be directly uploaded.

After image upload, default visualization settings are applied (e.g., SUVR thresholds 0.3–2.3; "*nipy_spectral"* colormap).

2. 3. Positivity criteria for composite evaluation

A region is considered positive only if there is a clearly visible peak of tracer uptake within the grey matter that matches the expected cortical anatomy. This uptake must be visible in all three anatomical planes (e.g. sagittal, axial, and coronal) and present in at least two consecutive slices. Composites do not need to be positive in all the composing regions to be considered positive.

Composite-specific criteria were structured to address two different objectives using a single method:

- Early Detection - Composites 1, 2: Composites 1 and 2 are intended to capture early tau tangle deposition. These composites should be rated with an emphasis on increased sensitivity. Any clearly visible focus of abnormal uptake that meets the main criteria should be interpreted as positive.
- Staging - Composites 3, 4, 5, 6, 7: Composites 3 through 7 are intended for staging tau accumulation. These regions should be rated as positive only when a distinct high-density peak (e.g., red signal using the tool's default parameters) is present and meets the main criteria. Small, non-contiguous foci or diffuse yellow uptake without a clear red peak should be interpreted as negative. Spillover from adjacent regions, off-target binding, or signal peaking outside the composite but extending into its periphery should also be classified as negative. In cases of uncertainty, a negative rating is preferred, as the individual is unlikely to exhibit tau levels indicative of a more advanced disease stage. This conservative approach is based on the principle that individuals with low-density tau accumulation in a region are more clinically aligned with those showing clear pathology in earlier (anterior) regions, rather than with those exhibiting high-density tau in the region under evaluation.

Following completion of all seven composite assessments, the tool automatically assigns one of the five-level tau PET categories described above.

© 2026 Ruppert E et al; HEAD study group. *JAMA Neurology*.

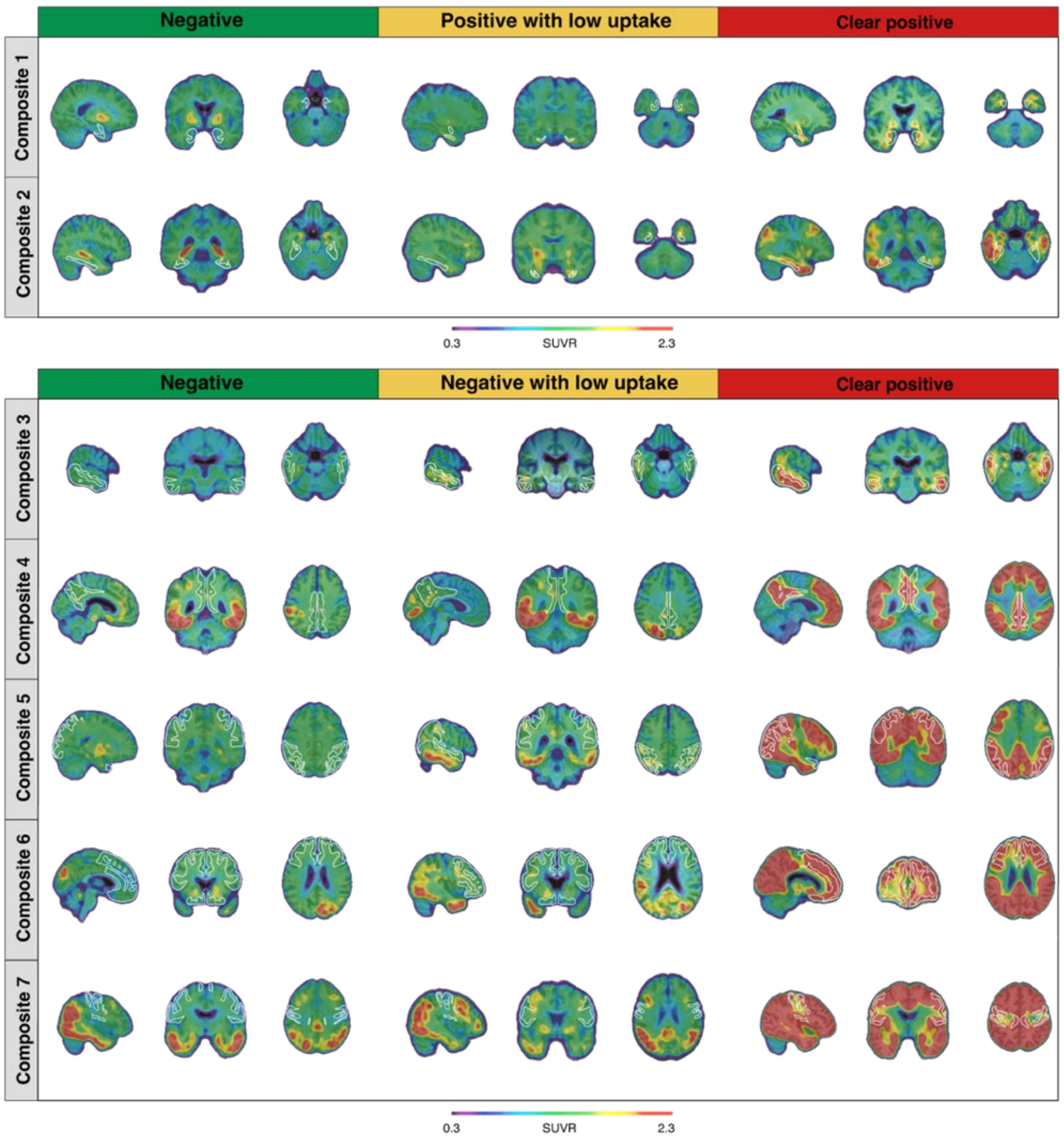

Figure 1. Examples of [^{18}F]flortaucipir scans illustrating varying levels of uptake in each of the seven composites. For composites 1 and 2, early detection regions, we recommend a more sensitive interpretation, where clearly visible focus of abnormal uptake should be considered positive, to ensure early tau tangle detection. The following five other composites are meant for staging of the extent of tau pathology in the brain and, to avoid false positives, should only be considered positive if there is a clear peak of uptake (in red) within the region itself.

© 2026 Ruppert E et al; HEAD study group. *JAMA Neurology*.

eTable 1. Amyloid PET Radiotracers and Scanners per Study Site

Site	Amyloid PET tracer	Amyloid PET scanner	MRI scanner
Brown University	[^{18}F] Florbetaben	Siemens Biograph mCT 4R PET/CT	Siemens Prisma Fit
Houston Methodist Research Institute	[^{11}C] PiB	Siemens Biograph Vision 600 Edge PET/CT<br>Siemens Biograph Vision Quadra Edge PET/CT	Siemens Vida
Lawrence Berkeley National Laboratory	[^{11}C] PiB	Siemens Biograph mCT TruePoint PET/CT	Siemens Prisma Fit<br>Siemens TrioTim
Mayo Clinic	[^{11}C] PiB	GE Discovery 690 PET/CT<br>GE Discovery MI PET/CT<br>Siemens Biograph Vision 600 PET/CT	Siemens Prisma
McGill University	[^{18}F] NAV4694	GE Discovery 690 PET/CT<br>Siemens ECAT HR+ (HRRT) PET	Siemens Prisma Fit
Sant Pau Biomedical Research Institute	[^{18}F] Florbetaben<br>[^{18}F] Flutemetamol	Philips Vereos PET/CT	Philips Ingenia Elition X
University of California San Francisco	[^{18}F] Florbetaben	Siemens Biograph Vision 600 PET/CT	Siemens Prisma Fit
University of Pittsburgh	[^{11}C] PiB	Siemens Biograph mCT PET/CT<br>Siemens ECAT HR PET	Siemens Prisma Fit
Washington University in St. Louis	[^{11}C] PiB	Siemens Biograph mCT TruePoint PET/CT<br>Siemens Biograph Vision 600 Edge PET/CT	Siemens Prisma Fit

Acquisition windows for Amyloid PET tracers (minutes post-injection): [^{11}C]PiB, 40-60 or 50-70; [^{18}F]NAV4694, 40-70; [^{18}F]Florbetaben, 90-110; [^{18}F]Flutemetamol, 90-110. Acquisition windows were standardized per radiotracer across all sites.

© 2026 Ruppert E et al; HEAD study group. *JAMA Neurology*.

eTable 2. Tau PET Radiotracers and Scanners per Study Site

Site	Tau PET tracer	Tau PET scanner	MRI scanner
Brown University	[^{18}F]flortaucipir<br>[^{18}F]MK-6240	Siemens Biograph mCT 4R PET/CT	Siemens Prisma Fit
Houston Methodist Research Institute	[^{18}F]flortaucipir<br>[^{18}F]MK-6240	Siemens Biograph Vision 600 Edge PET/CT<br>Siemens Biograph Vision Quadra Edge PET/CT	Siemens Vida
Lawrence Berkeley National Laboratory	[^{18}F]flortaucipir<br>[^{18}F]MK-6240	Siemens Biograph mCT TruePoint PET/CT	Siemens Prisma Fit<br>Siemens TrioTim
Mayo Clinic	[^{18}F]flortaucipir<br>[^{18}F]MK-6240	GE Discovery 690 PET/CT<br>GE Discovery MI PET/CT<br>Siemens Biograph Vision 600 PET/CT	Siemens Prisma
McGill University	[^{18}F]flortaucipir<br>[^{18}F]MK-6240<br>[^{18}F]PI-2620<br>[^{18}F]RO948	GE Discovery 690 PET/CT<br>Siemens ECAT HR+ (HRRT) PET	Siemens Prisma Fit
Sant Pau Biomedical Research Institute	[^{18}F]MK-6240<br>[^{18}F]PI-2620<br>[^{18}F]RO948	Philips Vereos PET/CT	Philips Ingenia Elition X
University of California San Francisco	[^{18}F]flortaucipir<br>[^{18}F]MK-6240	Siemens Biograph Vision 600 PET/CT	Siemens Prisma Fit
University of Pittsburgh	[^{18}F]flortaucipir<br>[^{18}F]MK-6240	Siemens Biograph mCT PET/CT	Siemens Prisma Fit
Washington University in St. Louis	[^{18}F]flortaucipir<br>[^{18}F]MK-6240	Siemens Biograph mCT TruePoint PET/CT<br>Siemens Biograph Vision 600 Edge PET/CT	Siemens Prisma Fit

Acquisition windows for Tau PET tracers (minutes post-injection): [^{18}F]flortaucipir, 80-100; [^{18}F]MK-6240, 90-110; [^{18}F]PI-2620, 45-75; [^{18}F]RO948, 70-90. Acquisition windows were standardized per radiotracer across all sites.

© 2026 Ruppert E et al; HEAD study group. *JAMA Neurology*.

eTable 3. Demographics and Key Characteristics From HEAD Study Participants With All Four Tau PET Radiotracers

	**CU** (n=68)	**MCI** (n=50)	**Dementia** (n=13)
**Age,** mean (SD), y	65.0 (8.0)	69.7 (7.4)	64.8 (7.6)
**Sex,** No. (%)			
Female	46 (67.6)	27 (54.0)	5 (38.5)
Male	22 (32.4)	23 (46.0)	8 (61.5)
**Education,** mean (SD), y	15.3 (2.9)	15.3 (3.5)	15.8 (2.9)
**Race,** No. (%)			
Asian	0 (0.0)	0 (0.0)	0 (0.0)
Black or African American	0 (0.0)	1 (2.0)	0 (0.0)
White	67 (98.5)	49 (98.0)	13 (100.0)
Other/Unknown	1 (1.5)	0 (0.0)	0 (0.0)
**Ethnicity,** No. (%)			
Hispanic/Latino	1 (1.5)	0 (0.0)	0 (0.0)
Non-hispanic/Latino	67 (98.5)	50 (100.0)	13 (100.0)
**APOE ε4,** No. (%)			
Carrier	23 (33.8)	25 (50.0)	9 (69.2)
Non-Carrier	45 (66.2)	25 (50.0)	4 (30.8)
**MMSE score,** mean (SD)	28.9 (1.2)	27.3 (2.6)	24.5 (4.0)
**MoCA score,** mean (SD)	27.0 (2.2)	24.3 (3.8)	18.3 (6.1)
**Aβ PET visual read,** No. (%)			
Negative	51 (75.0)	20 (40.0)	1 (7.7)
Positive	17 (25.0)	30 (60.0)	12 (92.3)
**Centiloids**, mean (SD)	13.5 (28.4)	61.3 (57.6)	119.1 (50.7)
**Plasma p-tau217,** mean (SD), pg/mL	0.3 (0.1)	0.7 (0.4)	1.1 (0.7)

Abbreviations: Aβ, amyloid-β; APOE ε4, apolipoprotein ε4 allele; CU, cognitively unimpaired; MCI, mild cognitive impairment; MMSE, Mini Mental State Examination; MoCA, Montreal Cognitive Assessment. Missing values: plasma p-tau217 (n = 1).

© 2026 Ruppert E et al; HEAD study group. *JAMA Neurology*.

eTable 4. Regional Tau PET Abnormality Prevalence in the Four-Tracer Subsample (n=131) and Two-Tracer Subsample (n=644)

	**MK-6240**[1] (n = 131)	**Flortaucipir**[1] (n = 131)	**MK-6240**[2] (n = 644)	**Flortaucipir**[2] (n = 644)
**Amygdala**	28.27	20.79	28.77	22.04
**Entorhinal**	23.39	18.97	23.87	19.09
**Fusiform**	22.32	18.32	22.61	18.09
**Inferior Temporal**	19.13	16.99	20.93	17.42
**Middle Temporal**	18.93	16.96	19.70	17.13
**Posterior Cingulate**	18.27	15.49	17.72	14.23
**Precuneus**	18.08	15.18	17.16	14.05
**Inferior Parietal**	16.46	14.77	15.89	12.14
**Superior Temporal**	15.93	12.23	15.70	13.37
**Lateral Occipital**	14.44	11.33	14.82	12.23
**Supramarginal**	13.63	11.83	13.38	10.62
**Superior Parietal**	13.52	11.12	12.96	10.26
**Anterior Cingulate**	14.89	9.15	12.96	8.29
**Middle Frontal**	13.16	11.34	11.20	9.47
**Inferior Frontal**	11.98	10.84	10.84	8.89
**Superior Frontal**	11.84	9.34	10.5	8.09
**Orbitofrontal**	9.81	7.79	9.89	6.83
**Paracentral**	11.35	7.43	9.49	7.28
**Precentral**	10.67	8.19	9.14	6.93
**Postcentral**	9.84	8.02	8.79	6.63

[1] Using n = 51 CU Aβ negatives
[2] Using n = 280 CU Aβ negatives

Percentage of participants with abnormal voxels (>2 SD above CU Aβ-negative reference mean) per region of interest for [^{18}F]MK-6240 and [^{18}F]flortaucipir. The four-tracer subsample used CU Aβ-negative participants from the four-tracer cohort as the reference group (n=51), while the two-tracer subsample used CU Aβ-negative participants from the [^{18}F]MK-6240/[^{18}F]flortaucipir cohort (n=280). Regions are ordered by descending abnormality prevalence. Regional ordering and composite assignments remained consistent across reference groups.

© 2026 Ruppert E et al; HEAD study group. *JAMA Neurology*.

eTable 5. Demographics and Key Characteristics From HEAD Study Participants With [^{18}F]flortaucipir Tau PET

	**CU** (n=364)	**MCI** (n=223)	**Dementia** (n=57)
**Age,** mean (SD), y	67.1 (8.3)	71.5 (7.7)	70.9 (8.8)
**Sex,** No. (%)			
Female	221 (60.7)	104 (46.6)	25 (43.9)
Male	143 (39.3)	119 (53.4)	32 (56.1)
**Education,** mean (SD), y	16.1 (2.5)	15.8 (3.1)	15.8 (2.8)
**Race,** No. (%)			
Asian	4 (1.1)	7 (3.1)	0 (0.0)
Black or African American	15 (4.1)	4 (1.8)	3 (5.3)
White	343 (94.2)	210 (94.2)	53 (93.0)
Other/Unknown	2 (0.5)	2 (0.9)	1 (1.8)
**Ethnicity,** No. (%)			
Hispanic/ latino	7 (1.9)	13 (5.8)	5 (8.8)
Non-hispanic/ latino	357 (98.1)	210 (94.2)	52 (91.2)
**APOE ε4**, No. (%)			
Carrier	110 (30.6)	112 (50.2)	40 (71.4)
Non-Carrier	250 (69.4)	111 (49.8)	16 (28.6)
**MMSE score,** mean (SD)	28.7 (1.5)	26.4 (3.1)	20.7 (6.3)
**MoCA score**, mean (SD)	27.1 (2.2)	22.9 (4.1)	15.6 (6.7)
**Aβ PET visual read,** No. (%)			
Negative	280 (76.9)	79 (35.4)	6 (10.5)
Positive	84 (23.1)	144 (64.6)	51 (89.5)
**Centiloids,** mean (SD)	14.2 (32.4)	61.2 (56.0)	102.8 (50.7)
**Plasma p-tau217**, mean (SD), pg/mL	0.4 (0.3)	0.8 (0.6)	1.4 (0.8)

Abbreviations: Aβ, amyloid-β; APOE ε4, apolipoprotein ε4 allele; CU, cognitively unimpaired; MCI, mild cognitive impairment; MMSE, Mini Mental State Examination; MoCA, Montreal Cognitive Assessment. Missing values: Education (n = 4), APOE ε4 (n = 5), MoCA score (n = 3), plasma p-tau217 (n = 11).

© 2026 Ruppert E et al; HEAD study group. *JAMA Neurology*.

eTable 6. Demographics and Key Characteristics From HEAD Study Participants With [^{18}F]RO948 Tau PET

	**CU** (n=71)	**MCI** (n=60)	**Dementia** (n=19)
**Age,** mean (SD), y	65.3 (8.0)	70.3 (7.2)	68.9 (8.9)
**Sex,** No. (%)			
Female	48 (67.6)	30 (50.0)	10 (52.6)
Male	23 (32.4)	30 (50.0)	9 (47.4)
**Education,** mean (SD), y	15.2 (3.0)	15.0 (3.7)	14.9 (3.0)
**Race,** No. (%)			
Asian	0 (0.0)	0 (0.0)	0 (0.0)
Black or African American	0 (0.0)	1 (1.7)	0 (0.0)
White	70 (98.6)	59 (98.3)	19 (100.0)
Other/Unknown	1 (1.4)	0 (0.0)	0 (0.0)
**Ethnicity,** No. (%)			
Hispanic/ latino	1 (1.4)	0 (0.0)	0 (0.0)
Non-hispanic/ latino	70 (98.6)	60 (100.0)	19 (100.0)
**APOE ε4**, No. (%)			
Carrier	26 (36.6)	29 (48.3)	10 (52.6)
Non-Carrier	45 (63.4)	31 (51.7)	9 (47.4)
**MMSE score**, mean (SD)	28.9 (1.2)	27.2 (2.6)	23.5 (4.1)
**MoCA score**, mean (SD)	27.0 (2.2)	24.0 (3.9)	17.7 (5.6)
**Aβ PET visual read,** No. (%)			
Negative	52 (73.2)	27 (45.0)	4 (21.1)
Positive	19 (26.8)	33 (55.0)	15 (78.9)
**Centiloids,** mean (SD)	14.7 (29.0)	53.7 (56.0)	89.1 (64.3)
**Plasma p-tau217,** mean (SD), pg/mL	0.3 (0.2)	0.7 (0.4)	1.1 (0.9)

Abbreviations: Aβ, amyloid-β; APOE ε4, apolipoprotein ε4 allele; CU, cognitively unimpaired; MCI, mild cognitive impairment; MMSE, Mini Mental State Examination; MoCA, Montreal Cognitive Assessment. Missing values: MMSE score (n = 1), plasma p-tau217 (n= 2).

© 2026 Ruppert E et al; HEAD study group. *JAMA Neurology*.

eTable 7. Demographics and Key Characteristics From HEAD Study Participants With [^{18}F]PI-2620 Tau PET

	**CU** (n=71)	**MCI** (n=61)	**Dementia** (n=17)
**Age,** mean (SD), y	65.2 (7.9)	71.0 (7.9)	68.3 (9.5)
**Sex,** No. (%)			
Female	48 (67.6)	31 (50.8)	6 (35.3)
Male	23 (32.4)	30 (49.2)	11 (64.7)
**Education,** mean (SD), y	15.4 (2.9)	14.7 (4.0)	15.4 (2.8)
**Race,** No. (%)			
Asian	0 (0.0)	0 (0.0)	0 (0.0)
Black or African American	0 (0.0)	2 (3.3)	0 (0.0)
White	70 (98.6)	59 (96.7)	17 (100.0)
Other/Unknown	1 (1.4)	0 (0.0)	0 (0.0)
**Ethnicity,** No. (%)			
Hispanic/ latino	1 (1.4)	0 (0.0)	1 (5.9)
Non-hispanic/ latino	70 (98.6)	61 (100.0)	16 (94.1)
**APOE ε4,** No. (%)			
Carrier	25 (35.2)	31 (50.8)	10 (58.8)
Non-Carrier	46 (64.8)	30 (49.2)	7 (41.2)
**MMSE score,** mean (SD)	28.9 (1.2)	27.0 (2.6)	24.2 (4.4)
**MoCA score,** mean (SD)	27.0 (2.2)	23.5 (4.2)	17.9 (6.2)
**Aβ PET visual read,** No. (%)			
Negative	54 (76.1)	23 (37.7)	3 (17.6)
Positive	17 (23.9)	38 (62.3)	14 (82.4)
**Centiloids**, mean (SD)	13.0 (27.9)	55.0 (54.6)	94.9 (63.5)
**Plasma p-tau217,** pg/mL, mean (SD)	0.3 (0.1)	0.7 (0.5)	1.2 (0.6)

Abbreviations: Aβ, amyloid-β; APOE ε4, apolipoprotein ε4 allele; CU, cognitively unimpaired; MCI, mild cognitive impairment; MMSE, Mini Mental State Examination; MoCA, Montreal Cognitive Assessment. Missing values: MMSE score (n = 1), plasma p-tau217 (n = 2).

© 2026 Ruppert E et al; HEAD study group. *JAMA Neurology*.

eTable 8. Demographics and Key Characteristics From HEAD Study per Study Site

	**Brown University** (n=36)	**Houston Methodist Research Institute** (n=104)	**Lawrence Berkeley National Laboratory** (n=22)	**Mayo Clinic** (n=40)	**McGill University** (n=203)	**Sant Pau Biomedical Research Institute** (n=37)	**University of California San Francisco** (n=14)	**University of Pittsburgh** (n=127)	**Washington University in St. Louis** (n=98)
**Age,** mean (SD), y	67.8 (6.4)	71.3 (8.4)	75.4 (6.1)	74.0 (7.5)	67.5 (7.8)	75.4 (6.1)	69.8 (6.2)	65.9 (8.6)	70.4 (8.6)
**Sex,** No. (%)									
Female	18 (50.0)	46 (44.2)	10 (45.5)	16 (40.0)	124 (61.6)	17 (45.9)	5 (35.7)	72 (56.7)	59 (60.2)
Male	18 (50.0)	58 (55.8)	12 (54.5)	24 (60.0)	79 (38.9)	20 (54.1)	9 (64.3)	55 (43.3)	39 (39.8)
**Education**, mean (SD), y	16.7 (2.1)	16.4 (2.4)	17.3 (2.1)	15.2 (1.9)	15.6 (3.2)	13.2 (4.1)	16.9 (2.2)	16.0 (2.7)	15.6 (2.5)
**APOE ε4**, No. (%)									
Carrier	13 (36.1)	61 (58.7)	8 (36.4)	6 (15.0)	89 (44.1)	17 (45.9)	8 (61.5)	40 (32.3)	37 (37.8)
Non-Carrier	23 (63.9)	43 (41.3)	14 (63.6)	34 (85.0)	113 (55.9)	20 (54.1)	5 (38.5)	84 (67.7)	61 (62.2)
**Clinical diagnosis,** No. (%)									
CU	34 (94.4)	21 (20.2)	16 (72.7)	35 (87.5)	106 (52.5)	5 (13.5)	0 (0.0)	91 (71.7)	59 (60.2)
MCI	2 (5.6)	67 (64.4)	3 (13.6)	5 (12.5)	78 (38.4)	22 (59.5)	9 (64.3)	28 (22.0)	33 (33.7)
Dementia	0 (0.0)	16 (15.4)	3 (13.6)	0 (0.0)	19 (9.4)	10 (27.0)	5 (35.7)	8 (6.3)	6 (6.1)
**Aβ PET visual read,** No. (%)									
Negative	31 (86.1)	34 (32.7)	10 (45.5)	28 (70.0)	105 (51.7)	19 (51.4)	6 (42.9)	93 (73.2)	58 (59.2)
Positive	5 (13.9)	70 (67.3)	12 (54.5)	12 (30.0)	98 (48.3)	18 (48.6)	8 (57.1)	34 (26.8)	40 (40.8)

Abbreviations: Aβ, amyloid-β; APOE ε4, apolipoprotein ε4 allele; CU, cognitively unimpaired; MCI, mild cognitive impairment. Missing values: Education (n = 4), APOE ε4 (n = 5).

© 2026 Ruppert E et al; HEAD study group. *JAMA Neurology*.

eTable 9. Binary and Five-Level Classification Inter-Rater Agreement Across Subgroups

**Binary positivity agreement,** No. (%) – Fleiss κ				
	**MK-6240**	**Flortaucipir**	**RO948**	**PI-2620**
All participants	619 (90.9) – 0.88	603 (93.6) – 0.91	127 (84.7) – 0.78	144 (96.6) – 0.93
Aβ-positive participants	282 (94.9) – 0.91	266 (95.3) – 0.93	59 (88.1) – 0.83	66 (95.7) – 0.94
CI participants	295 (94.6) – 0.92	265 (94.6) – 0.92	64 (81.0) – 0.73	76 (96.2) – 0.95
Four-tracer subsample	118 (90.1) – 0.86	122 (93.1) – 0.90	111 (84.7) – 0.78	127 (96.9) – 0.94
Excluding four-tracer subsample	501 (91.1) – 0.88	481 (93.8) – 0.91	16 (84.2) – 0.79	17 (94.4) – 0.88
**5-level classification agreement,** No. (%) – Fleiss κ				
	**MK-6240**	**Flortaucipir**	**RO948**	**PI-2620**
All participants	568 (83.4) – 0.82	552 (85.7) – 0.81	122 (81.3) – 0.77	137 (91.9) – 0.85
Aβ-positive participants	239 (80.5) – 0.82	220 (78.9) – 0.80	55 (82.1) – 0.84	59 (85.5) – 0.84
CI participants	257 (82.4) – 0.84	227 (81.1) – 0.82	59 (74.7) – 0.77	69 (87.3) – 0.85
Four-tracer subsample	109 (83.2) – 0.80	114 (87.0) – 0.83	106 (80.9) – 0.76	121 (92.4) – 0.86
Excluding four-tracer subsample	459 (83.5) – 0.82	438 (85.4) – 0.81	16 (84.2) – 0.84	16 (88.9) – 0.77

Abbreviations: Aβ, amyloid-β; CI, cognitively impaired; MK-6240, [^{18}F]MK-6240; Flortaucipir, [^{18}F]flortaucipir; RO948, [^{18}F]RO948; PI-2620, [^{18}F]PI-2620.
Sample sizes: All participants: MK-6240 (n=681), Flortaucipir (n=644), RO948 (n=150), PI-2620 (n=149); Aβ-positive participants: MK-6240 (n=297), Flortaucipir (n=279), RO948 (n=67), PI-2620 (n=69); CI participants: MK-6240 (n=312), Flortaucipir (n=280), RO948 (n=79), PI-2620 (n=79); Four-tracer subsample: n=131; Excluding four-tracer subsample: MK-6240 (n=550), Flortaucipir (n=513), RO948 (n=19), PI-2620 (n=18).
Agreements among three independent blinded raters.

© 2026 Ruppert E et al; HEAD study group. *JAMA Neurology*.

eTable 10. PI-2620 Inter-Rater Agreement With and Without Composite 1

	**PI-2620 without C1**	**PI-2620 with C1**
**Binary positivity agreement,** No. (%) – Fleiss κ		
All participants	144 (96.6) – 0.93	97 (65.1) – 0.51
Aβ-positive participants	66 (95.7) – 0.94	52 (75.4) – 0.66
CI participants	76 (96.2) – 0.95	56 (70.9) – 0.60
Four-tracer subsample	127 (96.9) – 0.94	84 (64.1) – 0.49
**5-level classification agreement,** No. (%) – Fleiss κ		
All participants	140 (91.9) – 0.85	92 (61.7) – 0.55
Aβ-positive participants	59 (85.5) – 0.84	47 (68.1) – 0.69
CI participants	69 (87.3) – 0.85	51 (64.6) – 0.66
Four-tracer subsample	121 (92.4) – 0.86	80 (61.1) – 0.53

Abbreviations: Aβ, amyloid-β; CI, cognitively impaired; C1 (composite 1); PI-2620, [^{18}F]PI-2620.
Sample sizes: all participants (n = 149); Aβ-positive (n = 69); CI participants (n = 79); 4-tracer subsample (n = 131).
Agreements among three independent blinded raters.

© 2026 Ruppert E et al; HEAD study group. *JAMA Neurology*.

eTable 11. PI-2620 Inter-Tracer Agreement With and Without Composite 1

**Binary inter-tracer agreements per tracer pair,** No. (%) – Cohen's κ			
**Participants**	**Tracer pair**	**PI-2620 without C1**	**PI-2620 with C1**
All participants	MK – PI	121 (81.2) – 0.57	124 (83.2) – 0.65
	FTP – PI	111 (84.7) – 0.63	107 (81.7) – 0.60
	RO – PI	114 (87.0) – 0.68	106 (80.9) – 0.58
Aβ-positive participants	MK – PI	53 (76.8) – 0.56	60 (87.0) – 0.72
	FTP – PI	50 (84.7) – 0.70	53 (89.8) – 0.80
	RO – PI	50 (84.7) – 0.70	51 (86.4) – 0.72
CI participants	MK – PI	61 (77.2) – 0.57	68 (86.1) – 0.71
	FTP – PI	53 (84.1) – 0.69	55 (87.3) – 0.74
	RO – PI	49 (77.8) – 0.57	51 (81.0) – 0.6
Four-tracer subsample	MK – PI	109 (83.2) – 0.61	109 (83.2) – 0.62
**5-level classification inter-tracer agreements per tracer pair,** No. (%) – Cohen's κ			
**Participants**	**Tracer pair**	**PI-2620 without C1**	**PI-2620 with C1**
All participants	MK – PI	112 (75.2) – 0.87	115 (77.2) – 0.88
	FTP – PI	104 (79.4) – 0.79	100 (76.3) – 0.79
	RO – PI	108 (82.4) – 0.82	102 (77.9) – 0.80
Aβ-positive participants	MK – PI	44 (63.8) – 0.86	51 (73.9) – 0.90
	FTP – PI	43 (72.9) – 0.86	46 (78.0) – 0.92
	RO – PI	44 (74.6) – 0.85	47 (79.7) – 0.86
CI participants	MK – PI	52 (65.8) – 0.89	60 (75.9) – 0.91
	FTP – PI	46 (73.0) – 0.84	49 (77.8) – 0.87
	RO – PI	43 (68.3) – 0.75	47 (74.6) – 0.75
Four-tracer subsample	MK – PI	101 (77.1) – 0.87	101 (77.1) – 0.87

Abbreviations: Aβ, amyloid-β; CI, cognitively impaired; C1, composite 1; MK, [^{18}F]MK-6240; FTP, [^{18}F]flortaucipir; RO, [^{18}F]RO948; PI, [^{18}F]PI-2620.

Sample sizes: All participants: MK-PI (n=149), FTP-PI (n=131), RO-PI (n=131). Aβ-positive participants: MK-PI (n=69), FTP-PI (n=59), RO-PI (n=59). CI participants: MK-PI (n=79), FTP-PI (n=63), RO-PI (n=63). For FTP-PI and RO-PI, the four-tracer subsample is equivalent to all participants (n=131).

Agreements using majority vote of the three raters.

© 2026 Ruppert E et al; HEAD study group. *JAMA Neurology*.

eTable 12. Binary and Five-Level Classification Inter-Tracer Agreement Across Subgroups

**Binary positivity agreement,** No. (%) – Unweighted Cohen's κ						
	**MK- FTP**	**MK - RO**	**MK - PI**	**FTP - RO**	**FTP - PI**	**RO - PI**
All participants	570 (88.5) – 0.76	131 (87.3) – 0.73	121 (81.2) - 0.57	112 (85.5) – 0.68	111 (84.7) - 0.63	114 (87.0) – 0.68
Aβ-positive participants	242 (86.7) – 0.68	59 (88.1) – 0.74	53 (76.8) – 0.56	51 (86.4) – 0.70	50 (84.7) – 0.71	50 (84.7) – 0.70
CI participants	246 (87.9) – 0.74	68 (86.1) – 0.70	61 (77.2) – 0.57	53 (84.1) – 0.66	53 (84.1) – 0.69	49 (77.8) – 0.57
Four-tracer subsample	115 (87.8) – 0.74	116 (88.5) – 0.75	109 (83.2) – 0. 61	112 (85.5) – 0.68	111 (84.7) – 0.63	114 (87.0) – 0.68
Excluding four-tracer subsample	455 (88.7) – 0.76	15 (78.9) – 0.59	12 (66.7) – 0.33	NA	NA	NA
**5-level classification agreement,** No. (%) – Weighted Cohen's κ						
	**MK- FTP**	**MK - RO**	**MK - PI**	**FTP - RO**	**FTP - PI**	**RO - PI**
All participants	529 (82.1) – 0.88	125 (83.3) – 0.83	112 (75.2) – 0.87	109 (83.2) – 0.73	104 (79.4) – 0.79	108 (82.4) – 0.82
Aβ-positive participants	206 (73.8) – 0.87	54 (80.6) – 0.83	44 (63.8) – 0.86	49 (83.1) – 0.84	43 (72.9) – 0.86	44 (74.6) – 0.85
CI participants	218 (77.9) – 0.88	63 (79.7) – 0.81	52 (65.8) – 0.89	50 (79.4) – 0.73	46 (73.0) – 0.84	43 (68.3) – 0.75
Four-tracer subsample	110 (84.0) – 0.84	111 (84.7) – 0.84	101 (77.1) – 0.87	109 (83.2) – 0.73	104 (79.4) – 0.79	108 (82.3) – 0.82
Excluding four-tracer subsample	419 (81.7) – 0.89	14 (73.7) – 0.76	11 (61.1) – 0.83	NA	NA	NA

Abbreviations: Aβ, amyloid-β; CI, cognitively impaired; MK, [^{18}F]MK-6240; FTP, [^{18}F]flortaucipir; RO, [^{18}F]RO948; PI, [^{18}F]PI-2620.
Sample sizes: All participants: MK vs FTP (n=644), MK vs RO (n=150), MK vs PI (n=149), FTP vs RO (n=131), FTP vs PI (n=131), RO vs PI (n=131); Aβ-positive participants: MK vs FTP (n=279), MK vs RO (n=67), MK vs PI (n=69), FTP vs RO (n=59), FTP vs PI (n=59), RO vs PI (n=59); CI participants: MK vs FTP (n=280), MK vs RO (n=79), MK vs PI (n=79), FTP vs RO (n=63), FTP vs PI (n=63), RO vs PI (n=63); Four-tracer subsample: n=131; Excluding four-tracer subsample: MK vs FTP (n=513), MK vs RO (n=19), MK vs PI (n=18).
Agreements using majority vote of the three raters.
In the excluding four-tracer subsample, small sample sizes and skewed marginal distributions deflated Cohen's κ for MK vs RO (n=19) and MK vs PI (n=18) despite moderate to high percent agreement (prevalence paradox).

© 2026 Ruppert E et al; HEAD study group. *JAMA Neurology*.

eTable 13. Five-Level Classification Inter-Tracer Agreement by Scan Interval

	MK- FTP	MK - RO	MK - PI	FTP - RO	FTP - PI	RO - PI
**≤ 45 days interval**						
Participants, n	483	104	98	85	85	96
Agreement, No. (%)	400 (82.8)	85 (81.7)	73 (74.5)	70 (82.4)	69 (81.2)	75 (78.1)
**45 – 90 days interval**						
Participants, n	111	32	35	35	27	11
No. (%)	87 (78.4)	29 (90.6)	25 (71.4)	29 (82.9)	21 (77.8)	11 (100.0)
**> 90 days interval**						
Participants, n	50	14	16	11	19	24
No. (%)	42 (84.0)	11 (78.6)	14 (87.5)	10 (90.9)	14 (73.7)	22 (91.7)

Abbreviations: MK, [^{18}F]MK-6240; FTP, [^{18}F]flortaucipir; RO, [^{18}F]RO948; PI, [^{18}F]PI-2620.
Agreements using majority vote of the three raters.

© 2026 Ruppert E et al; HEAD study group. *JAMA Neurology*.

eTable 14. PI-2620 Classification With and Without Composite 1

	**PI-2620 without C1**	**PI-2620 with C1**
**Binary Positivity,** No. (%)	30 (20.1)	57 (38.3)
**Class distribution**		
Negative	119 (79.9)	92 (61.7)
Atypical	5 (3.3)	1 (0.7)
Low	0 (0)	28 (18.8)
Moderate	8 (5.4)	10 (6.7)
High	17 (11.4)	18 (12.1)

Abbreviations: C1, composite 1; PI, [^{18}F]PI-2620.
Based on majority vote of the three raters.

© 2026 Ruppert E et al; HEAD study group. *JAMA Neurology*.

eTable 15. Demographics of Individuals Classified as Atypical Tau Burden Using the Harmonized Framework

Atypical radiotracer	Other radiotracers	Age	Sex	Educ	APOE ε4 status	Clinical Diagnosis	Aβ status	Centiloid	Memory	Language	EF	Attention	VS
FTP	MK: Neg; RO: Neg; PI: Neg	72	M	18	Non-carrier	MCI	Neg	-2.91	-0.27	-0.22	0.15	0.39	0.23
FTP	MK: Neg; RO: Neg; PI: Neg	70	M	19	Non-carrier	CU	Neg	2.67	-0.82	-0.66	0.00	-0.06	0.15
RO	MK: Neg; FTP: Neg; PI: Neg	63	M	15	Heterozygous	MCI	Neg	9.88	-1.12	-0.04	-0.68	-0.65	0.30
RO	MK: Neg; FTP:Neg; PI: Neg	64	F	13	Homozygous	Dementia	Pos	64.91	-0.42	-0.58	1.28	0.70	0.39
MK	FTP: Neg	70	M	12	Non-carrier	MCI	Neg	-4.31	0.04	-0.01	0.20	0.16	-1.73
FTP	MK: Mod	76	M	16	Heterozygous	CU	Pos	113.17	-1.72	-0.92	-0.04	-0.63	1.13
FTP	MK: Mod	78	M	20	Non-carrier	Dementia	Pos	57.41	-1.83	-4.48	-2.15	-6.41	-2.10
FTP	MK: Mod	84	F	12	Non-carrier	MCI	Pos	55.71	-1.23	-0.80	-0.34	-0.23	0.64
FTP	MK: High	61	F	18	Non-carrier	CU	Pos	165.19	0.24	0.16	-0.60	0.30	0.84
FTP	MK: High	67	F	18	Heterozygous	CU	Pos	55.98	0.03	-0.69	-0.12	0.07	0.14
PI	MK: Mod; FTP: Mod; RO: Mod	62	M	16	Heterozygous	MCI	Pos	51.67	-0.23	-0.23	-0.70	-0.11	-0.52
PI	MK: High; FTP: Mod; RO: Mod	68	M	21	Homozygous	Dementia	Pos	168.46	-2.95	-1.62	-0.65	0.23	-1.49
PI	MK: High; FTP: High; RO: High	73	F	14	Heterozygous	MCI	Pos	154.31	-0.15	-0.35	-0.04	-0.17	0.42
PI	MK: High; FTP: High; PI: High	68	M	15	Heterozygous	MCI	Pos	141.35	-2.36	-0.08	-0.03	-0.59	-0.41
PI	MK: High; FTP: High; RO: High	70	F	16	Heterozygous	MCI	Pos	150.45	-1.61	-0.23	0.72	0.75	0.28

Each row represents one participant classified as atypical by a single radiotracer. Other radiotracers column shows the harmonized classification from all other available tracers for that participant. Cognitive domain scores (memory, language, executive function, attention and visuospatial) are z-scores adjusted for age, sex, and education using UDS-3 normative regression models.

Abbreviations: Aβ, amyloid-β; APOE ε4, apolipoprotein ε4 allele; CU, cognitively unimpaired; EF, executive function; FTP, [^{18}F]flortaucipir; MCI, mild cognitive impairment; MK, [^{18}F]MK-6240; Mod, moderate; Neg, negative; PI, [^{18}F]PI-2620; Pos, positive; RO, [^{18}F]RO948; VS, visuospatial.

© 2026 Ruppert E et al; HEAD study group. *JAMA Neurology*.

eTable 16. Binary Tau PET Positivity Across Clinical Diagnoses

	**Harmonized MK-6240**	**Harmonized FTP**	**Regulatory-approved FTP**	**Harmonized RO948**	**Harmonized PI-2620**
**All participants**					
CU	73 (19.8)	49 (13.5)	26 (7.1)	5 (7.0)	0 (0)
MCI	156 (63.7)	123 (55.2)	100 (44.8)	35 (58.3)	18 (29.0)
Dementia	56 (83.6)	47 (82.5)	45 (78.9)	14 (73.7)	12 (70.6)
**Aβ-positives participants**					
CU	42 (48.8)	27 (32.1)	20 (23.8)	3 (15.8)	0 (0)
MCI	131 (84.5)	110 (76.4)	97 (67.4)	25 (75.8)	18 (47.4)
Dementia	53 (94.6)	46 (90.2)	45 (88.2)	14 (93.3)	12 (85.7)
**Aβ-negatives participants**					
CU	31 (11.0)	22 (7.9)	6 (2.1)	2 (3.8)	0 (0)
MCI	25 (27.8)	13 (16.5)	3 (3.8)	10 (37.0)	0 (0)
Dementia	3 (27.3)	1 (16.7)	0 (0)	0 (0)	0 (0)

Abbreviations: CU, cognitively unimpaired; MCI, mild cognitive impairment; MK-6240, [^{18}F]MK-6240; FTP, [^{18}F]flortaucipir; RO948, [^{18}F]RO948; PI-2620, [^{18}F]PI-2620.
Among tau positives in the Aβ-negatives group: Harmonized MK-6240 - low tau burden (n=50), moderate tau burden (n=8), atypical (n=1), mean Centiloids 2.1 (SD 9.7); Harmonized FTP - low tau burden (n=28), moderate tau burden (n=4), high tau burden (n=2), atypical (n=2), mean Centiloids 4.1 (SD 11.0); Harmonized RO948 - low tau burden (n=7), moderate tau burden (n=2), high tau burden (n=1), atypical (n=1), mean Centiloids 5.4 (SD 5.7).
Positivity based on majority vote of the three raters.

© 2026 Ruppert E et al; HEAD study group. *JAMA Neurology*.

eTable 17. Reclassification From Regulatory-Approved to Harmonized Framework

	**Regulatory-approved Negative**	**Regulatory-approved Positive**	**Total**
**Harmonized Negative**	413	12	425
**Harmonized Low**	49	9	58
**Harmonized Moderate**	5	57	62
**Harmonized High**	3	89	92
**Harmonized Atypical**	3	4	7
**Total**	473	171	644

Based on the majority vote among three independent blinded raters.

© 2026 Ruppert E et al; HEAD study group. *JAMA Neurology*.

eTable 18. Cognitive Composites (UDS-3) Across Harmonized [^{18}F]MK-6240 Classifications

Composite	Contrast	Adjusted estimate (95% CI)	P value
Memory	Negative - Low	0.2 (-0.08 to 0.5)	ns
	Negative - Moderate	1.0 (0.6 to 1.3)	<.001
	Negative - High	2.1 (1.8 to 2.4)	<.001
	Low - Moderate	0.8 (0.4 to 1.2)	<.001
	Low - High	1.9 (1.6 to 2.3)	<.001
	Moderate - High	1.2 (0.8 to 1.6)	<.001
Language	Negative - Low	0.3 (0.01 to 0.5)	<.05
	Negative - Moderate	0.4 (0.1 to 0.7)	<.05
	Negative - High	1.3 (1.1 to 1.6)	<.001
	Low - Moderate	0.1 (-0.3 to 0.5)	ns
	Low - High	1.1 (0.7 to 1.4)	<.001
	Moderate - High	0.9 (0.6 to 1.3)	<.001
Executive Function	Negative - Low	0.4 (0.1 to 0.6)	<.01
	Negative - Moderate	0.4 (0 to 0.72)	<.05
	Negative - High	1.4 (1.2 to 1.7)	<.001
	Low - Moderate	0 (-0.4 to 0.4)	ns
	Low - High	1.1 (0.7 to 1.4)	<.001
	Moderate - High	1.1 (0.7 to 1.5)	<.001
Attention	Negative - Low	0.3 (-0.04 to 0.7)	ns
	Negative - Moderate	0.5 (0.1 to 1.0)	<.05
	Negative - High	1.6 (1.2 to 1.9)	<.001
	Low - Moderate	0.2 (-0.4 to 0.7)	ns
	Low - High	1.3 (0.8 to 1.7)	<.001
	Moderate - High	1.1 (0.6 to 1.6)	<.001
Visuospatial	Negative - Low	0.3 (-0.2 to 0.8)	ns
	Negative - Moderate	0.1 (-0.6 to 0.7)	ns
	Negative - High	1.9 (1.4 to 2.4)	<.001
	Low - Moderate	-0.2 (-0.9 to 0.6)	ns
	Low - High	1.6 (1.0 to 2.3)	<.001
	Moderate - High	1.8 (1.0 to 2.5)	<.001

Sample sizes: Memory (n = 669); Language (n = 670); Executive Function (n = 671); Attention (n = 671); Visuospatial (n = 664). Memory composite was calculated based on the mean z-score of craft stories verbatim delayed recall, craft stories paraphrase delayed recall, and Benson figure delayed recall. Language was calculated based on the mean z-score of category verbal fluency, letter verbal fluency, and MINT total score. Executive Function is based on the mean z-score of trail making test B (reversed) and digit span backward. Attention is the mean z-score of trail making test A (reversed) and digit span forward. Visuospatial is based on the z-score of Benson Figure copy. All z-scores adjusted for age, sex and education using the UDS-3 normative regression models.[2] Adjusted estimates reflect pairwise mean differences from linear models with Tukey correction for multiple comparisons.

© 2026 Ruppert E et al; HEAD study group. *JAMA Neurology*.

eTable 19. Cognitive Composites (UDS-3) Across Harmonized [^{18}F]flortaucipir Classifications

Composite	Contrast	Adjusted estimate (95 % CI)	P value
Memory	Negative - Low	0.3 (-0.02 to 0.7)	ns
	Negative - Moderate	1.4 (1.0 to 1.7)	<.001
	Negative - High	2.2 (1.9 to 2.5)	<.001
	Low - Moderate	1.0 (0.6 to 1.5)	<.001
	Low - High	1.9 (1.5 to 2.3)	<.001
	Moderate - High	0.9 (0.5 to 1.3)	<.001
Language	Negative - Low	0 (-0.3 to 0.4)	ns
	Negative - Moderate	0.6 (0.3 to 1.0)	<.001
	Negative - High	1.4 (1.1 to 1.7)	<.001
	Low - Moderate	0.6 (0.2 to 1.1)	<.01
	Low - High	1.3 (1.0 to 1.7)	<.001
	Moderate - High	0.7 (0.3 to 1.1)	<.001
Executive Function	Negative - Low	0.2 (-0.2 to 0.5)	ns
	Negative - Moderate	0.6 (0.3 to 1.0)	<.001
	Negative - High	1.5 (1.2 to 1.8)	<.001
	Low - Moderate	0.5 (0 to 0.9)	ns
	Low - High	1.3 (0.9 to 1.7)	<.001
	Moderate - High	0.8 (0.4 to 1.3)	<.001
Attention	Negative - Low	0.3 (-0.2 to 0.7)	ns
	Negative - Moderate	0.8 (0.3 to 1.2)	<.001
	Negative - High	1.6 (1.3 to 2.0)	<.001
	Low - Moderate	0.5 (-0.1 to 1.1)	ns
	Low - High	1.4 (0.9 to 1.9)	<.001
	Moderate - High	0.9 (0.4 to 1.4)	<.001
Visuospatial	Negative - Low	0.1 (-0.5 to 0.8)	ns
	Negative - Moderate	0.4 (-0.2 to 1.1)	ns
	Negative - High	1.9 (1.4 to 2.5)	<.001
	Low - Moderate	0.3 (-0.5 to 1.2)	ns
	Low - High	1.8 (1.1 to 2.6)	<.001
	Moderate - High	1.5 (0.7 to 2.3)	<.001

Sample sizes: Memory (n = 626); Language (n = 627); Executive Function (n = 628); Attention (n = 628); Visuospatial (n = 621). Memory composite was calculated based on the mean z-score of craft stories verbatim delayed recall, craft stories paraphrase delayed recall, and Benson figure delayed recall. Language was calculated based on the mean z-score of category verbal fluency, letter verbal fluency and MINT total score. Executive Function is based on the mean z-score of trail making test B (reversed) and digit span backward. Attention is the mean z-score of trail making test A (reversed) and digit span forward. Visuospatial is based on the z-score of Benson Figure copy. All z-scores adjusted for age, sex and education using the UDS-3 normative

© 2026 Ruppert E et al; HEAD study group. *JAMA Neurology*.

regression models.[2] Adjusted estimates reflect pairwise mean differences from linear models with Tukey correction for multiple comparisons.

© 2026 Ruppert E et al; HEAD study group. *JAMA Neurology*.

eFigure 1. Study Sample Flowchart

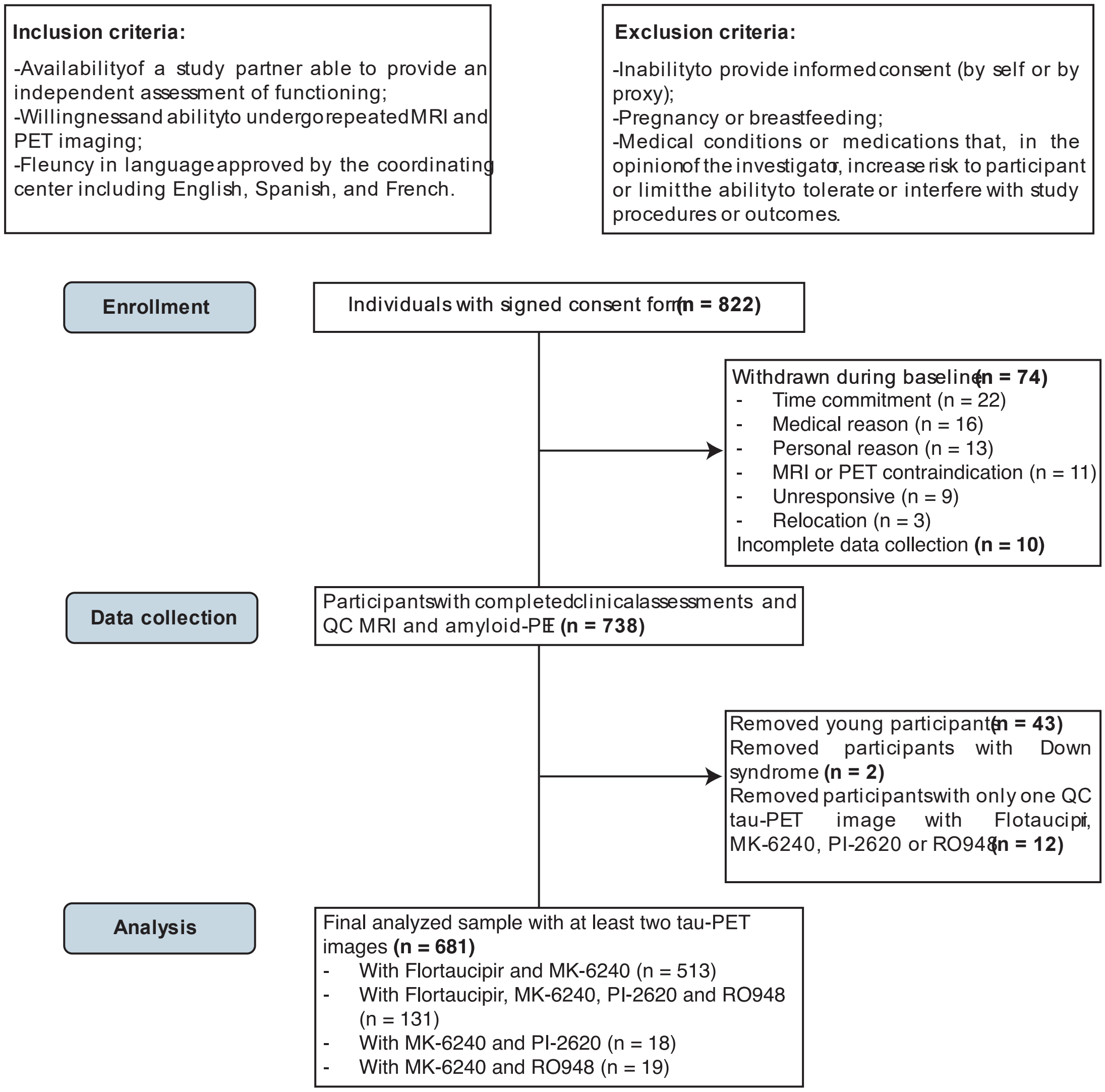

Abbreviations: PET, positron emission tomography; QC, quality control; MRI, magnetic resonance imaging; MK-6240, [^{18}F]MK-6240; FTP, [^{18}F]flortaucipir; RO948, [^{18}F]RO948; PI-2620, [^{18}F]PI-2620.

© 2026 Ruppert E et al; HEAD study group. *JAMA Neurology*.

eFigure 2. Regional Abnormality Frequencies Across Tau PET Radiotracers Informing Composite Definitions

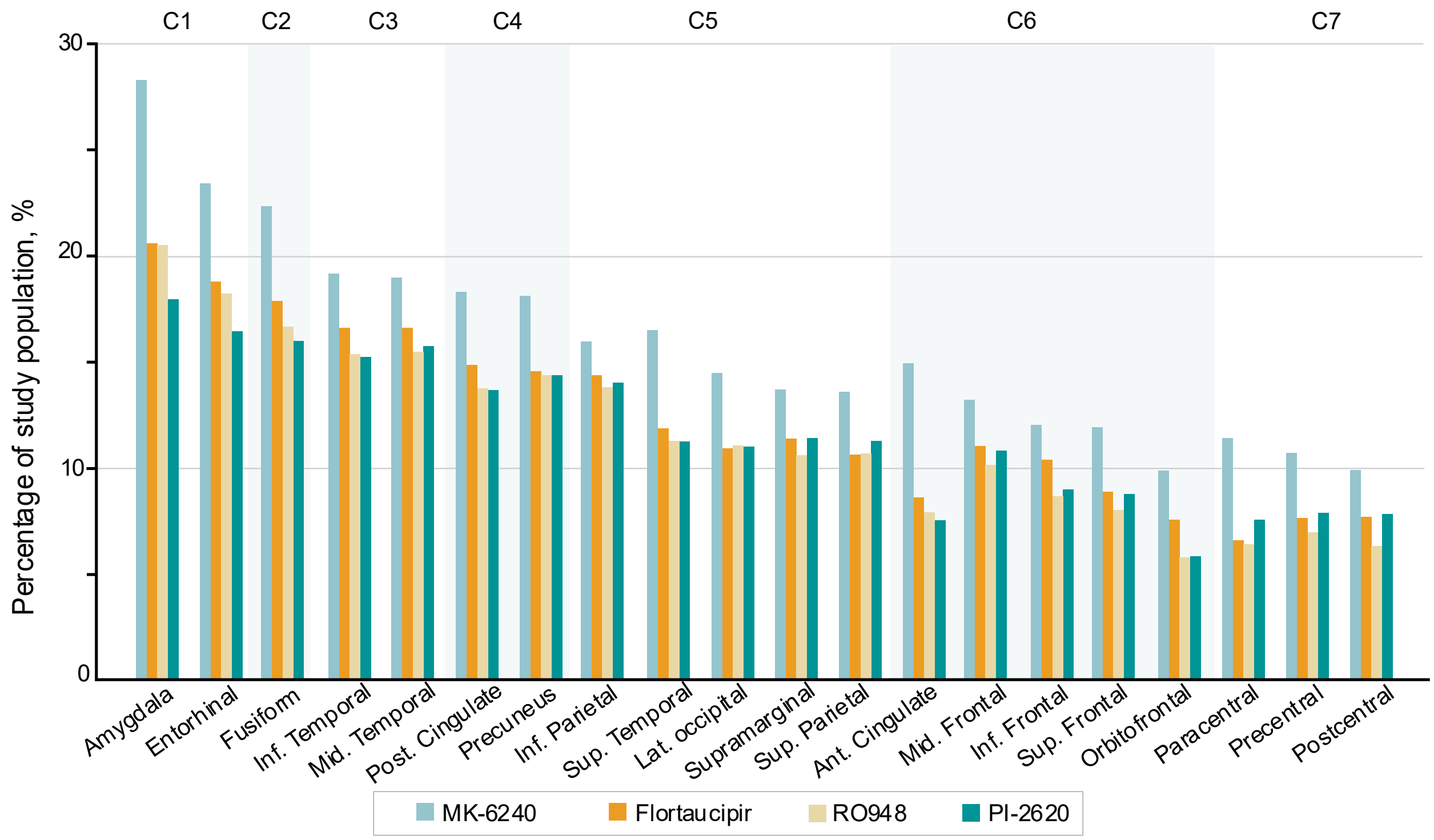

Abbreviations: ant., anterior; inf., inferior; mid., middle; lat., lateral; sup., superior; C1, composite 1; C2, composite 2; C3, composite 3; C4, composite 4; C5, composite 5; C6, composite 6; C7, composite 7; ; ROI, regions of interest; PET, positron emission tomography. Bar chart displays the percentage of participants with abnormal tau uptake in each ROI stratified by tracer. Data are from the four-tracer subsample (n=131). Abnormality was defined as uptake exceeding 2 standard deviations above the mean of cognitively unimpaired, amyloid-negative participants (n=51). ROIs with similar abnormality patterns across tracers and anatomical proximity were grouped into seven composite regions (C1–C7): C1 (amygdala, entorhinal cortex), C2 (fusiform gyrus), C3 (middle and inferior temporal cortices), C4 (posterior cingulate, precuneus), C5 (inferior parietal, lateral occipital, superior temporal, superior parietal, supramarginal cortices), C6 (anterior cingulate, middle frontal, superior frontal, inferior frontal, orbitofrontal cortices), and C7 (precentral, paracentral, postcentral cortices).

eFigure 3. Regional Abnormality Prevalence at 1.5, 2.0, and 2.5 SD Thresholds Across Radiotracers

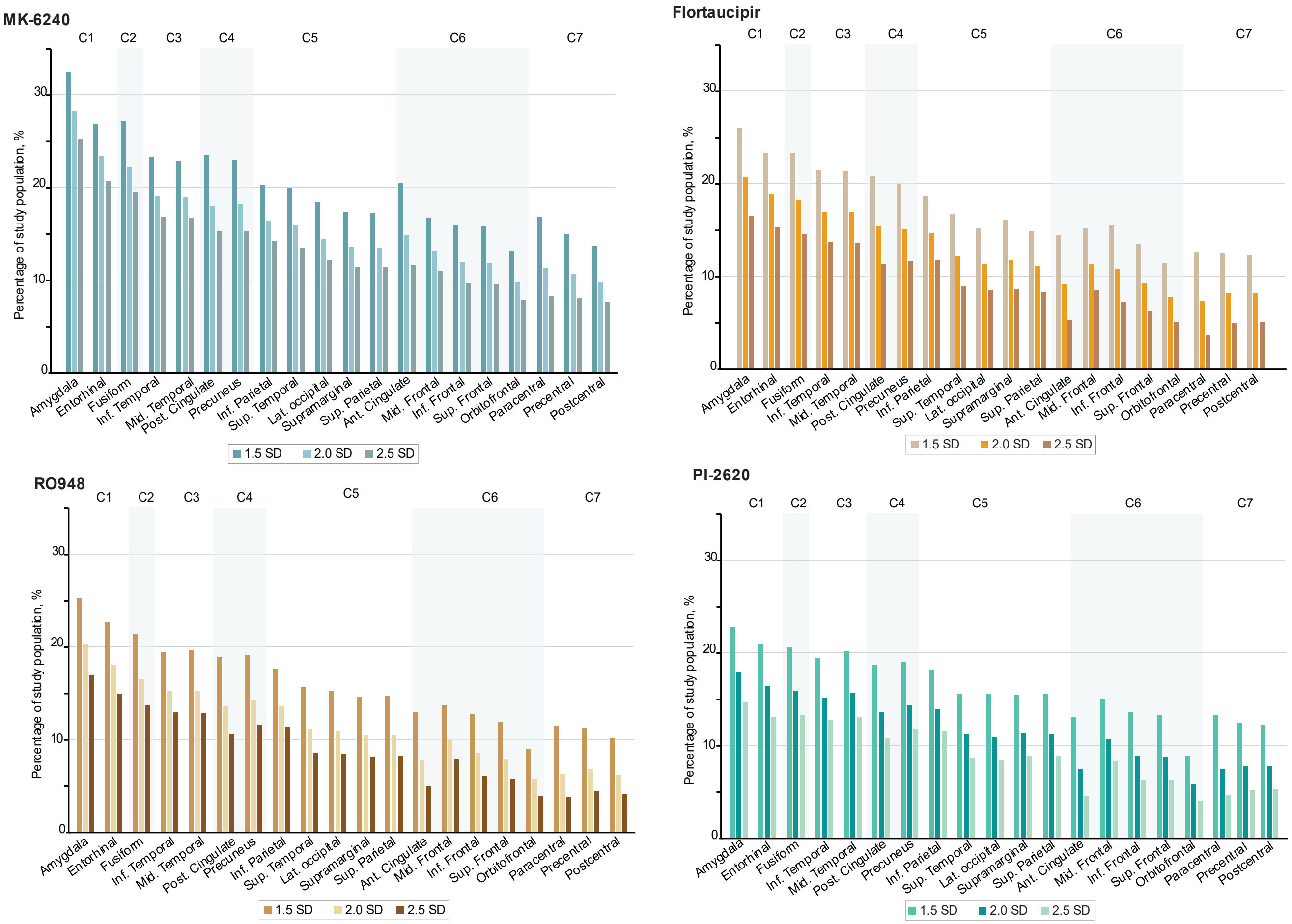

Abbreviations: ant., anterior; inf., inferior; mid., middle; lat., lateral; sup., superior; C1, composite 1; C2, composite 2; C3, composite 3; C4, composite 4; C5, composite 5; C6, composite 6; C7, composite 7; ROI, regions of interest;

Percentage of the study population with voxel-level abnormality in each of the ROIs at 1.5 SD, 2.0 SD, and 2.5 SD thresholds above the cognitively unimpaired reference mean, shown separately for [^{18}F]MK-6240, [^{18}F]flortaucipir, [^{18}F]RO948, and [^{18}F]PI-2620.Across all four tracers and all three thresholds, the rank ordering of regional involvement remained consistent. Higher SD thresholds reduced overall prevalence but preserved the hierarchical pattern.

© 2026 Ruppert E et al; HEAD study group. *JAMA Neurology*.

eFigure 4. Examples of Regions With Frequent Off-Target Binding Excluded From the Harmonized Visual Reading Framework

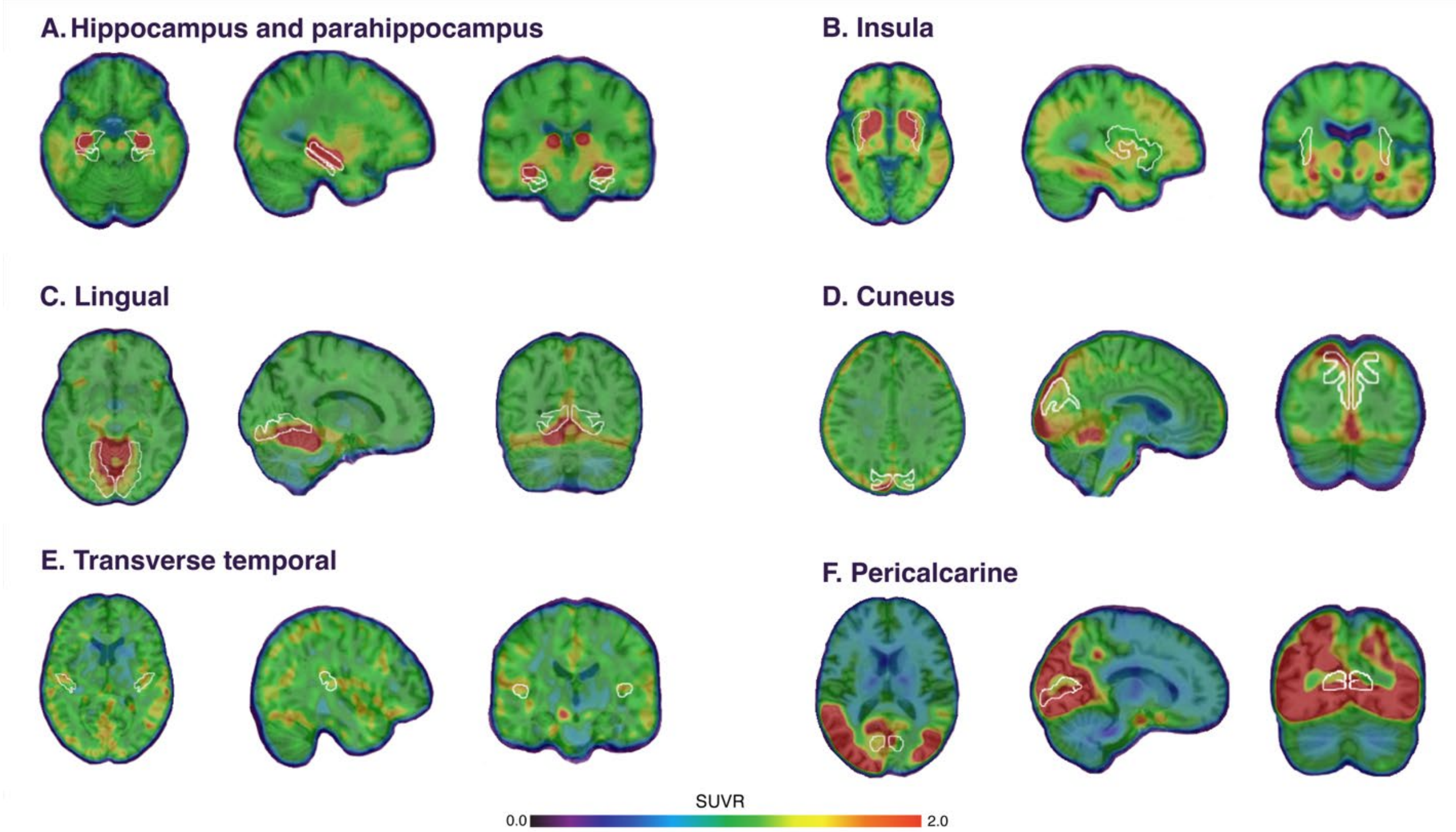

Abbreviations: Aβ, amyloid-β; CU, cognitively unimpaired; MCI, mild cognitive impairment.

Each panel shows a representative tau PET image illustrating off-target binding patterns that informed region exclusion decisions. Panel A, [^{18}F]Flortaucipir off-target binding in the hippocampus, complicating visual assessment of the adjacent parahippocampal gyrus; 69-year-old male; CU, Aβ-negative. Panel B, [^{18}F]Flortaucipir off-target binding in the putamen, with potential spillover into the insula causing visual ambiguity; 80-year-old male; MCI, Aβ-negative. Panel C, [^{18}F]MK-6240 meningeal binding extending into the tentorial space between cerebellum and cerebrum, affecting interpretation of the lingual region; 72-year-old male; CU, Aβ-negative. Panel D, [^{18}F]MK-6240 meningeal binding adjacent to the cuneus; 66-year-old female; CU, Aβ-negative. Panel E, the small size of the transverse temporal region may hinder accurate visual interpretation; 58-year-old male; CU, Aβ-negative. Panel F, [^{18}F]MK-6240 spillover from neighboring regions into the pericalcarine, despite absence of tau deposition in precentral, postcentral, and paracentral regions, suggesting spillover rather than true tau accumulation; 72-year-old female; dementia, Aβ-positive.

© 2026 Ruppert E et al; HEAD study group. *JAMA Neurology*.

eFigure 5. Composite-Specific Visual Reference Masks

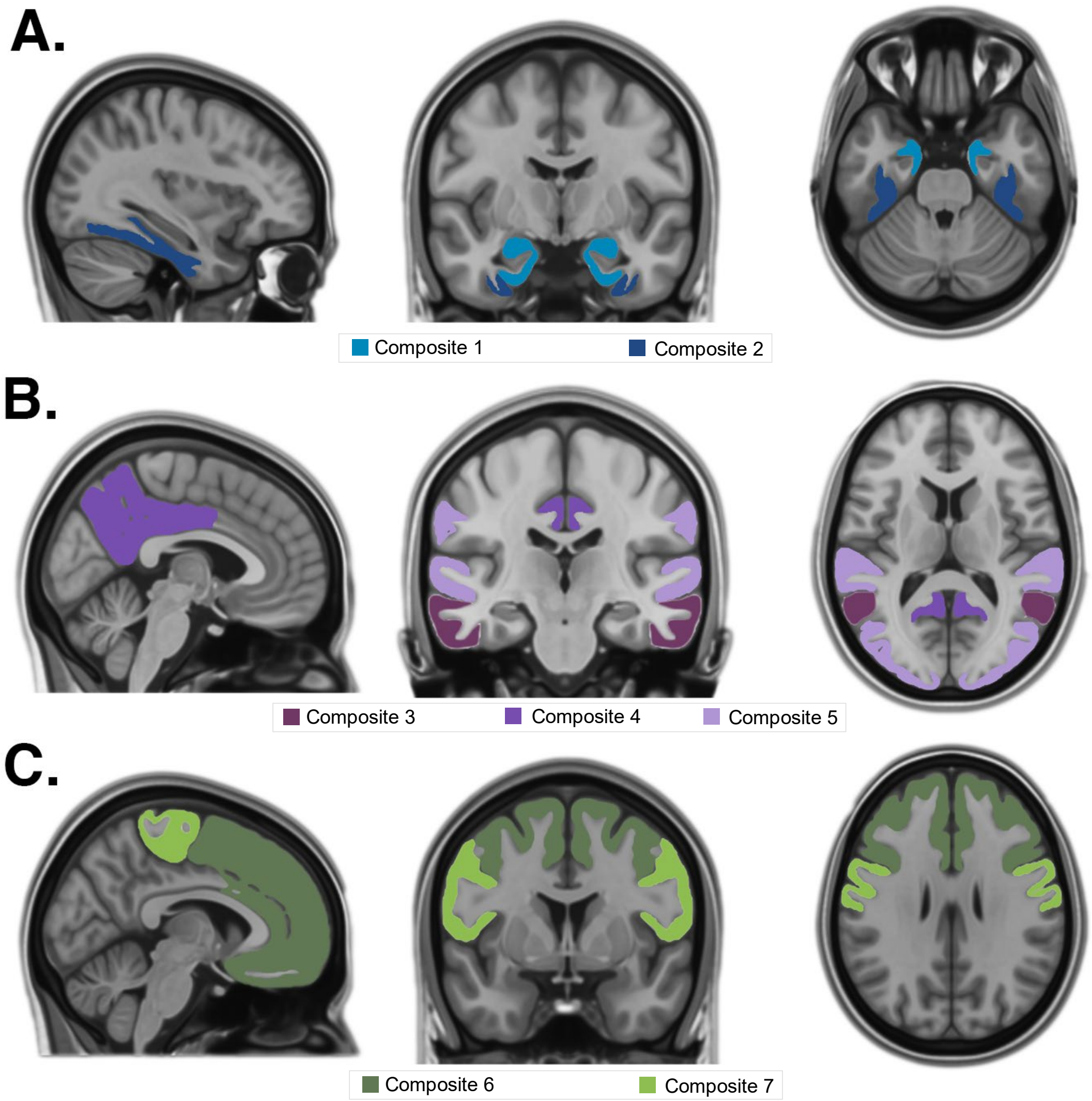

Abbreviations: MRI, magnetic resonance imaging

Sagittal, coronal, and axial views show composite-specific visual reference masks overlaid on a T1-weighted MRI template in MNI-152 space. These masks are integrated into the web-based visual reading platform to guide raters through binary classification (positive/negative) of each composite. The masks serve as anatomical references to aid boundary delineation and do not independently determine positivity. The composite regions were: (1) amygdala and entorhinal; (2) fusiform gyrus; (3) middle and inferior temporal; (4) posterior cingulate and precuneus; (5) inferior parietal, lateral occipital, superior temporal, superior parietal, and supramarginal; (6) anterior cingulate, middle frontal, superior frontal, inferior frontal, and orbitofrontal; and (7) precentral, paracentral, and postcentral. Panel A correspond to early detection composites and in Panel B and C correspond to staging composites.

© 2026 Ruppert E et al; HEAD study group. *JAMA Neurology*.

eFigure 6. Harmonized Tau PET Visual Reading Tool

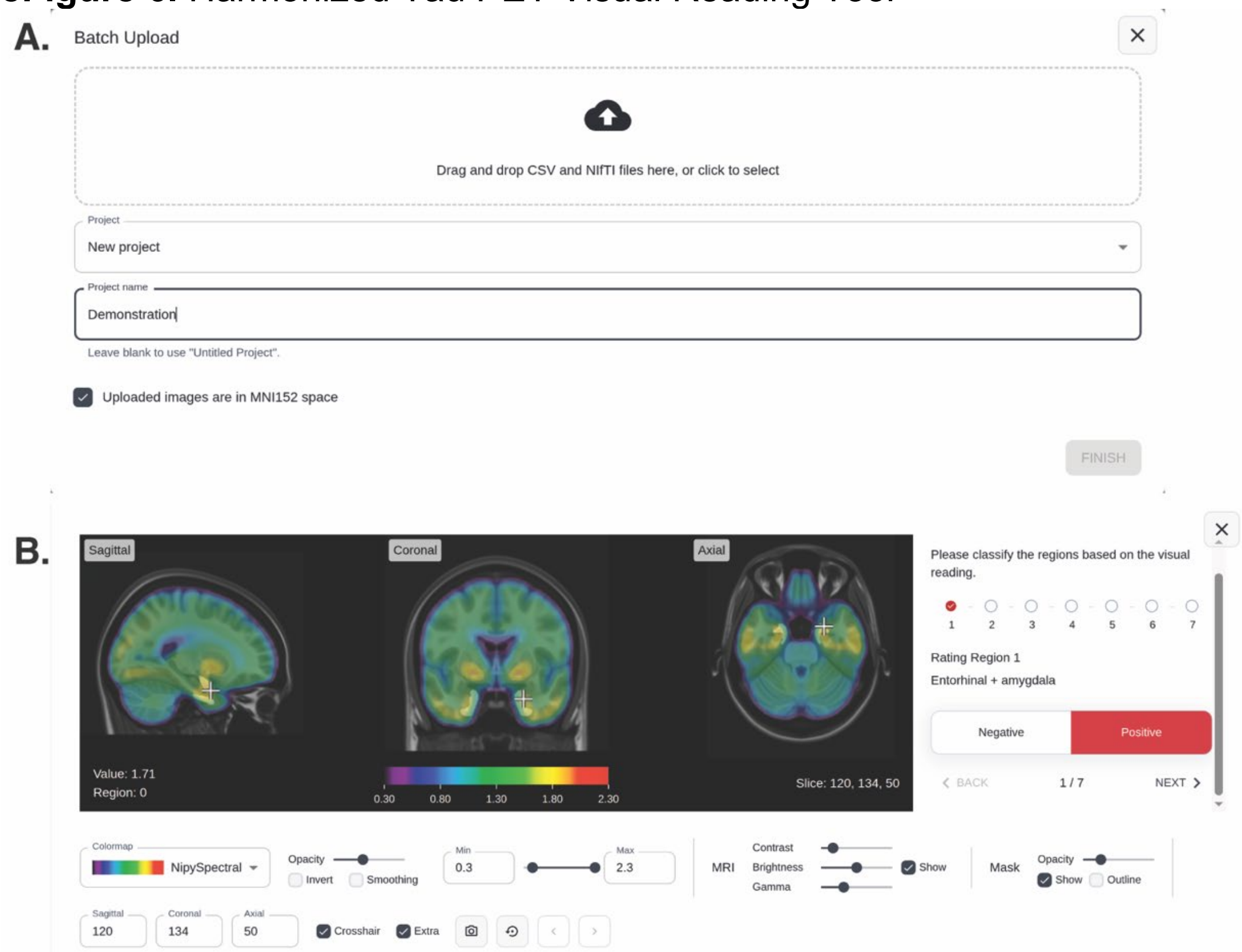

Abbreviations: CSV, comma-separated values; MRI, magnetic resonance imaging; PET, positron emission tomography; SUVR, standardized uptake value ratio.

The batch upload interface (Panel A) allows raters to upload a CSV file with subject IDs, PET and MRI file paths along with the corresponding NIfTI (.nii) files. Images must be normalized to the ICBM/MNI152 standard space for the anatomical reference masks to align correctly. If no subject-specific MRI is available, the tool loads a template MRI automatically. The main reading interface displays the tau PET SUVR image overlaid on the structural MRI in sagittal, coronal, and axial planes simultaneously (Panel B). Raters navigate through slices in each plane and can toggle the anatomical reference mask overlay on or off. For each of the seven composite regions, raters select negative or positive. Visualization settings including colormap, opacity, smoothing, contrast, and gamma are adjustable to support image interpretation.

© 2026 Ruppert E et al; HEAD study group. *JAMA Neurology*.

eFigure 7. Mean Tau PET ([18F]MK-6240) SUVR Images by Harmonized Five-Level Classification

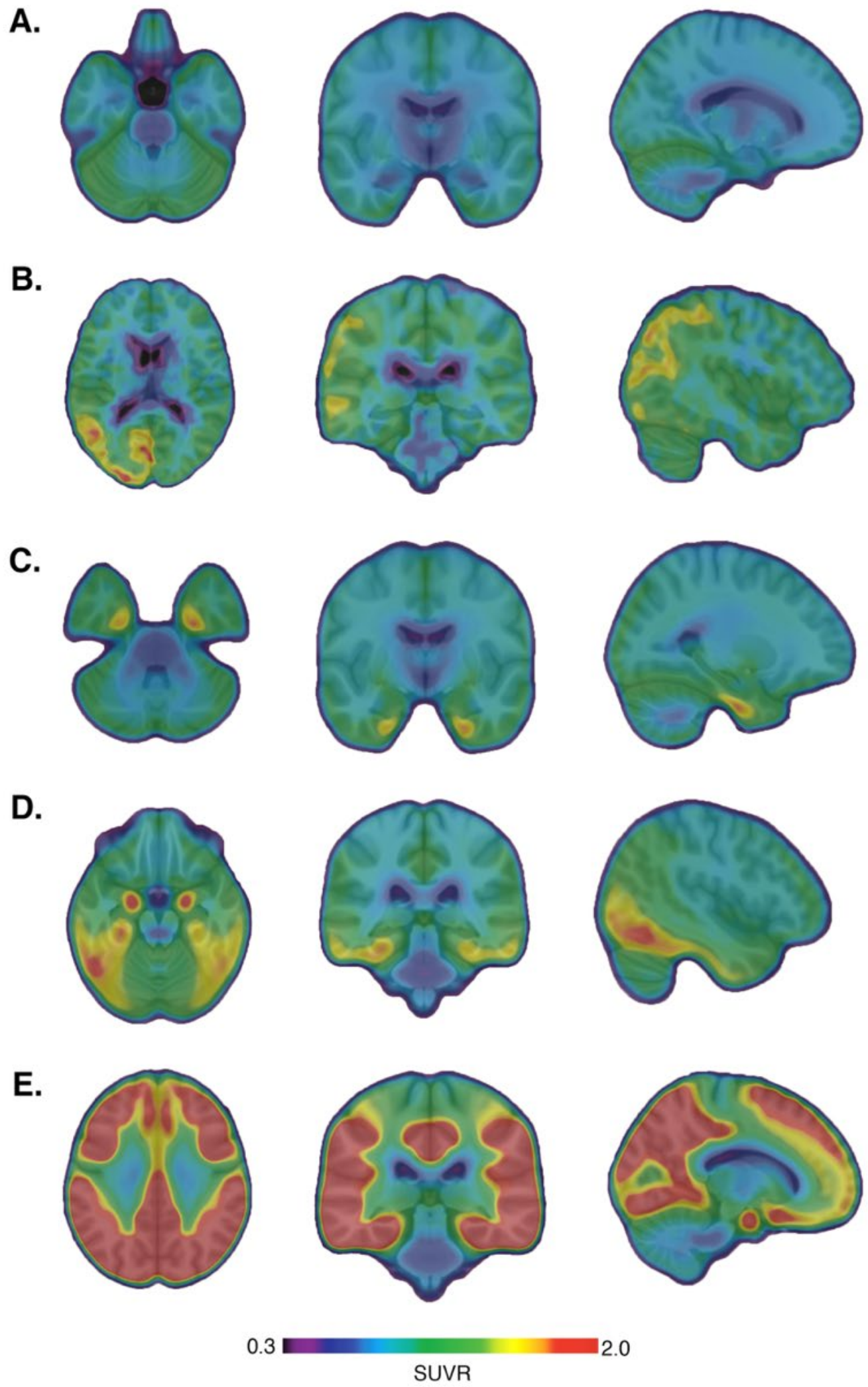

Abbreviations: PET, positron emission tomography; SUVR, standardized uptake value ratio.
Average [18F]MK-6240 SUVR images are shown in axial, coronal, and sagittal planes for each of the five-level classification groups: negative (Panel A, n=396), atypical (Panel B, n=1), low tau burden (Panel C, n=109), moderate tau burden (Panel D, n=59), and high tau burden (Panel E, n=116). Images reflect the mean SUVR across all participants assigned to each class by majority vote.

© 2026 Ruppert E et al; HEAD study group. *JAMA Neurology*.

eFigure 8. Mean Tau PET ([¹⁸F]flortaucipir) SUVR Images by Harmonized Five-Level Classification

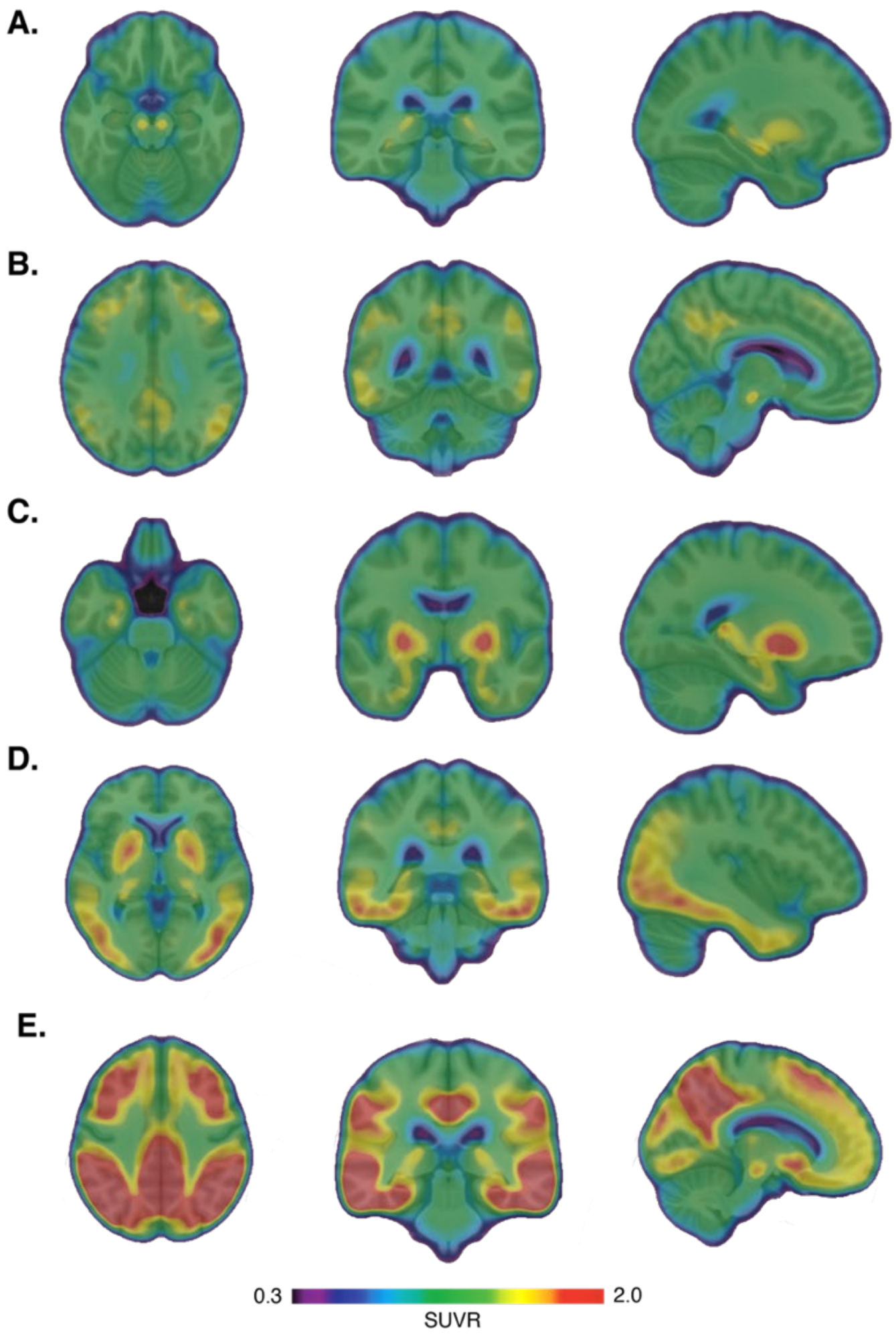

Abbreviations: PET, positron emission tomography; SUVR, standardized uptake value ratio.
Average [¹⁸F]flortaucipir SUVR images are shown in axial, coronal, and sagittal planes for each of the five-level classification groups: negative (Panel A, n=425), atypical (Panel B, n=7), low tau burden (Panel C, n=58), moderate tau burden (Panel D, n=62), and high tau burden (Panel E, n=92). Images reflect the mean SUVR across all participants assigned to each class by majority vote.

© 2026 Ruppert E et al; HEAD study group. *JAMA Neurology*.

eFigure 9. Mean Tau PET ([^{18}F]RO948) SUVR Images by Harmonized Five-Level Classification

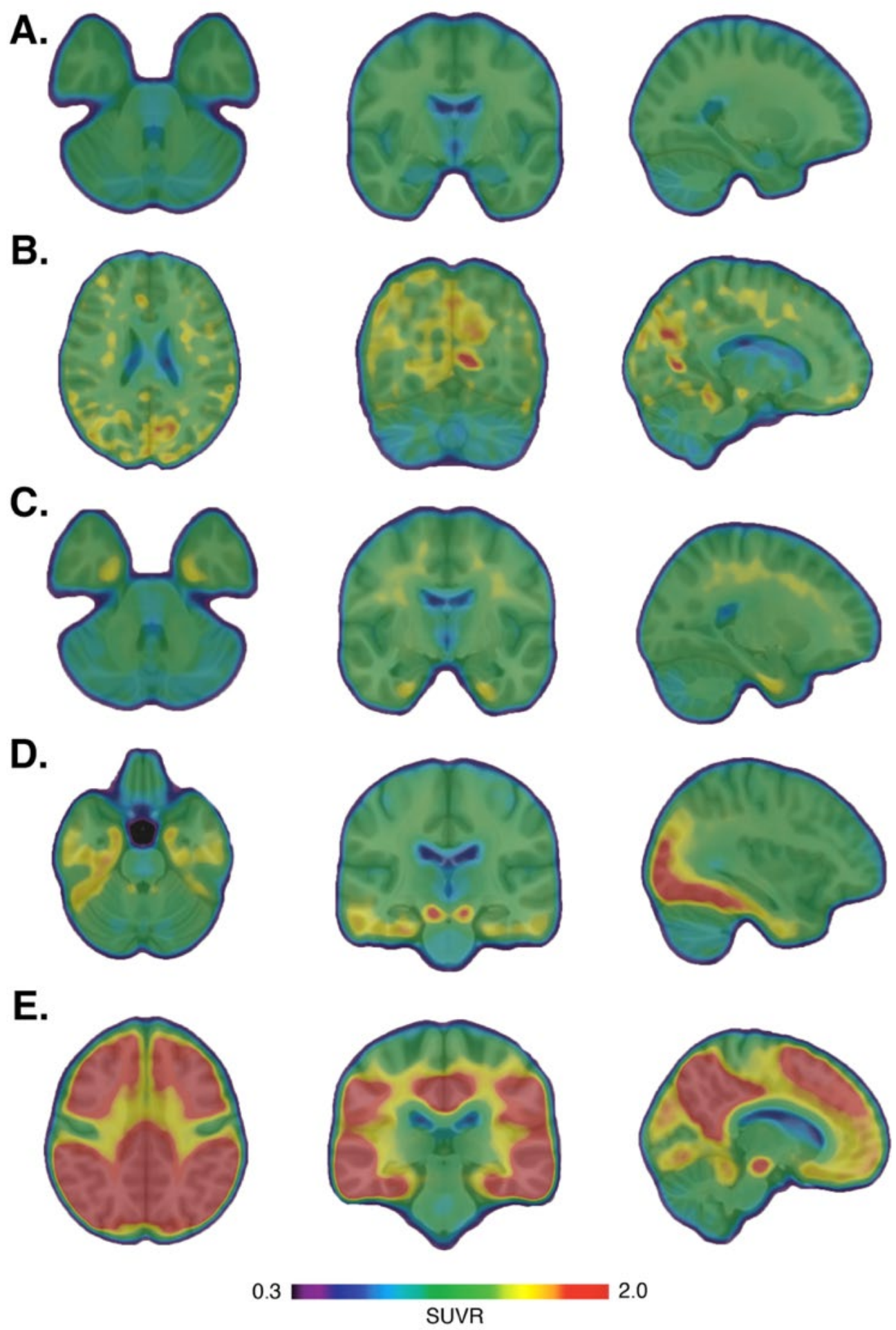

Abbreviations: PET, positron emission tomography; SUVR, standardized uptake value ratio.
Average [^{18}F]RO948 SUVR images are shown in axial, coronal, and sagittal planes for each of the five-level classification groups: negative (Panel A, n=96), atypical (Panel B, n=2), low tau burden (Panel C, n=17), moderate tau burden (Panel D, n=12), and high tau burden (Panel E, n=22). Images reflect the mean SUVR across all participants assigned to each class by majority vote

© 2026 Ruppert E et al; HEAD study group. *JAMA Neurology*.

eFigure 10. Mean Tau PET ([^{18}F]PI-2620) SUVR Images by Harmonized Five-Level Classification

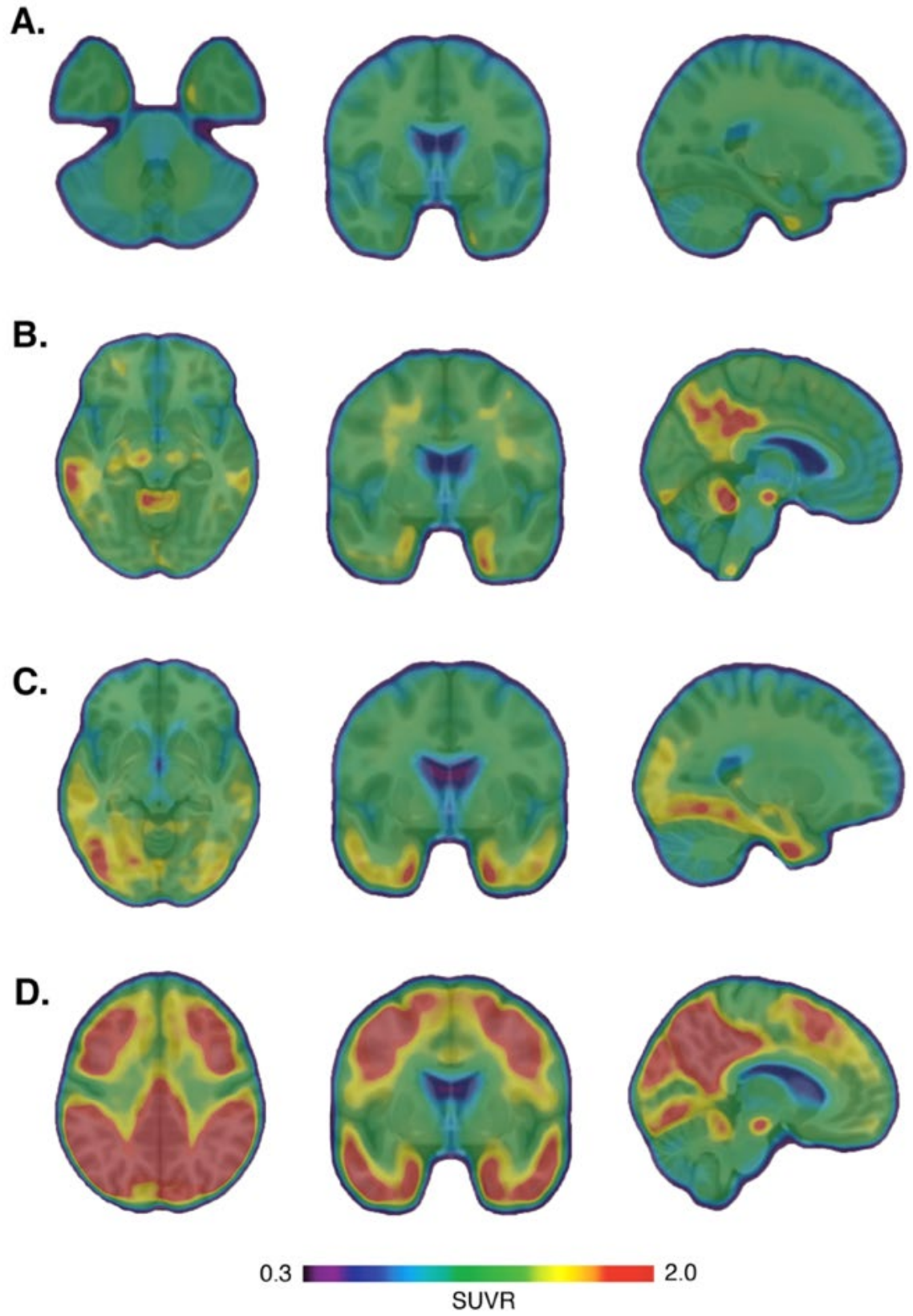

Abbreviations: PET, positron emission tomography; SUVR, standardized uptake value ratio.
Average [^{18}F]PI-2620 SUVR images are shown in axial, coronal, and sagittal planes for each of the five-level classification groups. negative (Panel A, n=119), atypical (Panel B, n=5), moderate tau burden (Panel C, n=8), and high tau burden (Panel D, n=17). No low cases were identified within this sample with [^{18}F]PI-2620 after removal of composite 1. Images reflect the mean SUVR across all participants assigned to each class by majority vote.

© 2026 Ruppert E et al; HEAD study group. *JAMA Neurology*.

eFigure 11. Mean Tau PET SUVR Images by Harmonized Five-Level Classification Across the Four Radiotracers

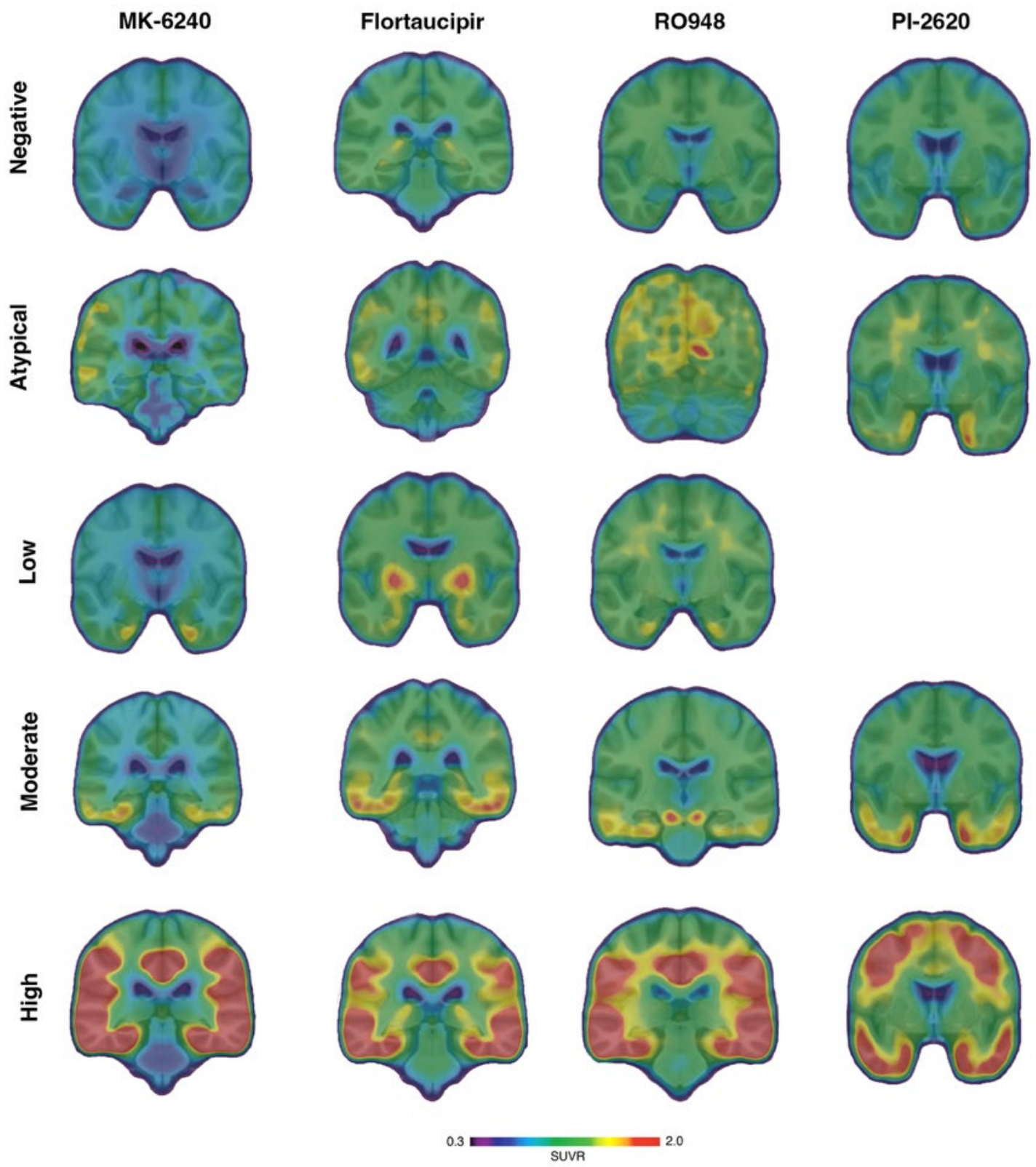

Abbreviations: PET, positron emission tomography; SUVR, standardized uptake value ratio.
Average SUVR images are shown in coronal view for each of the five-level tau burden classification groups (negative, atypical, low, moderate, and high) across all four tau PET tracers: [^{18}F]MK-6240, [^{18}F]Flortaucipir, [^{18}F]RO948, and [^{18}F]PI-2620. Each row represents one classification group, and each column represents one tracer. Images reflect the mean SUVR across all participants assigned to each class by majority vote. No low cases were identified within this sample with [^{18}F]PI-2620.

© 2026 Ruppert E et al; HEAD study group. *JAMA Neurology*.

eFigure 12. Inter-rater Cohen's Kappa for Tau PET Regional Composites, Five-Level Classification, and Binary Positivity

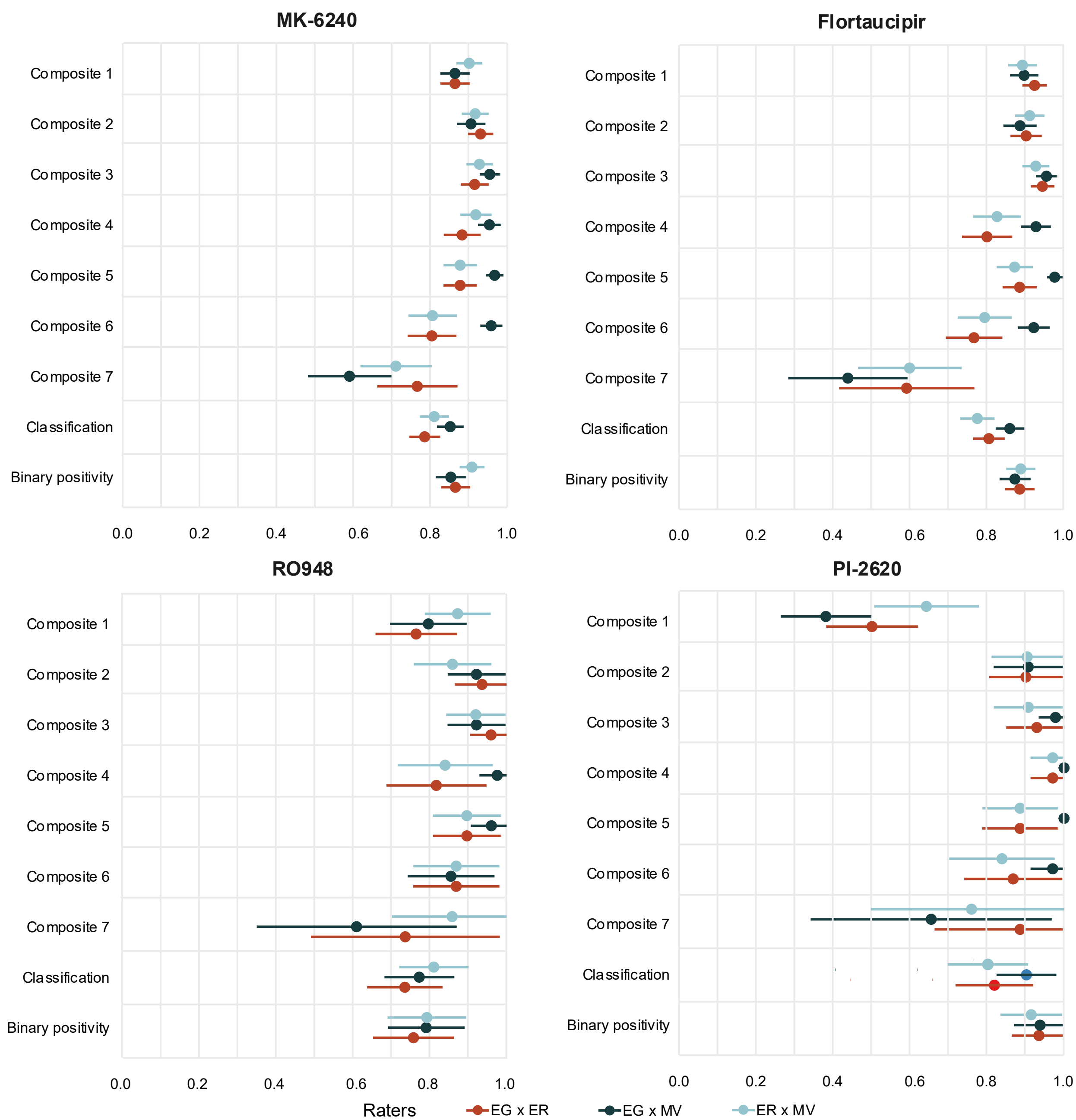

Abbreviations: κ, kappa; PET, positron emission tomography.

Cohen's κ and 95% confidence intervals are shown for each of the seven composite regions, five-level classification, and binary tau PET positivity, separately for [18F]MK-6240, [18F]flortaucipir, [18F]RO948, and [18F]PI-2620. Each color represents one rater pair. Cohen's κ for five-level classification is weighted; all other κ values are unweighted.

© 2026 Ruppert E et al; HEAD study group. *JAMA Neurology*.

eFigure 13. Rater Confidence in Visual Reads

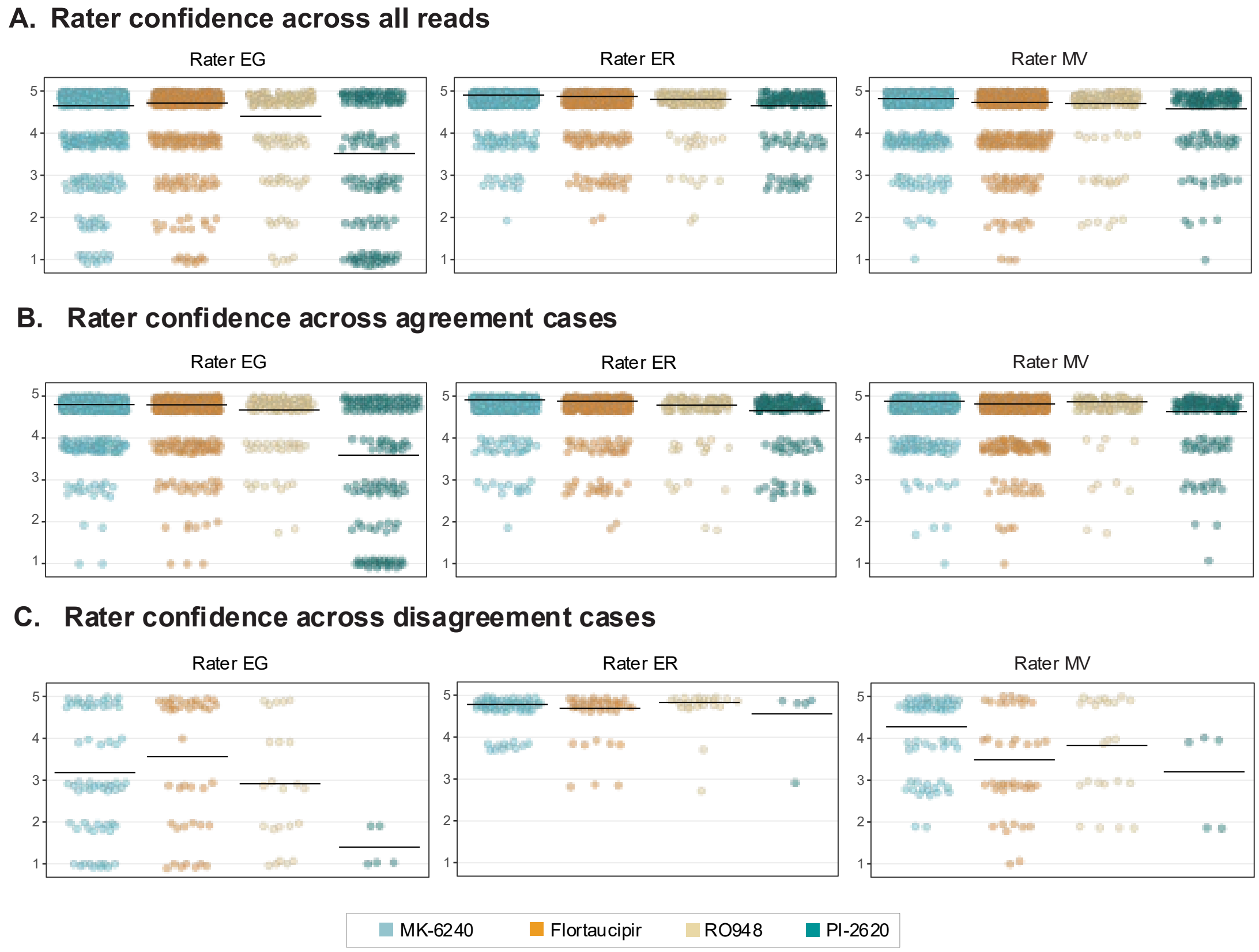

Dot plots show individual confidence scores assigned by each rater (E.G., E.R., M.V.) for each tracer. Horizontal bars indicate the mean. Panel A, rater confidence across all reads. Panel B, rater confidence restricted to cases where all three raters agreed. Panel C, rater confidence restricted to cases where at least one rater disagreed.

© 2026 Ruppert E et al; HEAD study group. *JAMA Neurology*.

eFigure 14. Patterns of Inter-Tracer Disagreements

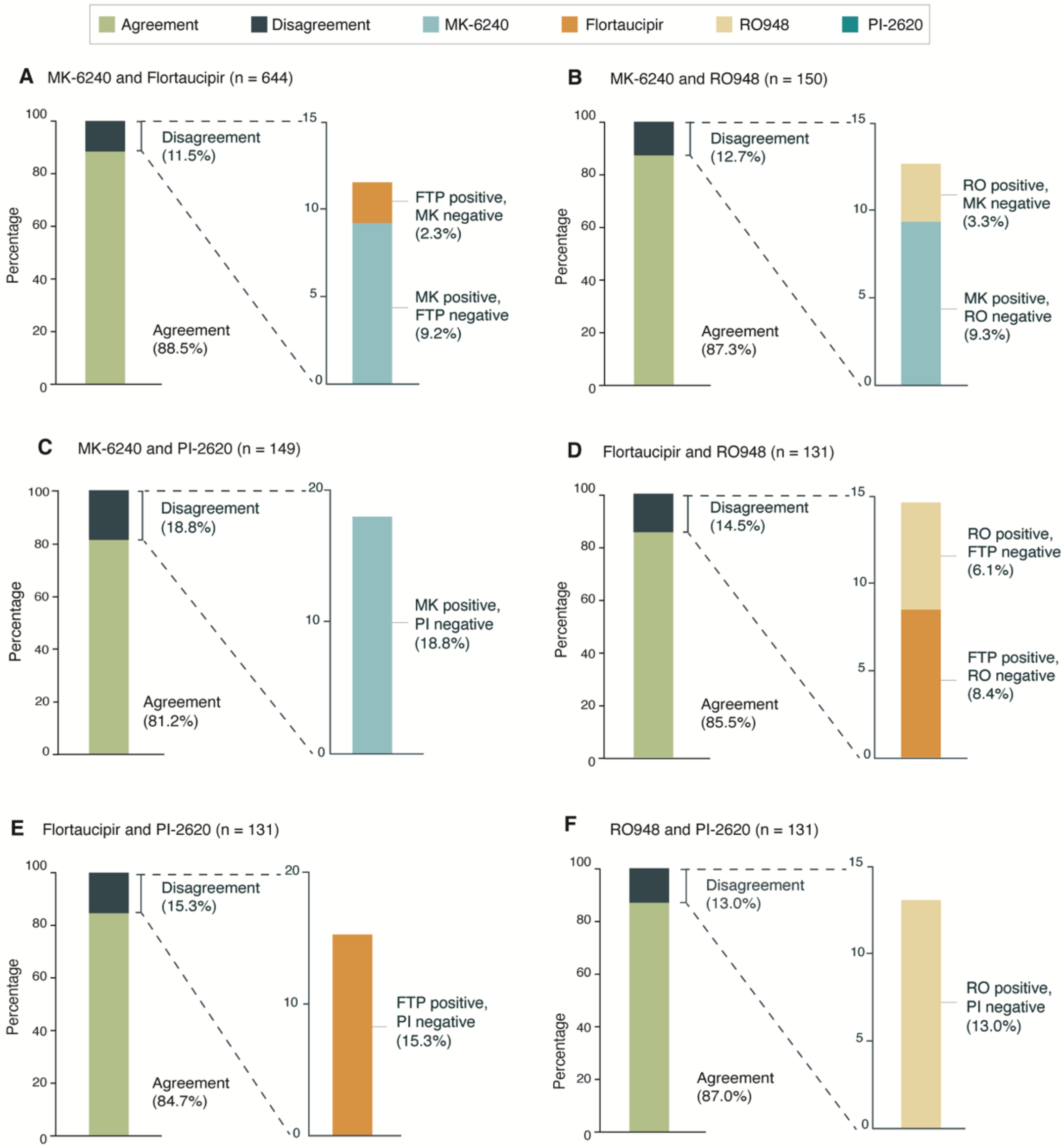

Each panel shows the overall binary agreement rate (left bar) and the breakdown of disagreements by direction (right bar) per tracer pair: Disagreement bars are color-coded by tracer. Panel A, [^{18}F]MK-6240 and [^{18}F]flortaucipir (n=644). Panel B, [^{18}F]MK-6240 and [^{18}F]RO948 (n=150). Panel C, [^{18}F]MK-6240 and [^{18}F]PI-2620 (n=149). Panel D, [^{18}F]flortaucipir and [^{18}F]RO948 (n=131). Panel E, [^{18}F]flortaucipir and [^{18}F]PI-2620 (n=131). Panel F [^{18}F]RO948 and [^{18}F]PI-2620 (n=131).

© 2026 Ruppert E et al; HEAD study group. *JAMA Neurology*.

eFigure 15. Discordant Cases Where Harmonized [18F]MK-6240 Was Positive and Harmonized [18F]flortaucipir Was Negative

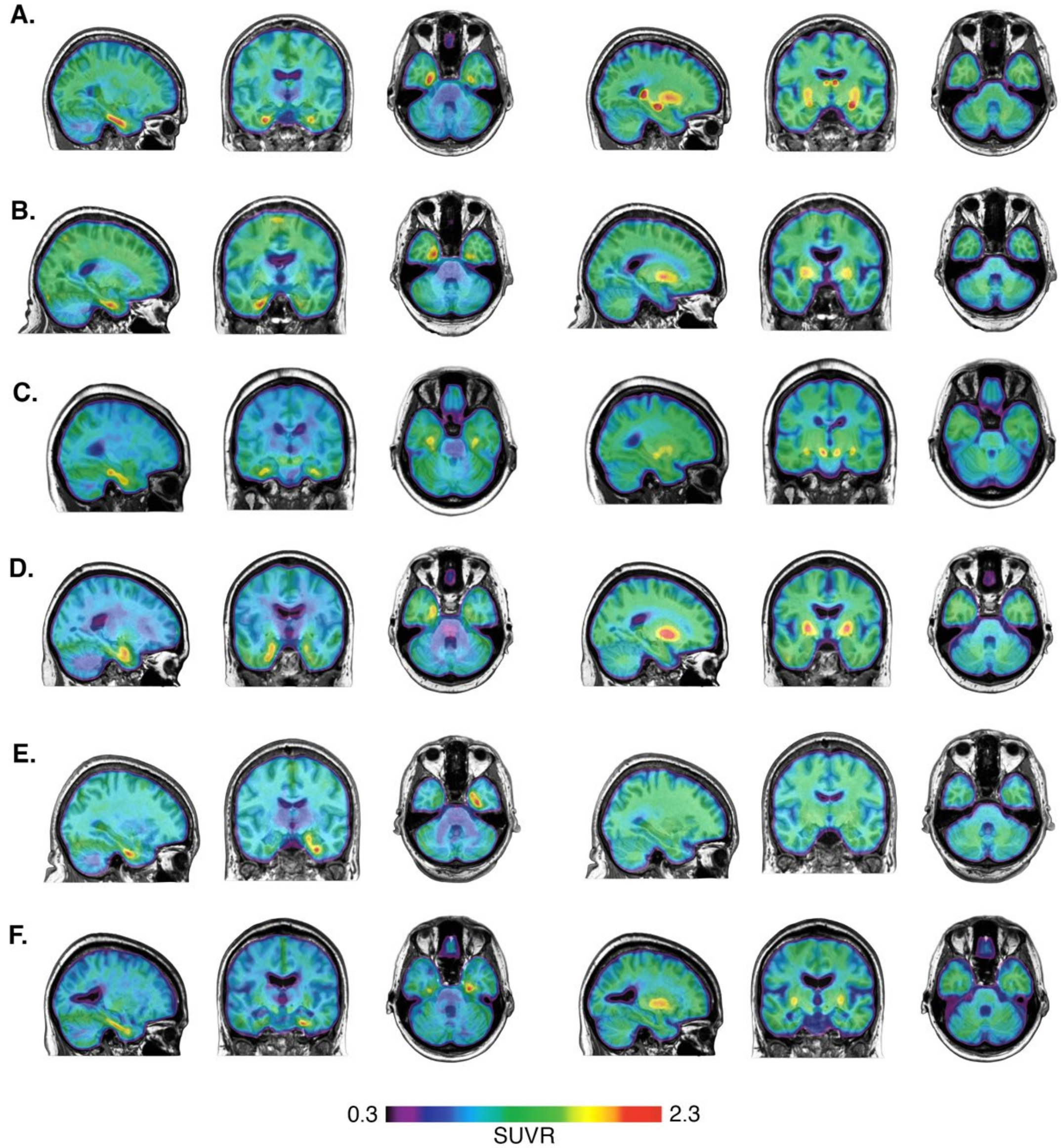

Abbreviations: Aβ, amyloid-β; CDR-SB, Clinical Dementia Rating sum of boxes; CU, cognitively unimpaired; MCI, mild cognitive impairment; MoCA, Montreal Cognitive Assessment; SUVR, standardized uptake value ratio.

Each panel shows [18F]MK-6240 (left) and [18F]flortaucipir (right) images from the same participant. All cases were classified as low tau burden on [18F]MK-6240 and negative on [18F]flortaucipir. Panel A, 74-year-old male; CU, Aβ-negative; MoCA 30; CDR-SB 0; Centiloid -0.1. Panel B, 81-year-old male; MCI, Aβ-negative; MoCA 20; CDR-SB 1; Centiloid 5.8. Panel C, 82-year-old male; dementia, Aβ-negative; MoCA missing; CDR-SB 7; Centiloid 6.8. Panel D, 75-year-old female; CU, Aβ-positive; MoCA 24; CDR-SB 0; Centiloid 140.0. Panel E, 61-year-old male; MCI, Aβ-positive; MoCA 23; CDR-SB 3.5; Centiloid 47.8. Panel F, 73-year-old male; dementia, Aβ-positive; MoCA 19; CDR-SB 5; Centiloid 61.5.

© 2026 Ruppert E et al; HEAD study group. *JAMA Neurology*.

eFigure 16. Discordant Cases Where Harmonized [^{18}F]MK-6240 Was Negative and Harmonized [^{18}F]flortaucipir Was Positive

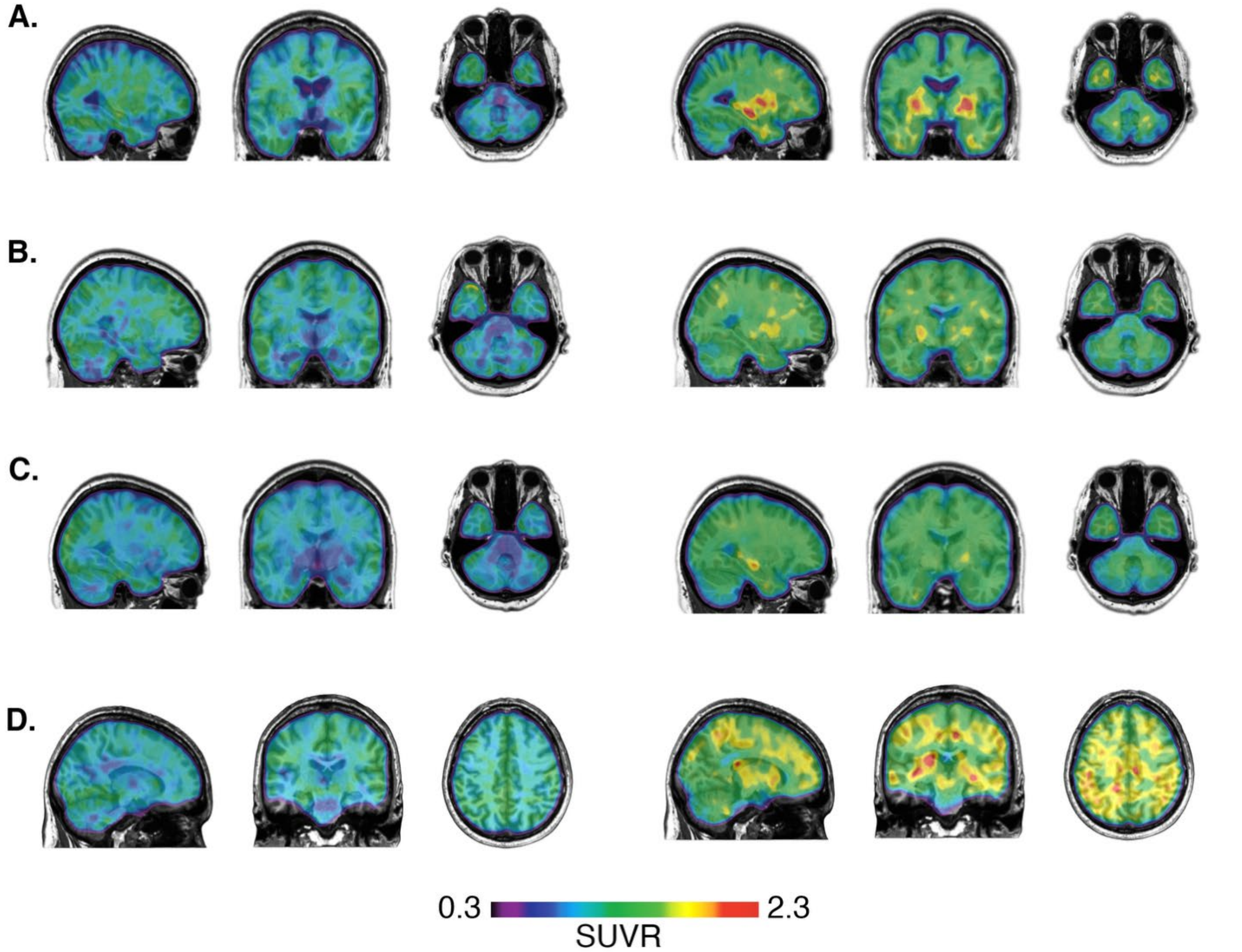

Abbreviations: Aβ, amyloid-β; CU, cognitively unimpaired; MCI, mild cognitive impairment; MoCA, Montreal Cognitive Assessment; CDR-SB, Clinical Dementia Rating Sum of Boxes; SUVR, standardized uptake value ratio.

Each panel shows [^{18}F]MK-6240 (left) and [^{18}F]flortaucipir (right) images from the same participant. Panels A, B, and C were classified as negative on [^{18}F]MK-6240 and low tau burden on [^{18}F]flortaucipir. Panel D was classified as negative on [^{18}F]MK-6240 and high tau burden on [^{18}F]flortaucipir. No dementia cases had a positive [^{18}F]flortaucipir and negative [^{18}F]MK-6240 read. Panel A, 75-year-old male; CU, Aβ-negative; MoCA 28; CDR-SB 0; Centiloid 4.3. Panel B, 60-year-old female; MCI, Aβ-negative; MoCA 29; CDR-SB 1; Centiloid 9.6. Panel C, 68-year-old female; CU, Aβ-positive; MoCA 29; CDR-SB 0.5; Centiloid 20.4. Panel D, 77-year-old male; MCI, Aβ-positive; MoCA 26; CDR-SB 0.5; Centiloid 24.2.

© 2026 Ruppert E et al; HEAD study group. *JAMA Neurology*.

eFigure 17. Atypical Tau Burden Examples With Corresponding Radiotracer Images in Individual Participants

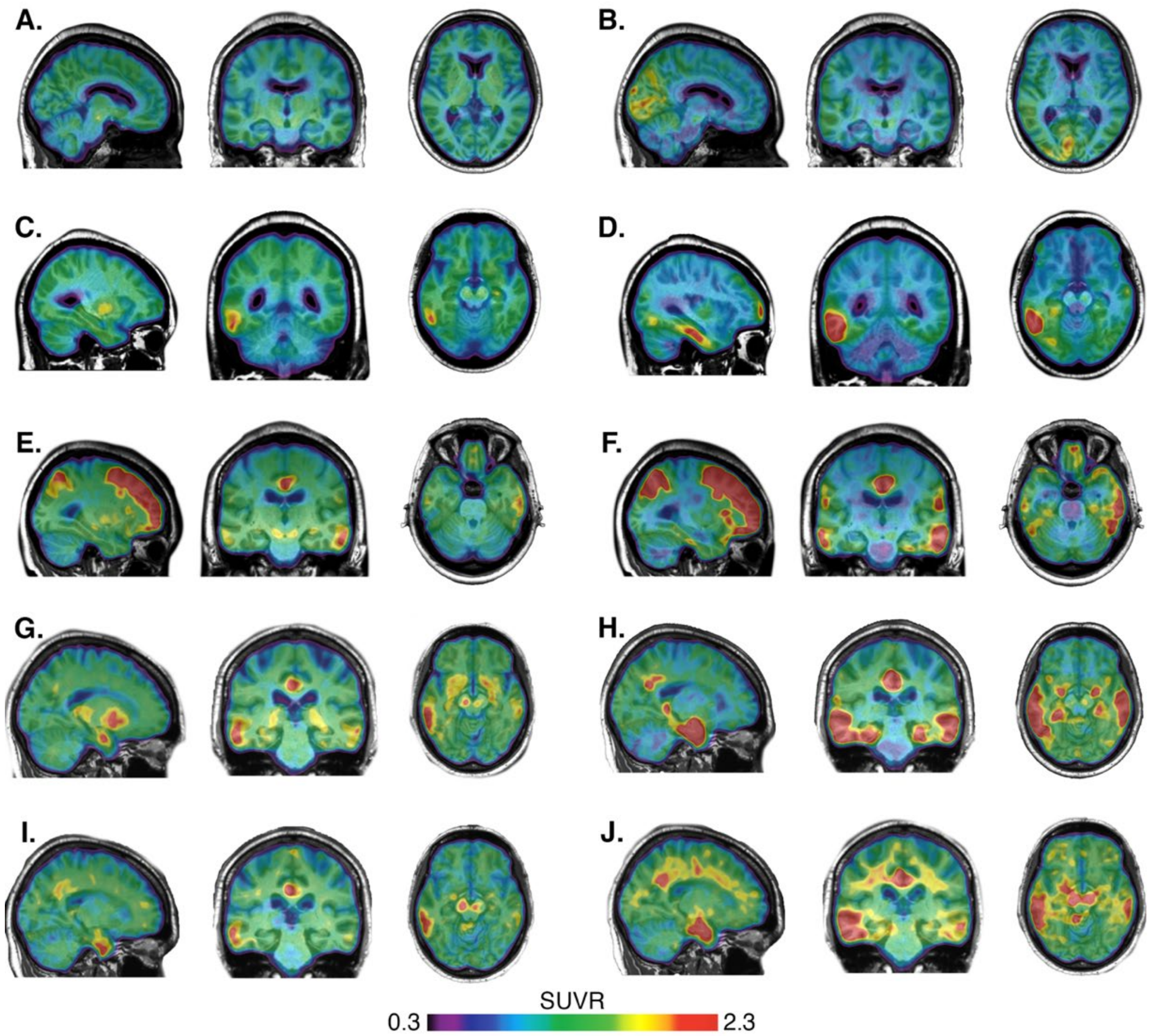

Abbreviations: Aβ, amyloid-β; CDR-SB, Clinical Dementia Rating Sum of Boxes; CU, cognitively unimpaired; MCI, mild cognitive impairment; MoCA, Montreal Cognitive Assessment; SUVR, standardized uptake value ratio.

Representative cases in which one radiotracer classified a participant as atypical tau burden while other radiotracers assigned a different classification for the same individual. In all cases, the atypical classification occurred with only one tracer. Panel A [^{18}F]flortaucipir (negative) and panel B [^{18}F]MK-6240 (atypical) from a 70-year-old male with MCI Aβ-negative (MoCA, 25; CDR-SB, 0.5; Centiloid, -4.3). Panel C [^{18}F]flortaucipir (atypical) and panel D [^{18}F]MK-6240 (moderate) from an 84-year-old female with MCI Aβ-positive (MoCA, 19; CDR-SB, 2.5; Centiloid, 55.7). Panel E [^{18}F]flortaucipir (atypical) and panel F [^{18}F]MK-6240 (high) from a 61-year-old female, CU Aβ-positive (MoCA, 27; CDR-SB, 0; Centiloid, 165.2). Panel G [^{18}F]flortaucipir (high), panel H [^{18}F]MK-6240 (high), panel I [^{18}F]PI-2620 (atypical), and panel J [^{18}F]RO948 (high) from a 68-year-old male with MCI Aβ-positive (MoCA, 24; CDR-SB, 2.0; Centiloid, 141.3).

© 2026 Ruppert E et al; HEAD study group. *JAMA Neurology*.

eFigure 18. Frequencies of 5-Level Tau Burden Classification Across the Four Tau PET Radiotracers

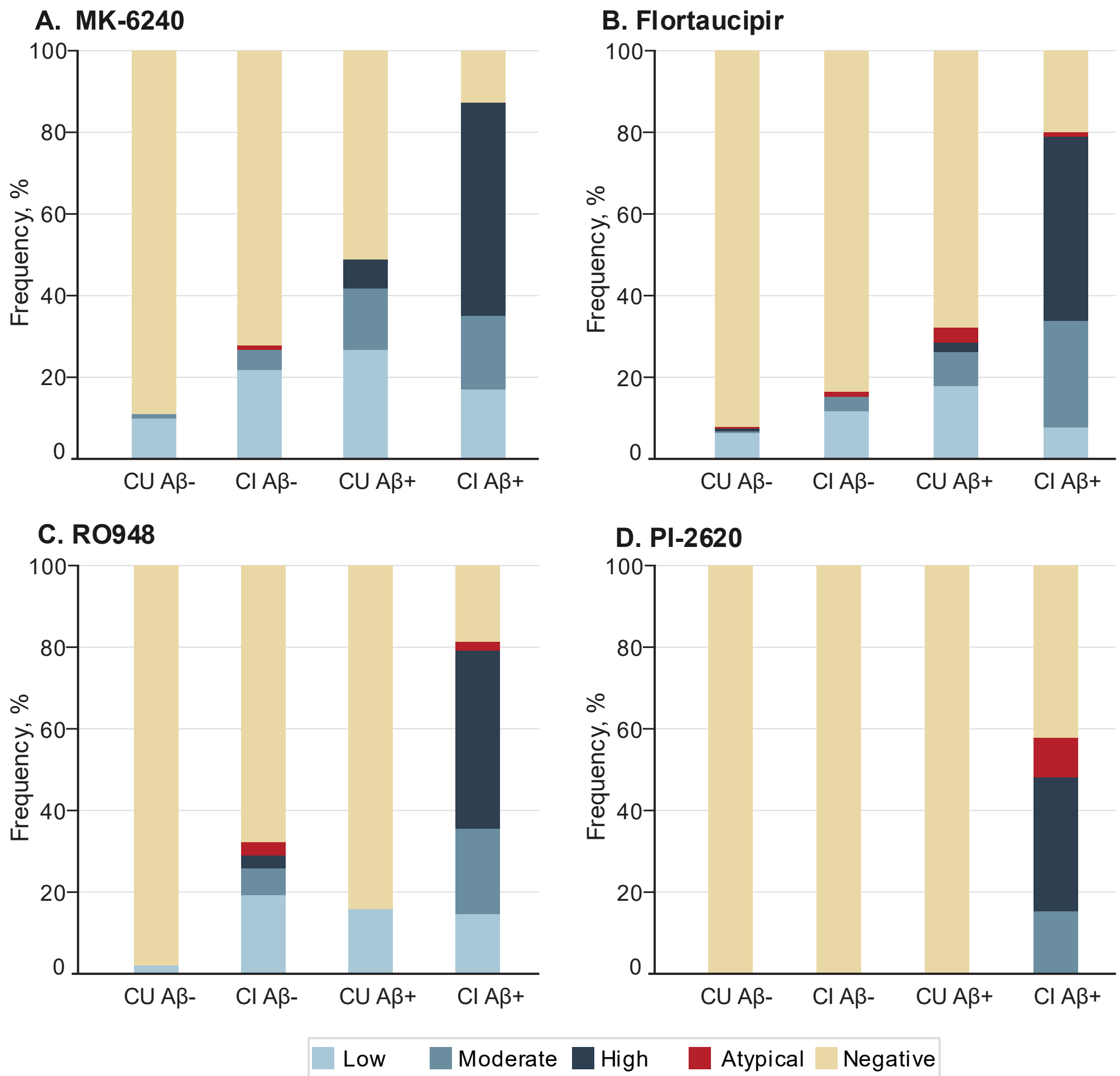

Abbreviations: Aβ, amyloid-β; CI, cognitively impaired; CU, cognitively unimpaired; PET, positron emission tomography. Distribution of harmonized five-level tau burden classification (negative, atypical, low, moderate, and high) stratified by clinical diagnosis (CU and CI) and amyloid-β status (Aβ+ vs Aβ−) for Panel A, [^{18}F]MK-6240 (n=681); Panel B, [^{18}F]flortaucipir (n=644); Panel C, [^{18}F]RO948 (n=150); and Panel D, [^{18}F]PI-2620 (n=149). CI includes participants with mild cognitive impairment and dementia. For [^{18}F]PI-2620, composite 1 was excluded from classification, which removed the low burden category for this tracer. Classification was based on majority vote across three raters.

© 2026 Ruppert E et al; HEAD study group. *JAMA Neurology*.

eFigure 19. Frequencies of Clinical Diagnoses in Each of the 5-Level Tau Burden Classification Across the Four Tau PET Radiotracers

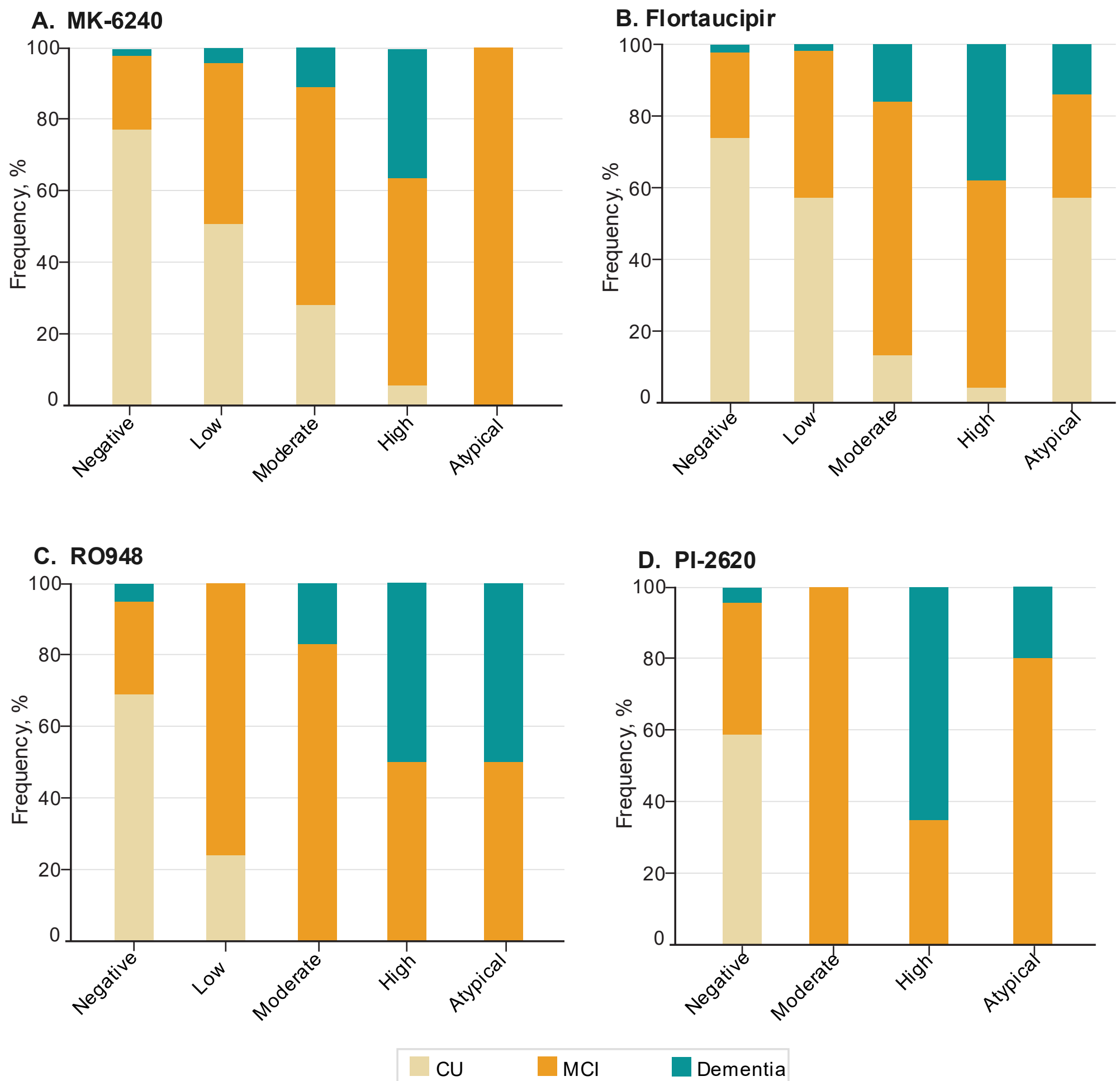

Abbreviations: CU, cognitively unimpaired; MCI, mild cognitive impairment; PET, positron emission tomography.
Distribution of clinical diagnoses across the harmonized five-level tau burden classification (negative, atypical, low, moderate, and high) for Panel A, [^{18}F]MK-6240 (n=681); Panel B, [^{18}F]flortaucipir (n=644); Panel C, [^{18}F]RO948 (n=150); and Panel D, [^{18}F]PI-2620 (n=149). For [^{18}F]PI-2620, composite 1 was excluded from classification, which removed the low burden category for this tracer. Classification was based on majority vote across three raters.

© 2026 Ruppert E et al; HEAD study group. *JAMA Neurology*.

eFigure 20. Cognitive Performance, Amyloid-β Load, and Plasma p-tau217 With Binary Tau-Positivity Across Radiotracers and Frameworks

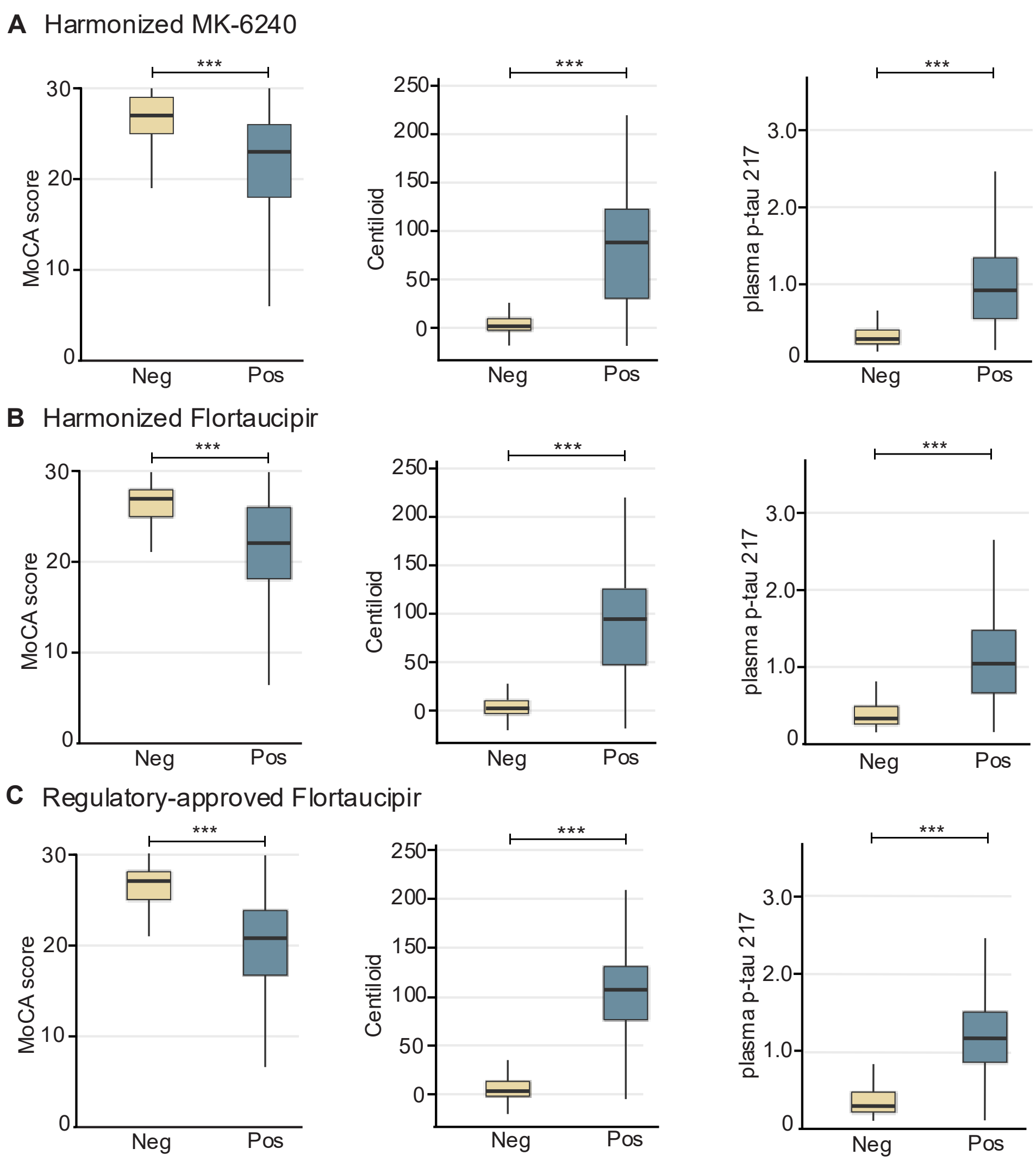

Abbreviations: CI, confidence interval; MoCA, Montreal Cognitive Assessment; PET, positron emission tomography

Box plots show MoCA scores, Centiloid values, and plasma p-tau217 levels stratified by binary tau positivity for Panel A, [^{18}F]MK-6240 with the harmonized framework (n = 381 negative, n = 263 positive); Panel B, [^{18}F]flortaucipir with the harmonized framework (n = 425 negative, n = 219 positive); and Panel C, [^{18}F]flortaucipir with the regulatory-approved framework (n = 454 negative, n = 171 positive). Boxes represent the interquartile range, horizontal lines indicate the median, whiskers extend to 1.5× the interquartile range, and individual data points are shown. Pairwise comparisons were estimated using linear models adjusted for age and sex. For harmonized [^{18}F]MK-6240 reads (Panel A), tau-positive participants had lower MoCA scores (adjusted mean difference = 4.4; 95% CI, 3.7-5.1), higher Centiloid values (68.6; 95% CI, 61.9-75.3), and higher plasma p-tau217 (0.6; 95% CI, 0.6-0.7). For harmonized [^{18}F]flortaucipir reads (Panel B), tau-positive participants had lower MoCA scores (4.9; 95% CI, 4.2-5.6), higher Centiloid values (70.9; 95% CI, 64.2-77.6), and higher plasma p-tau217 (0.7; 95% CI, 0.6-0.8). For regulatory-approved [^{18}F]flortaucipir reads (Panel C), tau-positive participants had lower MoCA scores (6.0; 95% CI, 5.3-6.7), higher Centiloid values (84.2; 95% CI, 77.8-90.5), and higher plasma p-tau217 (0.8 95% CI, 0.8-0.9). Missing: MoCA n=3, plasma p-tau217 n=11. *P < .05; **P < .01; ***P < .001. .

© 2026 Ruppert E et al; HEAD study group. *JAMA Neurology*.

eFigure 21. Discordant Tau PET Positivity Between Harmonized and Regulatory-Approved Visual Reading Frameworks

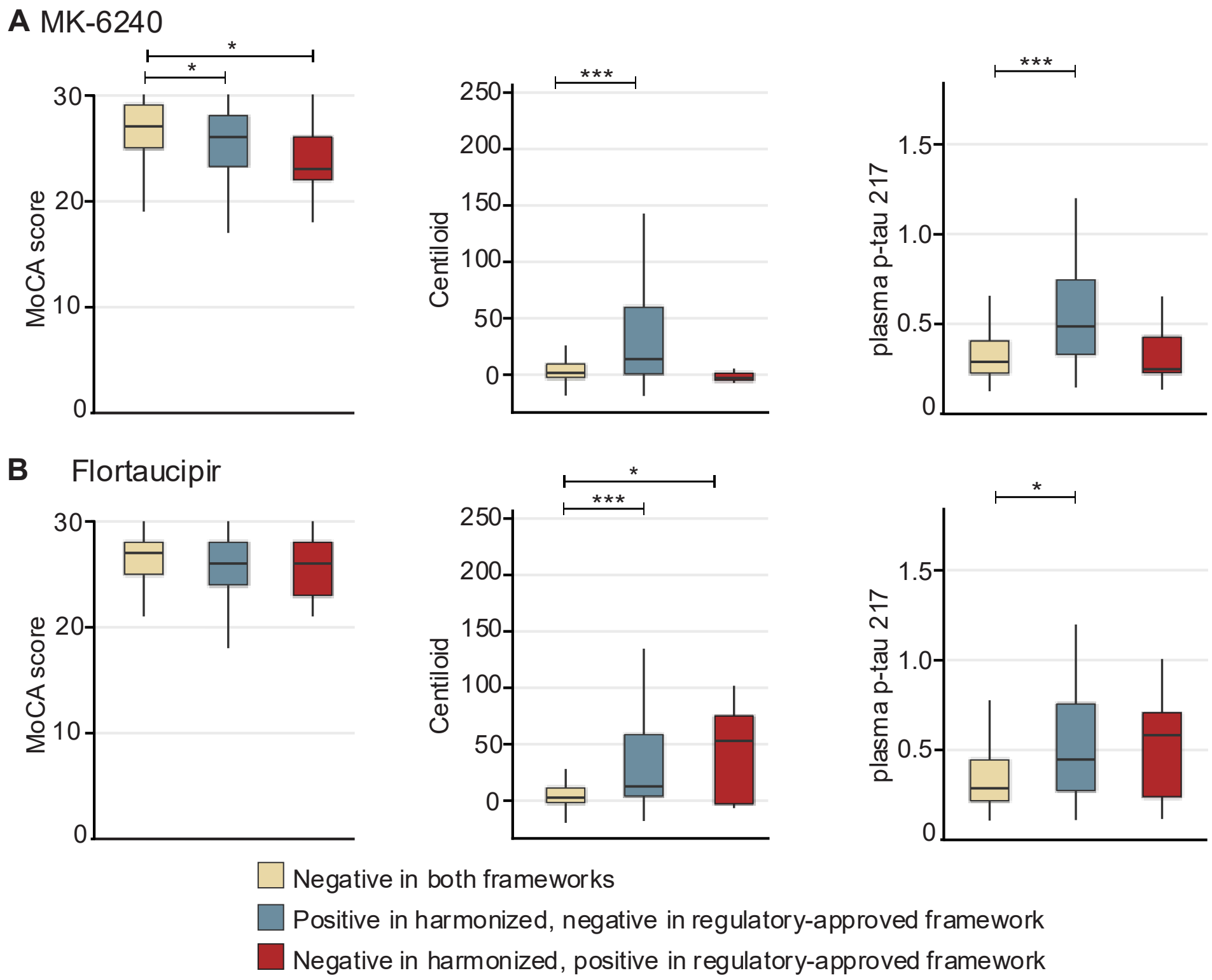

Abbreviations: MoCA, Montreal Cognitive Assessment; PET, positron emission tomography.

Box plots show MoCA scores, Centiloid values, and plasma p-tau217 for three groups: negative with both frameworks (yellow), positive only with the harmonized framework (blue), and positive only with the regulatory-approved [^{18}F]flortaucipir framework (red). Panels show results for harmonized [^{18}F]MK-6240 vs regulatory-approved [^{18}F]flortaucipir (A) and harmonized [^{18}F]flortaucipir vs regulatory-approved [^{18}F]flortaucipir (B). Box plots show medians with interquartile ranges; whiskers extend to 1.5 times the interquartile range. Adjusted mean differences (vs negative in both frameworks) from linear models adjusted for age and sex are reported below. In panel A, participants positive only on harmonized [^{18}F]MK-6240 had lower MoCA scores (-0.8; P=.04), higher Centiloid values (+20.3; P<.001), and higher plasma p-tau217 (+0.14 pg/mL; P<.001). Participants positive only on regulatory-approved [^{18}F]flortaucipir had lower MoCA scores (-2.6; P=.03) but no significant differences in Centiloid (-8.3; P=.68) or plasma p-tau217 (−0.05 pg/mL; P=.80). In panel B, participants positive only on harmonized [^{18}F]flortaucipir had higher Centiloid values (+15.7; P<.001) and plasma p-tau217 (+0.10 pg/mL; P=.01) but no significant MoCA difference (-0.3; P=.69). Participants positive only on regulatory-approved [^{18}F]flortaucipir had higher Centiloid values (+24.2; P=.01) but no significant differences in MoCA (−0.3; P=.89) or plasma p-tau217 (+0.16 pg/mL; P=.06). *P<.05; ***P<.001.

© 2026 Ruppert E et al; HEAD study group. *JAMA Neurology*.

eReferences.

1.Arani A, Borowski B, Felmlee J, et al. Design and validation of the ADNI MR protocol. *Alzheimers Dement*. Sep 2024;20(9):6615–6621. doi:10.1002/alz.14162

2. Weintraub, S., Besser, L., Dodge, H. H., et. al. Version 3 of the Alzheimer Disease Centers' Neuropsychological Test Battery in the Uniform Data Set (UDS). *Alzheimer Dis Assoc Disord*, Sep 2018*;32*(1), 10–17. doi 10.1097/WAD.0000000000000223

© 2026 Ruppert E et al; HEAD study group. *JAMA Neurology*.
