# Supplementary material for: A Harmonized Visual Reading Framework for Tau PET Staging in Alzheimer Disease
Source: JAMA Neurol. 2026 Sep 21:e263253. Online ahead of print. doi: 10.1001/jamaneurol.2026.3253 (PMC13595235; doi:10.1001/jamaneurol.2026.3253)
Supplement: Supplementary file 2 [file jamaneurol-e263253-s002.pdf]

Supplemental Online Content: Nonauthor Collaborators
*First name, last name, and suffix (if applicable) are required and will appear in PubMed.

| *Group Name(s): HEAD study | | | | | | | |
|---|---|---|---|---|---|---|---|
| ***First Name and Middle Initial(s)** | ***Last Name** | ***Suffix (eg, Jr, III)** | Academic Degrees | Institution | Location (city, state/province, country) | Role or Contribution, eg, chair, principal investigator | Group (if more than 1 Group listed in the byline) and/or Subgroup (eg, Steering Committee) |
| Cristiano S. | Aguzzoli | | MD | Brain Institute of Rio Grande do Sul, Pontifícia Universidade Católica do Rio Grande do Sul | | | |
| Selma | Avdagic | | MS | Washington University in St.Louis, School of Medicine, Mallinckrodt Institute of Radiology | | | |
| Tevy | Chan | | MD, MS | Centre intégré universitaire de santé et services sociaux de l'Ouest-de-l'Île-de-Montréal, Douglas Hospital Research Centre, McGill University Research Centre for Studies in Aging, Translational Neuroimaging Laboratory | | | |
| Ann D. | Cohen | | PhD | University of Pittsburgh, School of Medicine, Department of Psychiatry | | | |
| Emerine | Cummings | | MS | Washington University in St.Louis, School of Medicine, Mallinckrodt Institute of Radiology | | | |
| Vladimir | Fonov | | PhD | McGill University, Faculty of Medicine and Health Sciences, Department of Neurology & Neurosurgery; McGill University, Montreal Neurological Institute, McConnell Brain Imaging Centre | | | |
| Tatiana | Foroud | | PhD | Indiana University School of Medicine, Department of Medical and Molecular Genetics | | | |

Supplemental Online Content: Nonauthor Collaborators
*First name, last name, and suffix (if applicable) are required and will appear in PubMed.

| *First Name and Middle Initial(s) | *Last Name | *Suffix (eg, Jr, III) | Academic Degrees | Institution | Location (city, state/province, country) | Role or Contribution, eg, chair, principal investigator | Group (if more than 1 Group listed in the byline) and/or Subgroup (eg, Steering Committee) |
|---|---|---|---|---|---|---|---|
| Danielle | Gray | | MS | Washington University in St.Louis, School of Medicine, Mallinckrodt Institute of Radiology | | | |
| Xiaoyang | Hu | | PhD | Brown University, Departments of Psychiatry and Human Behavior & Cognitive and Psychological Sciences | | | |
| Yasser | Iturria-Medina | | PhD | McGill University, Faculty of Medicine and Health Sciences, Department of Neurology & Neurosurgery; McGill University, Montreal Neurological Institute, McConnell Brain Imaging Centre | | | |
| Wan Lu | Jia | | MD, MS | Centre intégré universitaire de santé et services sociaux de l'Ouest-de-l'Île-de-Montréal, Douglas Hospital Research Centre, McGill University Research Centre for Studies in Aging, Translational Neuroimaging Laboratory | | | |
| Jesse | Klostranec | | MD, PhD | McGill University, Faculty of Medicine and Health Sciences, Department of Diagnostic Radiology; McGill University, Montreal Neurological Institute, McConnell Brain Imaging Centre | | | |
| Douglas T. | Leffa | | MD, PhD | University of Pittsburgh, School of Medicine, Department of Psychiatry | | | |

Supplemental Online Content: Nonauthor Collaborators
*First name, last name, and suffix (if applicable) are required and will appear in PubMed.

| *First Name and Middle Initial(s) | *Last Name | *Suffix (eg, Jr, III) | Academic Degrees | Institution | Location (city, state/province, country) | Role or Contribution, eg, chair, principal investigator | Group (if more than 1 Group listed in the byline) and/or Subgroup (eg, Steering Committee) |
|---|---|---|---|---|---|---|---|
| Maxime | Montembault | | PhD | McGill University, Faculty of Medicine and Health Sciences, Department of Psychiatry; Centre intégré universitaire de santé et services sociaux de l'Ouest-de-l'Île-de-Montréal, Douglas Hospital Research Centre, McGill University Research Centre for Studies in Aging | | | |
| Karine | Provost | | MD, MS | McGill University, Montreal Neurological Institute, McConnell Brain Imaging Centre; Centre hospitalier de l'Université de Montréal, Faculty of Medicine, Department of Radiology, Radiation Oncology and Nuclear Medicine | | | |
| Nesrine | Rahmouni | | MS | Centre intégré universitaire de santé et services sociaux de l'Ouest-de-l'Île-de-Montréal, Douglas Hospital Research Centre, McGill University Research Centre for Studies in Aging, Translational Neuroimaging Laboratory | | | |
| Pampa | Saha | | PhD | University of Pittsburgh, School of Medicine, Department of Psychiatry | | | |
| Juli | Singer | | MS | University of Pittsburgh, School of Medicine, Department of Psychiatry | | | |

Supplemental Online Content: Nonauthor Collaborators
*First name, last name, and suffix (if applicable) are required and will appear in PubMed.

| ***First Name and Middle Initial(s)** | ***Last Name** | ***Suffix (eg, Jr, III)** | Academic Degrees | Institution | Location (city, state/province, country) | Role or Contribution, eg, chair, principal investigator | Group (if more than 1 Group listed in the byline) and/or Subgroup (eg, Steering Committee) |
|---|---|---|---|---|---|---|---|
| Jenna | Stevenson | | BS | Centre intégré universitaire de santé et services sociaux de l'Ouest-de-l'Île-de-Montréal, Douglas Hospital Research Centre, McGill University Research Centre for Studies in Aging, Translational Neuroimaging Laboratory | | | |
| Victoria | Tate | | MS | Washington University in St.Louis, School of Medicine, Mallinckrodt Institute of Radiology | | | |
| Arthur W. | Toga | | PhD | University of Southern California, Mark and Mary Stevens Neuroimaging and Informatics Institute | | | |
| Raphael L. | Tuma | | MD, PhD | University of Pittsburgh, School of Medicine, Department of Psychiatry | | | |
| Paolo | Vitali | | PhD | Centre intégré universitaire de santé et services sociaux de l'Ouest-de-l'Île-de-Montréal, Douglas Hospital Research Centre, McGill University Research Centre for Studies in Aging | | | |
