# Supplementary material for: A Harmonized Visual Reading Framework for Tau PET Staging in Alzheimer Disease
Source: JAMA Neurol. 2026 Sep 21:e263253. Online ahead of print. doi: 10.1001/jamaneurol.2026.3253 (PMC13595235; doi:10.1001/jamaneurol.2026.3253)
Supplement: Supplementary file 3 [file jamaneurol-e263253-s003.pdf]

Data Sharing Statement

Ruppert. A Harmonized Visual Reading Framework for Tau PET Staging in Alzheimer Disease. *JAMA Neurol*. Published September 21, 2026. doi:10.1001/jamaneurol.2026.3253

Data
Data available: Yes
Data types: Deidentified participant data
How to access data: upon request: pascoalt@upmc.edu
When available: With publication

Supporting Documents
Document types: Statistical/analytic code
How to access documents: upon request: pascoalt@upmc.edu
When available: With publication

Additional Information
Who can access the data: researchers whose proposed use of the data has been approved
Types of analyses: any purpose
Mechanisms of data availability: after approval of a proposal
